# Benzo[*cd*]azulenyl: A Structural Isomer of Phenalenyl—Synthesis and Properties of Its Tri‐*tert*‐butyl‐substituted Derivative and Formation of a Thermal‐ and Photoresponsive σ‐Dimer

**DOI:** 10.1002/chem.70947

**Published:** 2026-03-26

**Authors:** Kaho Takeuchi, Akihito Konishi, Makoto Yasuda

**Affiliations:** ^1^ Department of Applied Chemistry, Graduate School of Engineering The University of Osaka Suita Osaka Japan; ^2^ Innovative Catalysis Science Division, Institute For Open and Transdisciplinary Research Initiatives (ICS‐OTRI) The University of Osaka Suita Osaka Japan

**Keywords:** azulene, C─C bond formation, nonalternant hydrocarbon, photochromism, radical

## Abstract

The chemistry of nonalternant hydrocarbons has recently experienced a significant resurgence in interest. While extensive research has been conducted on azulenoids, that is, structural isomers of benzenoid polycyclic hydrocarbons, the exploration of doublet open‐shell nonalternant systems, that is, isomers of open‐shell graphene nanofragments has not yet been undertaken. To clarify the chemical and physical effects of nonhexagonal rings on the unpaired electron in these structures, we focus on benzo[*cd*]azulenyl, which is a nonalternant isomer of phenalenyl. We synthesized and characterized a tri‐*tert*‐butyl substituted derivative, which exists as the stable σ‐dimer in the solid state. In solution, the σ‐bond of the dimer dissociates in response to external stimuli (light and heat), yielding the monomeric radical. Analyzing the bond‐dissociation and recombination processes allowed us to determine the thermodynamic and kinetic parameters. Unlike phenalenyl, the spin density of benzo[*cd*]azulenyl is unevenly distributed, skewing toward its five‐ and seven‐membered rings. This asymmetrical spin density, combined with kinetic protection, grants the tri‐*tert*‐butyl substituted derivative selective and reversible C─C‐bond‐formation properties. The nonalternant nature of benzo[*cd*]azulenyl enhances its redox properties and lowers the photoexcitation energy. Our study contributes to the establishment of design strategies for novel open‐shell doublet radical materials based on nonalternant hydrocarbon frameworks.

## Graphical abstract

Benzo[*cd*]azulenyl, a nonalternant isomer of phenalenyl, is identified as a new member of the nonalternant hydrocarbon radical family. The tri‐*tert*‐butyl substituted derivative forms a stable σ‐dimer in the solid state. Upon exposure to external stimuli such as light and heat, the σ‐bond of the dimer dissociates, producing the monomeric radical. This research advances the development of design strategies for novel open‐shell doublet radical materials utilizing nonalternant hydrocarbon frameworks.

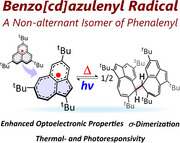

## 1 Introduction

**FIGURE 1:**
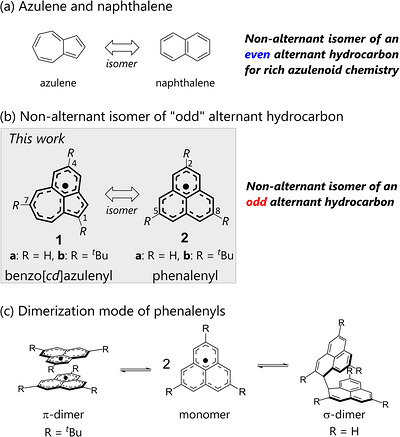
(a) Chemical structures of azulene and naphthalene. (b) Chemical structure of the benzo[*cd*]azulenyl radical **1**, a structural nonalternant isomer of phenalenyl radical **2**. (c) Dimerization mode of phenalenyl radicals.

The recent resurgence of nonalternant hydrocarbons [1, 2, 3, 4, 5] has highlighted the importance of understanding the structure–property relationships in π‐conjugated networks [6], particularly the effect of incorporating nonhexagonal rings into π‐systems [7, 8, 9, 10]. The topology of the π‐conjugated networks in nonalternant systems, which stands in sharp contrast to that of alternant benzenoid systems, results in unique electron configurations and molecular orbital characteristics. The most fundamental and representative example of a nonalternant isomer of an alternant hydrocarbon with an even number of *sp* ^2^ carbons (referred to in this article as ‘even alternant hydrocarbons’) is azulene, which is a structural isomer of naphthalene (Figure 1a) [11, 12, 13, 14, 15, 16, 17, 18]. The 5/7‐membered bicyclic structure of azulene endows it with remarkable optoelectronic properties, including a large dipole moment (1.08 D) [19], a narrow HOMO–LUMO energy gap due to its poor spatial overlap [6, 20], and an *anti*‐Kasha emission [11, 21]. Recent advances in synthetic methodologies and characterization techniques have led to the creation of a variety of azulene‐based polycyclic systems, thus enabling the showcasing of their optoelectronic properties [22, 23, 24, 25, 26, 27]. However, despite extensive research on azulenoids, only a limited number of examples of open‐shell nonalternant systems that consist of an odd number of *sp* ^2^ carbons bearing an unpaired electron (referred to in this article as ‘odd nonalternant hydrocarbons’) are known to exist [28, 29, 30, 31, 32, 33, 34, 35, 36, 37]. Fluorenyl radicals [38, 39, 40] and their congeners [41, 42, 43, 44, 45] have been widely studied as representative odd nonalternant hydrocarbon radicals. Through the synthesis of various derivatives [39], including π‐extended analogues [41, 46, 47], the reactivity [38, 41] and optoelectronic properties [40, 48, 49] of these radicals have been elucidated. In sharp contrast to the abundance of research on fluorenyl‐based radicals, the exploration of nonalternant isomers of benzenoid mono‐radicals, which are important molecular models of doublet open‐shell graphene fragments [50, 51, 52], has yet to be undertaken. Moreover, the chemical and physical effects of nonhexagonal rings on the unpaired electron remain largely unknown.

In this context, we describe here the synthesis and characterization of a derivative of benzo[*cd*]azulenyl radical **1**, that is, a nonalternant isomer of phenalenyl radical **2** (Figure 1b). In radical **1**, a naphthalene substructure of **2** is replaced with an azulene core. Phenalenyl **2** is the simplest odd alternant hydrocarbon radical [53], featuring *D*_3_ *_h_* symmetry and a uniform distribution of spin density across the six *α*‐positions. Since its first preparation in the 1950s [54, 55], the chemistry of **2** has flourished. The electronic [56, 57, 58], structural [59, 60] and chemical properties [61, 62, 63, 64] of **2** and its derivatives have been extensively explored. One of the most intriguing aspects of **2** is its radical dimerization (Figure 1c) [65, 66, 67]. Depending on the substituents introduced, phenalenyl **2** can form either σ‐dimers [68, 69, 70] or π‐dimers [59, 71, 72]. The rapid growth of research on phenalenyl‐related molecules has been summarized well in several reviews [73, 74, 75, 76, 77]. In stark contrast to **2**, benzo[*cd*]azulenyl **1** remains an elusive synthetic target despite numerous efforts [78, 79] involving the syntheses of hydrogenated hydrocarbons [78, 79, 80, 81], ketones [82, 83, 84, 85, 86, 87] and ionic species [88, 89]. The first attempt to synthesize **1** was reported by Hafner in 1963, who characterized the anionic species [78]. Boekelheide later tried to synthesize **1a**, but his efforts resulted only in the formation of insoluble polymeric materials [79]. These pioneering studies clearly indicate that kinetic stabilization seems to be crucial for the synthesis of **1**. Theoretical calculations have provided fascinating insights into the spin–spin interactions between two molecules of **1**. Su predicted that **1a** could potentially form various types of pancake‐shaped π‐dimers [90]. These intriguing experimental and theoretical studies have peaked our interest in synthesizing and characterizing **1**.

**FIGURE 2:**
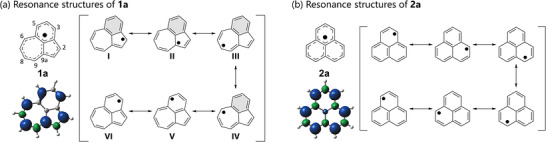
Canonical structures and spin‐density maps of (a) benzo[*cd*]azulenyl **1a**, where the hexagonal units that have a 6π‐benzenoid character are shown in gray and (b) phenalenyl **2a**. The spin‐density maps were calculated at the UB3LYP/6‐311+G(d,p) level.

Resonance structures reveal the differences in the electronic structures between **1a** and **2a** (Figure 2). Phenalenyl **2a** has six equivalent resonance structures, which demonstrate a symmetric delocalization of an unpaired electron across the six *α*‐positions, contributing to its thermodynamic stability (Figure 2b). In contrast, whilst benzo[*cd*]azulenenyl **1a** can also be represented by six canonical structures and the unpaired electron is delocalized across the peripheral π‐conjugated structure, these structures are not equivalent (Figure 2a). Given that the hexagonal ring exhibits greater local aromaticity than the azulene subunit, the four canonical structures (**I–IV**) with a 6π‐benzenoid character are expected to contribute more to the overall character of the molecule than the two remaining structures (**V** and **VI**). This notion is supported by the spin‐density map calculated at the UB3LYP/6‐311+G(d,p) level for **1a**, which indicates that the unpaired electron of **1a** is more likely to be distributed toward the peripheral 2‐, 6‐, 8‐, and 9a‐positions on the azulene subunit rather than the 3‐ and 5‐positions on the hexagonal unit. This unsymmetric distribution of the unpaired electron in **1a** enhances the local aromaticity and decreases the radical‐stabilization energy (RSE) relative to **2a**, which exhibits global aromaticity and a high RSE (Tables S12–S14). These considerations prompted us to synthesize **1** and to examine how the nonalternant skeleton influences the behavior of the unpaired electron by conducting a comparative analysis with phenalenyl radical **2**.

## 2 Results and Discussion

### 2.1 Synthesis and Characterization of the σ‐Dimer

**SCHEME 1:**
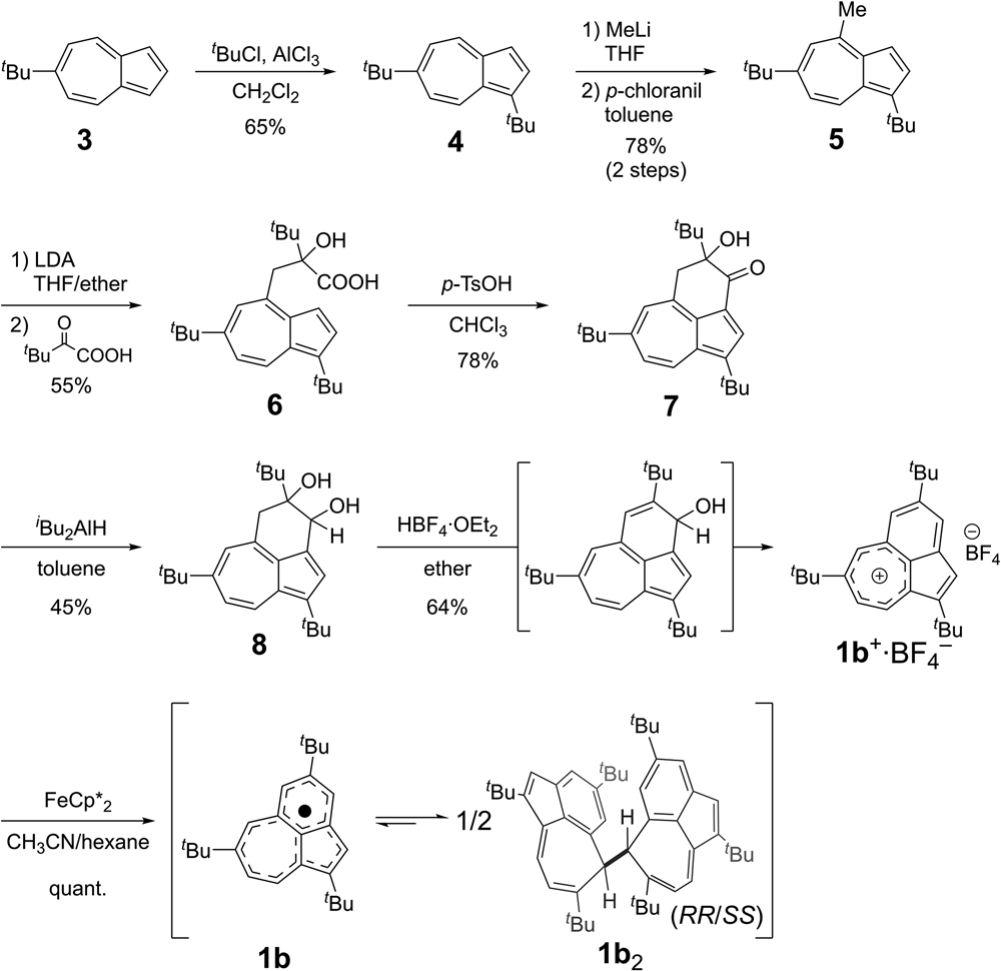
Synthesis of benzo[*cd*]azulenenyl **1b** and its σ‐dimerization.

In our pursuit of the successful synthesis and characterization of **1**, we designed 1,4,7‐tri‐*tert*‐butyl benzo[*cd*]azulenyl (**1b**; Figure 1b), as the 1‐, 4‐, and 7‐positions have such small spin‐density coefficients that we can minimize the substitution effect on the main core of **1**. Target **1b** is a structural isomer of phenalenyl derivative **2b**, which has been well‐characterized in terms of its optoelectronic properties [59] and dimerization processes [65, 91]. Scheme 1 illustrates the synthetic route to **1b**.

**FIGURE 3:**
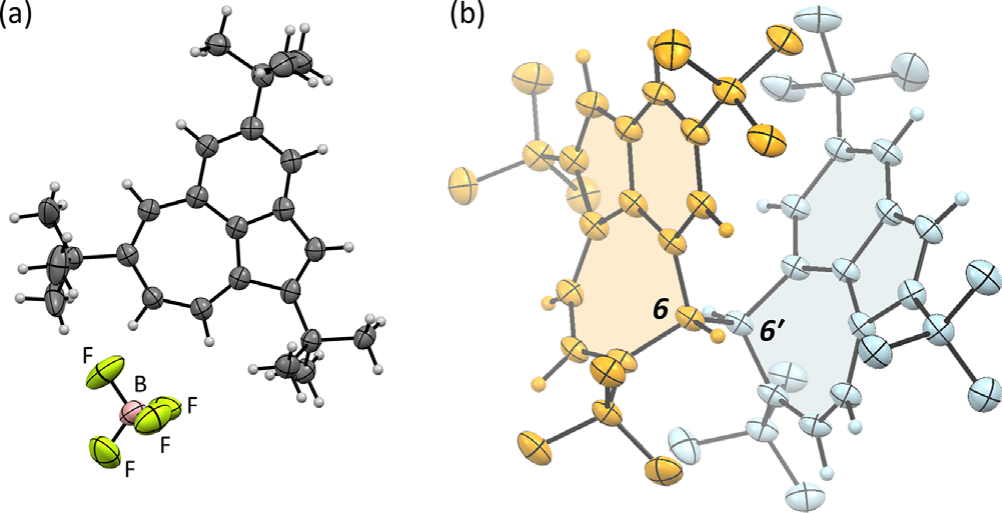
Molecular structures of (a) **1b** ^+^·BF_4_^−^ and (b) **1b**_2_ in the solid state with thermal ellipsoids at 50% probability; some hydrogen atoms are omitted for clarity.

Starting from 6‐(*tert*‐butyl)azulene (**3**) [92], the introduction of an additional *tert*‐butyl group at the 1‐position gave 1,6‐di‐*tert*‐butylazulene (**4**) [93]. 1,6‐Di‐*tert*‐butyl‐4‐methylazulene (**5**) was synthesized through the methylation of **4**. The construction of the remaining hexagonal ring was accomplished using the Yli‐Kauhaluoma procedure [94, 95]. After deprotonation of the methyl group at the 4‐position of **5** with LDA, treatment of the generated anion with 3,3‐dimethyl‐2‐oxobutyric acid gave *α*‐hydroxy‐carboxylic‐acid derivative **6**. The cyclization of **6** to tricyclic dihydrobenzo[*cd*]azulen‐3‐one (**7**) was achieved using *p*‐toluenesulfonic acid in CHCl_3_. The reduction of the carbonyl group of **7** with *^i^* Bu_2_AlH gave diol **8**. Treating diol **8** with HBF_4_·Et_2_O directly produced the salt **1b** ^+^·BF_4_^−^ as a stable, dark purple solid. The solid‐state structure of cation **1b** ^+^ was unequivocally established via a single‐crystal X‐ray diffraction analysis (Figure 3a), which showed the presence of a pronounced tropylium‐type character (Figure S4 and Table S12). Then, a one‐electron reduction of **1b** ^+^ with decamethylferrocene (FeCp*_2_) was carried out. Immediately after adding FeCp*_2_, the reaction produced a strongly colored solution that changed from purple to dark brown, indicating the formation of radical **1b**. However, over time, the color of the reaction mixture gradually faded to a pale yellow. The ^1^H NMR spectrum of the product, recorded at room temperature, showed clear and sharp NMR signals attributable to a single product, demonstrating the selective formation of a closed‐shell σ‐dimer. Recrystallization from benzene/ethanol yielded a single crystal suitable for X‐ray crystallography. The X‐ray diffraction analysis revealed that **1b** selectively dimerizes at the 6‐position of the heptagon, wherein the stereo‐configuration of the σ‐bonded carbon atoms is *RR*/*SS* (Figure 3b). The σ‐bond connecting the two **1b** units (1.581(3) Å; Figure S5) is longer than a typical C(*sp* ^3^)–C(*sp* ^3^) single bond (1.54 Å), but significantly shorter than the σ‐bonds observed in other reported σ‐dimers of phenalenyl radicals (generally >1.6 Å) [68, 69, 75]. This suggests that the σ‐bond of **1b**_2_ is stronger than those found in most phenalenyl radicals. In contrast to other reported σ‐dimers of hydrocarbon radicals [40, 41, 68, 96], melting or grinding solid **1b**_2_ gave no monomeric radical species. The carbon atom at the 6‐position of **1b** has the second‐highest spin density of the six possible carbon radical positions (Figure 2a), thus leading to the formation of **1b**_2_ via a radical–radical coupling. DFT calculations indicated that the obtained (6*R*,6′*R*)‐**1b**_2_ is the most stable regio‐ and stereoisomer compared to the other possible σ‐dimers (Table S34). The balance between the steric congestion from the three *tert*‐butyl groups and the structural flexibility of the carbon atom on the heptagon can be expected to facilitate the selective dimerization of **1b**. The σ‐dimer of **1b**_2_ is stable under ambient conditions and a series of ^1^H NMR measurements revealed no signal change upon exposure of **1b**_2_ to air under indoor light illumination at room temperature after 10 days (Figure S1).

### 2.2 Characterization of the Monomeric Radical and its Thermodynamic Equilibrium

**FIGURE 4:**
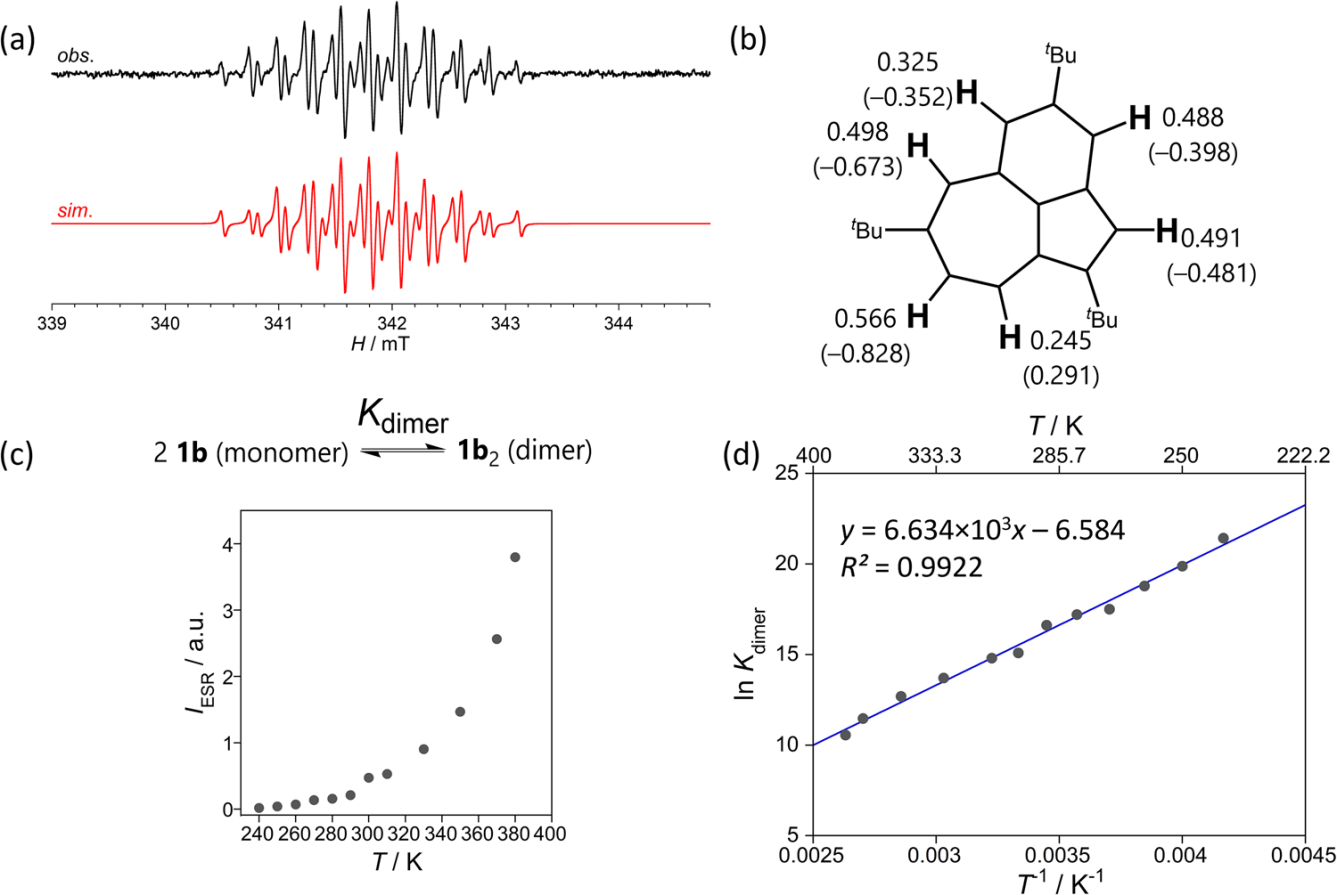
(a) Observed (black) and simulated (red) ESR spectra of a hexane solution of **1b**_2_ at 300 K. (b) Summary of the hyperfine coupling constants (|*a*_H_|/mT) for **1b**. The values in parentheses were calculated at the UB3LYP/EPR‐II//UB3LYP‐D3(BJ)/6‐311G(d) level. (c) The ESR signal intensity of **1b** as a function of temperature. (d) The dimerization constant *K*_dimer_ of **1b** as a function of temperature.

Dissolution of **1b**_2_ in hexane at room temperature gave a weak but well‐resolved multiline ESR signal (*g* = 2.0027) corresponding to the monomeric radical **1b** (Figure 4a). This observation indicates that radical **1b** and its σ‐dimer **1b**_2_ exist as an equilibrium mixture in dilute solution. The hyperfine coupling constants (hfccs) of the main core of **1b** were determined by computational simulation to be |*a*_H2_| = 0.491 mT, |*a*_H3_| = 0.488 mT, |*a*_H5_| = 0.325 mT, |*a*_H6_| = 0.498 mT, |*a*_H8_| = 0.566 mT, and |*a*_H9_| = 0.245 mT (Figure 4b). Upon cooling, the ESR signals decreased in intensity and almost disappeared at 240 K, due to the σ‐dimerization. At approximately room temperature, the equilibrium overwhelmingly favors σ‐dimer **1b**_2_, which explains the negligible radical effect seen in the NMR signals recorded at this temperature. Variable‐temperature (VT) ESR measurements determined that the enthalpy change (Δ*H*) and entropy change (Δ*S*) for this dimerization are −13 kcal·mol^−1^ and −13 cal·K^−1^·mol^−1^, respectively (Figure 4c). Table 1 summarizes the thermodynamic parameters of **1b** and those of some phenalenyls. The Δ*H* value of **1b** is more negative than that of the σ‐dimerization of pristine phenalenyl radical **2a** (Δ*H* = −9.8 kcal·mol^−1^; Table 1, entry 2) [97] and the π‐dimerization of tri‐*tert*‐butyl phenalenyl radical **2b** (Δ*H* = −9.5 kcal·mol^−1^; Table 1, entry 3) [65]. Notably, the Δ*H* value of **1b** is comparable to that of **2‐(C_6_F_5_)_3_** (Δ*H* = −15 kcal·mol^−1^; Table 1, entry 4) [68], in which face‐to‐face attractive interactions between the C_6_F_5_ group and the phenalenyl ring facilitate σ‐dimerization, indicating that **1b** also has a strong tendency to form dimers, even in the absence of polar substituents. The |Δ*S*| value of **1b** is much smaller than that of the π‐dimerization of **2b** (Δ*S* = −36 cal·K^−1^·mol^−1^; Table 1, entry 3) [65] and that of the σ‐dimerization of **2‐(C_6_F_5_)_3_** (Δ*S* = −32 cal·K^−1^·mol^−1^; Table 1, entry 4) [68], but comparable to that of the σ‐dimerization of **2a** (Δ*S* = −11 cal·K^−1^·mol^−1^; Table 1, entry 2) [97]. These results may reflect the greater structural flexibility of a conjugated heptagon compared to a hexagon, despite the steric congestion of the three *tert*‐butyl substituents. Based on these thermodynamic parameters, the Gibbs‐free‐energy change (Δ*G*) for the σ‐dimerization of **1b** was calculated to be −9.1 kcal·mol^−1^ at 298 K. This value was corroborated by the theoretical estimate of Δ*G* (−9.0 kcal·mol^−1^) obtained at the (U)B3LYP‐D3(BJ)/6‐311G(d)/SMD(toluene) level for the dimerization of **1b** to **1b**_2_ (Figure S28). When exposed to air, the intensity of the ESR spectrum of **1b** gradually diminished, and after one hour, it transformed into a different spectrum altogether (Figure S1b). The estimated half‐life for the decomposition process was found to be 30 min. The MALDI‐TOF mass spectrometry analysis of the decomposed sample revealed the formation of oxygen‐adducts in air (Figure S1c).

**TABLE 1:** Thermodynamic parameters for the σ‐/π‐dimerization and optoelectronic properties of **1b** as well as of phenalenyls **2a**, **2b**, and **2‐(C_6_F_5_)_3_**.

Entry	Compound	Type of dimerization	Δ*H* / kcal·mol^−1^	Δ*S* / cal·K^−1^·mol^−1^	*λ*_max_ / nm (for monomeric radical)	*E*_red_^1/2^ / V *E*_ox_^1/2^ / V (vs. Fc/Fc^+^)
1	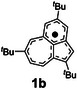	σ‐dimerization	−13	−13	850	−1.44 −0.28
2 [97]	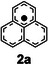	σ‐dimerization	−9.8	−11	n.d.	−1.64 [98] −0.04 [98]
3 [65]	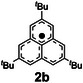	π‐dimerization	−9.5	−36	544	−1.64 −0.11
4 [68]	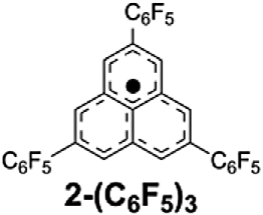	σ‐dimerization	−15	−32	n.d.	−1.30 +0.36

### 2.3 Photo‐Responsive σ‐Bond Scission and Subsequent Recombination

**FIGURE 5:**
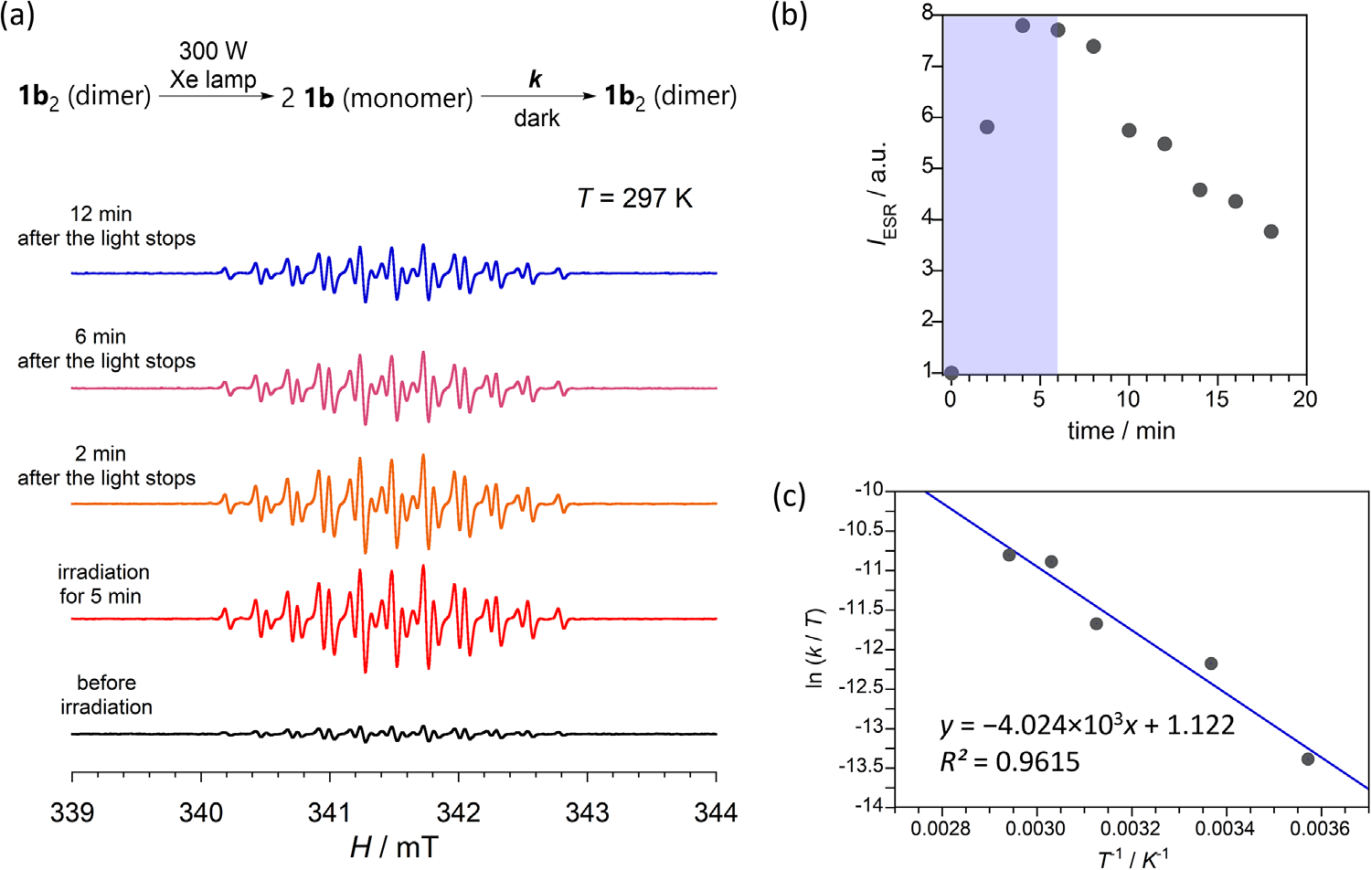
(a) Time‐dependent changes in the ESR spectra of **1b**_2_ at 297 K. (b) Profile of the ESR signal intensity at 297 K. During the purple‐colored period, a Xe lamp was turned on. (c) Eyring–Polanyi plot for the thermal recombination of **1b**.

**SCHEME 2:**
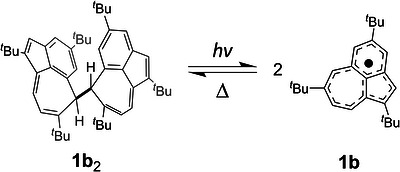
Photochromic reaction of **1b**_2_ and thermal recombination of **1b**.

The scission of the σ‐bond in **1b**_2_ can be facilitated by light irradiation. Irradiating a toluene solution of **1b**_2_ with a Xe lamp for 5 min at 297 K resulted in an eightfold increase in the intensity of the ESR signal (Figure 5a). Once irradiation was stopped, the signal intensity gradually returned to its original level (Figure 5b). This observation suggests that thermal recombination occurs, that is, that radical **1b** thermally reverts to σ‐dimer **1b**_2_ (Scheme 2) [99, 100, 101]. During the process, the decomposition of **1b**_2_ was negligible, confirmed by ^1^H NMR measurements (Figure S16).

An analysis of the decay profile and how it varies with temperature allowed us to determine the rate constant for the thermal recombination of **1b** to **1b**_2_. Using a second‐order kinetic expression, we established the following kinetic parameters: Δ*G* ^‡^ (298 K) = 21 kcal·mol^−1^, Δ*H* ^‡^ = 8.0 kcal·mol^−1^, and Δ*S* ^‡^ = −45 cal·K^−1^·mol^−1^. To the best of our knowledge, there are only few reports on the kinetic parameters associated with the dimerization processes of persistent hydrocarbon radicals [102, 103, 104]. The second‐order reaction rate constant (*k*) for the radical recombination of **1b** was determined to be 0.0015 M^−1^s^−1^ at 297 K. This value is much smaller than those of the radical recombination of lophyl radicals to give hexaarylbiimidazoles (*k* ∼ 10^2^–10^4^ M^−1^s^−1^) [105, 106], thus illustrating the high kinetic stability of **1b**. The theoretical estimate for Δ*G* ^‡^ (298 K) at the (U)B3LYP‐D3(BJ)/6‐311G(d)/SMD(toluene) level is +15.4 kcal·mol^−1^ (Figure S28), that is, smaller than the experimentally determined value. The diffusion of the generated radicals **1b** in solution may increase the observed energy barrier for thermal recombination. From the rate constants (*k*) for the thermal recombination of **1b** to **1b**_2_ and the equilibrium constants for the σ‐dimerization (*K*_dimer_), the kinetic parameters for the dissociation process of **1b**_2_ to **1b** are addressed (Table S3 and Figure S14). The Gibbs free energy of activation for the dissociation was estimated to be 31 kcal·mol^−1^ at 298 K, supporting the requirement of higher energy for the dissociation than the recombination (Figure S28).

**FIGURE 6:**
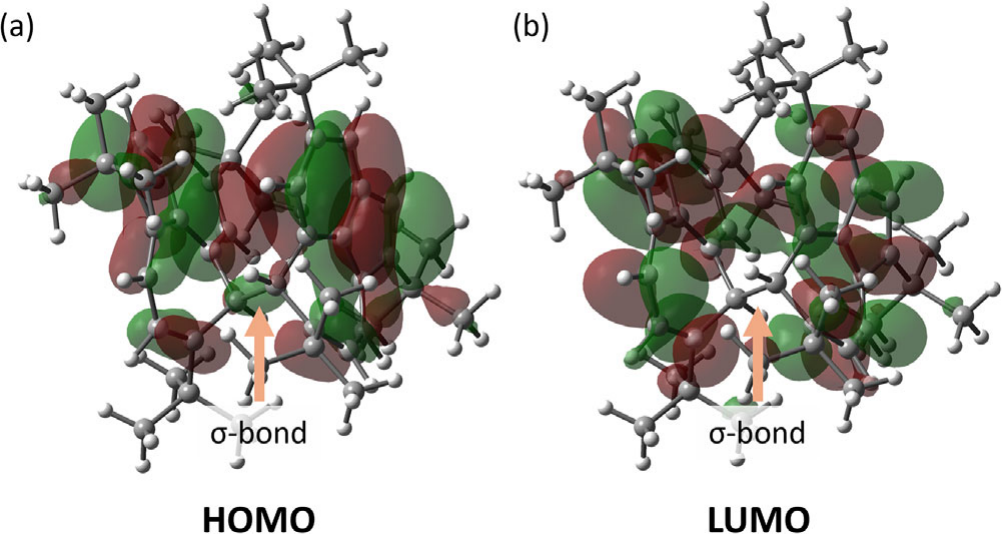
(a) HOMO and (b) LUMO for **1b**_2_ at the RB3LYP/6‐311+G(d,p)//RB3LYP‐D3(BJ)/6‐311G(d) level.

Although details of the reaction mechanism of the photo‐dissociation of **1b**_2_ remain uncertain at this stage, the σ‐bond connecting the two **1b** units in the optimized structure in the first excited singlet state (*S*_1_) of **1b**_2_, as calculated at the TD‐B3LYP‐D3(BJ)/6‐311G(d) level, is elongated (1.588 Å) compared to that of the ground state (1.581 Å as observed in the X‐ray diffraction analysis). The weakening of the σ‐bond upon photoexcitation should facilitate the homolytic dissociation of **1b**_2_. Time‐dependent (TD)‐DFT calculations indicate that the first excited state of **1b**_2_ is predominantly characterized by the HOMO–LUMO transition (Table S11). Within the σ‐bond of **1b**_2_, the HOMO displays a bonding interaction, whereas the LUMO introduces a nodal plane (Figure 6). This transition leads to a reduction of the σ‐bond order upon photoexcitation. Furthermore, the moderate reactivity of radical **1b**, which stems from the unsymmetric distribution of the unpaired electron and the steric protection of the *tert*‐butyl groups, allowed us to examine the thermal recombination of **1b**.

### 2.4 Redox Properties and Electronic Absorption

**FIGURE 7:**
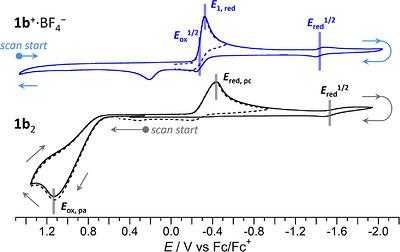
Cyclic voltammograms of **1b** ^+^·BF_4_^−^ (V vs. Fc/Fc^+^; in 0.1 M *n*Bu_4_NClO_4_/CH_3_CN; scan rate: 100 mV/s; room temperature) and **1b**_2_ (V vs. Fc/Fc^+^; in 0.1 M *n*Bu_4_NClO_4_/THF; scan rate: 100 mV/s; room temperature).

The optoelectronic measurements conducted on **1b** clearly revealed its physical properties. The σ‐dimer of **1b**_2_ exhibited an irreversible cyclic voltammogram (CV) with *E*_ox, pa_ = +1.14 V and *E*_red, pc_ = −0.44 V (vs. Fc/Fc^+^) at room temperature (Figure 7 and Table S5). The significant separation between the oxidation and re‐reduction waves indicates that dynamic structural changes occur in response to the redox stimulus [107]. The anodic peak at *E*_ox, pa_ = +1.14 V corresponds to the oxidation of **1b**_2_, which leads to the dissociation of the molecule into two monomeric cations **1b** ^+^. The cathodic peak at *E*_red, pc_ = −0.44 V is comparable to that of the reduction wave (*E*_1, red_) of the isolated cation **1b** ^+^ at −0.32 V, thus representing the reduction of **1b** ^+^ back to **1b**. The generated **1b** then immediately dimerizes to reform **1b**_2_. Together with the significant separation of the redox couple, attributed to a dynamic redox system [107], a weak reversible reduction wave, attributed to the reduction of radical **1b**, was observed at *E*_red_^1/2^ = ‒1.58 V. In the CV of **1b** ^+^ in CH_3_CN, the reduction wave was more clearly observed at *E*_red_^1/2^ = ‒1.44 V, and the oxidation wave from **1b** to **1b** ^+^ was also seen at *E*_ox_^1/2^ = ‒0.28 V. While the oxidative half‐wave potential (*E*_ox_^1/2^) of **1b** shows a negative shift compared to that of **2b** (‒0.11 V vs. Fc/Fc^+^) [59, 98, 108], the reductive wave half‐wave potential (*E*_red_^1/2^) of **1b** shows a positive shift relative to **2b** (‒1.64 V) [59, 98, 108]. Replacing the hexagons of **2b** with a pentagon and heptagon thus enhances the electron‐donating properties and electron affinity of **1b**.

**FIGURE 8:**
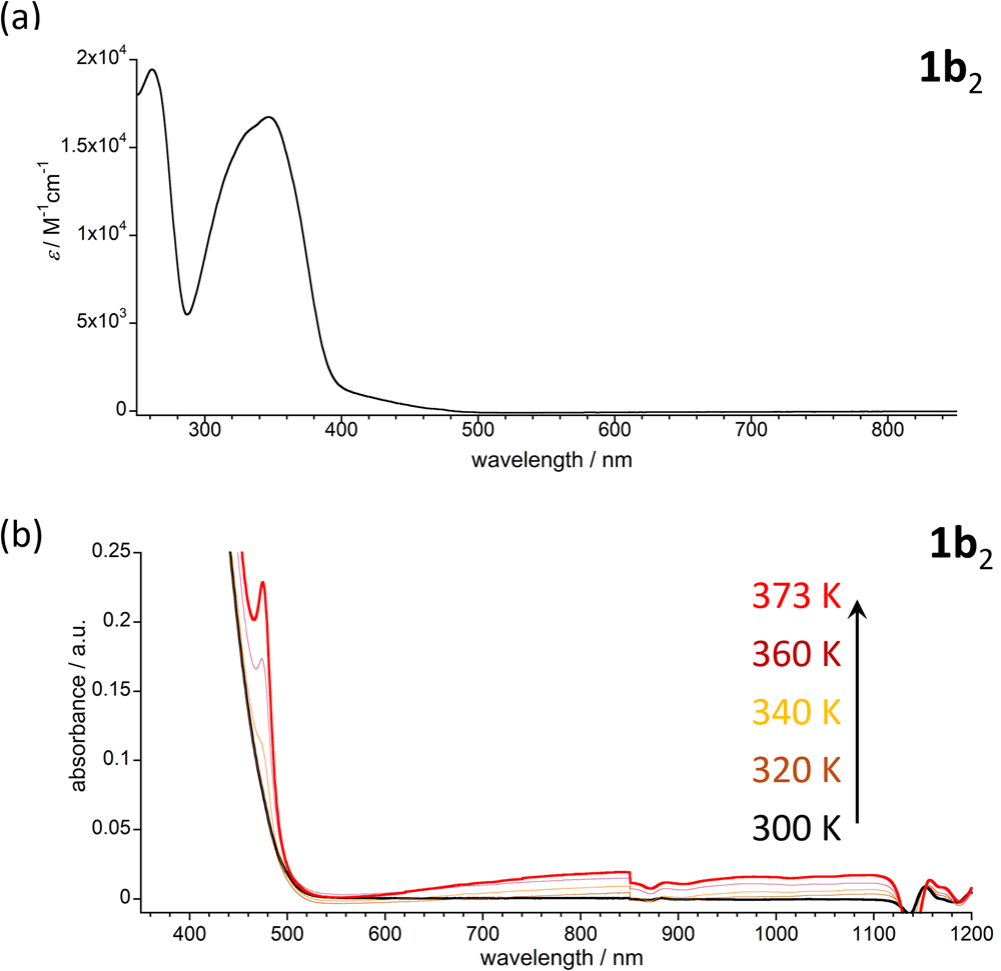
(a) Electronic absorption spectrum of **1b**_2_ in hexane at room temperature. (b) Magnified VT electronic absorption spectra of **1b**_2_ in toluene.

The nonalternant character of **1b** is also reflected in the electronic absorption spectrum. At room temperature, an intense absorption was observed at 347 nm with a tailing absorption ranging from 400 to 500 nm (Figure 8a). Based on time‐dependent density‐functional‐theory (TD‐DFT) calculations at the B3LYP/6‐311+G(d,p) level (Figure S27 and Table S11), these absorptions were identified as those of σ‐dimer **1b**_2_. However, upon heating the measured solution from 300 to 373 K, a broad band centered at around 850 nm and a sharp band at 475 nm appeared (Figure 8b). These new absorptions can be attributed to monomeric radical **1b**. The TD‐DFT calculations indicated that the longest absorption band at 850 nm primarily corresponds to the *D*_0_ → *D*_1_ transition from the *α*‐SOMO to the *α*‐LUMO of **1b** (Figure S24 and Table S8). Notably, the *D*_0_ → *D*_1_ transition energy of **1b** is red‐shifted compared to that of the monomeric form of phenalenyl **2** (*λ* ∼ 550 nm) [59, 60]. The same absorptions observed under heating conditions were also detected under photoirradiation with a Xe lamp, thus providing more evidence to support the photochromic behavior demonstrated by the ESR measurements (Figure S21). Replacing the benzenoid rings in **2** with the nonalternant structure thus effectively reduces the photoexcitation energy of **1**.

## 3 Conclusion

In conclusion, we have successfully synthesized and characterized tri‐*tert*‐butyl‐substituted benzo[*cd*]azulenyl **1b**, which is a nonalternant isomer of **2b** that had previously been unknown. Despite possessing kinetic protection, **1b** predominantly exists in a σ‐dimeric form, referred to as **1b**_2_, which was isolated in crystalline form. Due to the relatively weak σ‐bond in **1b**_2_, it exhibits thermal‐ and photoresponsive properties that facilitate the formation of monomeric radical **1b** through bond dissociation, which allowed us to determine both the thermodynamic and kinetic parameters for the bond‐dissociation/‐recombination process.

In the case of benzo[*cd*]azulenyl radical **1**, the unpaired electron is delocalized over almost the entire molecular structure, similar to phenalenyl radical **2**. However, unlike **2**, which has *D*_3*h*_ symmetry and a highly symmetric spin distribution, the spin density of **1** is skewed toward its five‐ and seven‐membered rings. This unsymmetrical spin density, combined with the kinetic protection offered by the three *tert*‐butyl groups, gives **1b** regio‐ and stereoselective C─C‐bond‐formation properties with a noticeable reaction rate. The nonalternant nature of **1b**, resulting from the replacement of the two hexagons of **2b** with a pentagon and a heptagon, is clearly reflected in its optoelectronic properties, where the electron‐donating properties and electron affinity are enhanced and the photoexcitation energy is reduced compared to **2b**.

Our study has shed light on the chemical and physical effects that the introduction of nonhexagonal rings in a nonalternant hydrocarbon structure exerts on the unpaired electron. This type of study has rarely been conducted within the context of nonalternant isomers of doublet open‐shell graphene fragments. Further studies on the benzo[*cd*]azulenyl‐based scaffold, including the isolation of the monomeric radical and the exploration of other dimerization modes, are currently in progress in our group.

## 4 Experimental Section

All synthetic procedures and characterization data for unknown compounds are provided in the Supporting Information. The data that supports the findings of this study are available in the Supplementary Information associated with this article.

## Conflicts of Interest

The authors declare no conflicts of interest.

## Supporting information



The authors have cited additional references within the Supporting Information [92, 93, 109, 110, 111, 112]. Deposition numbers 2520797 (for **1b** ^+^·BF_4_^−^), and 2520798 (for **1b**_2_) contain the supplementary crystallographic data for this paper. These data are provided free of charge by the joint Cambridge Crystallographic Data Centre and Fachinformationszentrum Karlsruhe Access Structures service.

**Supporting File 1**: chem70947‐sup‐0002‐Data.zip.
