# Supplementary material for: Benzo[cd]azulenyl: A Structural Isomer of Phenalenyl—Synthesis and Properties of Its Tri‐tert‐butyl‐substituted Derivative and Formation of a Thermal‐ and Photoresponsive σ‐Dimer
Source: Chemistry. 2026 Mar 26;32(22):e70947. doi: 10.1002/chem.70947 (PMC13250364; doi:10.1002/chem.70947)
Supplement: Supplementary file 1 — The authors have cited additional references within the Supporting Information [92, 93, 109, 110, 111, 112]. Deposition numbers 2520797 (for 1b +·BF4 −), and 2520798 (for 1b 2) contain the supplementary crystallographic data for this paper. These data are provided free of charge by the joint Cambridge Crystallographic Data Centre and Fachinformationszentrum Karlsruhe Access Structures service. [file CHEM-32-e70947-s002.pdf]

Supporting Information

Benzo[*cd*]azulenyl, a Structural Isomer of Phenalenyl: Synthesis and Properties of its Tri-*tert*-butyl-substituted Derivative and Formation of a Thermal- and Photoresponsive σ-Dimer

Kaho Takeuchi,[a] Akihito Konishi,*[a,b] and Makoto Yasuda*[a,b]

[a] Department of Applied Chemistry, Graduate School of Engineering, The University of Osaka, 2-1 Yamadaoka, Suita, Osaka 565-0871, Japan

[b] Innovative Catalysis Science Division, Institute for Open and Transdisciplinary Research Initiatives (ICS-OTRI), The University of Osaka, Suita, Osaka 565-0871, Japan

Table of contents

1. General 3

2. Materials 3

3. Synthetic procedures 4

4. Stability of 1b and $1b_2$ 24

5. X-ray crystallographic data 25

5-1. Summary for crystallographic data of $1b^{+}\cdot BF_4^{-}$ 25

5-2. Summary for crystallographic data of $1b_2$ 26

6. Summary of the observed geometries 27

7. ESR measurements 28

7-1. ESR spectrum of 1b 28

7-2. VT-ESR measurement of 1b 29

7-3. Evaluation of photo-stimulus dissociation of the σ-bond in $1b_2$ 32

8. Electrochemical properties 38

9. Electronic absorption spectra 39

10. Computational method 41

10-1. General 41

10-2. Molecular orbitals 42

10-3. TD-DFT calculations 45

10-4. NICS(1) and ACID calculations 51

10-5. Radical stabilization energies (RSEs) 52

10-6. Cartesian coordinates for the optimized geometries 53

11. Dimerization from 1b to $1b_2$ 59

11-1. General 59

11-2. Summary of each reaction step 60

11-3. Cartesian coordinates for the optimized geometry for each reaction step 61

11-3-1. Cartesian coordinates for the optimized geometry for IM1 61

11-3-2. Cartesian coordinates for the optimized geometry for TS1 62

11-4. Relative energy of other possible σ-dimers to $1b_2$ 63

12. References 68

1. General

NMR spectra were recorded on JEOL-AL400, JEOL-ECS400 (400 MHz for ^{1}H, 100 MHz for ^{13}C, and 372 MHz for ^{19}F NMR) and Bruker AVANCE III spectrometers (600 MHz for ^{1}H, and 150 MHz for ^{13}C NMR). ^{1}H and ^{13}C NMR signals of compounds were assigned using HMQC, HSQC, HMBC, COSY, and ^{13}C off-resonance techniques. Chemical shifts were reported in ppm on the δ scale relative to tetramethylsilane (δ = 0.00 ppm for ^{1}H NMR) and $CDCl_3$ (δ = 77.16 ppm for ^{13}C NMR) as an internal reference. For ^{19}F NMR spectra, $BF_3{\cdot}OEt_2$ in $CDCl_3$ was employed as an external standard (–153.0 ppm). ESR spectra were recorded on a Bruker EMXmicro spectrometer equipped with a Bruker ER4119HS-W1 a high-sensitivity cavity resonator, a variable temperature control unit including a liquid N_2 cryostat, and a temperature controller. The magnetic field was calibrated at room temperature with a Bruker strong pitch standard ($g = 2.0028$). ESR spectral simulation was performed by using the software of EasySpin (Version 6.0.6).[1] Positive FAB, EI, MALDI-TOF mass spectra were recorded on a JEOL JMS-700, a Shimadzu GCMS-QP2010 Ultra, and a JEOL JMS-S3000, respectively. The high-resolution DART or ESI mass spectra were analyzed by using a JEOL JMS-T100LP. IR spectra were recorded as thin films or as solids in KBr pellets on a JASCO FT/IR 6200 spectrophotometer, or as solids in ATR-mode on a JASCO FT/IR-4X spectrophotometer. UV-vis-NIR spectra were recorded on a JASCO V-770 spectrophotometer. Fluorescence spectra were recorded using a JASCO FP-8550 spectrophotometer. For variable temperature measurements, a UNISOKU CoolSpeK cryostat was employed. Cyclic voltammetric measurements were performed with an ALS-600C electrochemical analyzer using a glassy carbon working electrode, a Pt counter electrode, and an Ag/$AgNO_3$ reference electrode at room temperature in CH_3CN or THF containing 0.1 M $^n Bu_4NClO_4$ as the supporting electrolyte. Data collection for X-ray crystal analysis was performed on Rigaku/XtaLAB Synergy-S/Cu (CuK_α λ = 1.54187 Å) diffractometers. All non-hydrogen atoms were refined with anisotropic displacement parameters and hydrogen atoms were placed at calculated positions and refined "riding" on their corresponding carbon atoms by Olex2[2] program.

2. Materials

Anhydrous dichloromethane, THF, acetonitrile, diethyl ether, toluene and hexane were purchased and used as obtained. All reagents were obtained from commercial suppliers and used as received. Compounds **3**[3] and **4**[4] were synthesized according to the reported literatures. The reaction for the generation **1b** from **1b**$^+{\cdot}BF_4^-$ was performed in a nitrogen-filled glovebox.

3. Synthetic procedures

1,6-Di-*tert*-butylazulene 4[4]

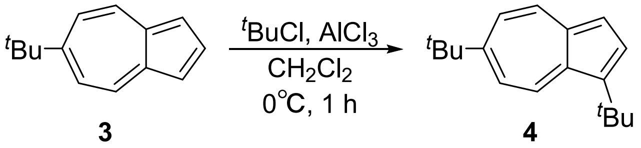

Aluminium chloride ($AlCl_3$, 0.763 g, 5.54 mmol) was added to a solution of **3**[3] (0.929 g, 5.04 mmol) and *tert*-butyl chloride (0.62 mL, 5.54 mmol) in dichloromethane (30 mL). After stirring at 0 °C for 1.5 h, the reaction mixture was poured onto ice. The organic layer was washed with water and dried over Na_2SO_4. The filtrate was evaporated, and the residue was purified by column chromatography on silica gel with hexane to give **4** as a blue solid (0.783 g, 3.26 mmol, 65%).

^{1}H NMR (400 MHz, $CDCl_3$) 8.62 (d, J = 10.4 Hz, 1H), 8.21 (d, J = 10.4 Hz, 1H), 7.75 (d, J = 4.0 Hz, 1H), 7.26 (dd, J = 10.8, 1.6 Hz, 1H), 7.23 (dd, J = 10.6, 1.8 Hz, 1H), 7.20 (d, J = 4.0 Hz, 1H), 1.59 (s, 9H), 1.45 (s, 9H); ^{13}C{^{1}H} NMR (100 MHz, $CDCl_3$) 160.8, 140.3, 139.2, 135.7, 134.9, 134.7, 133.4, 120.2, 119.0, 115.8, 38.5, 33.5, 32.3, 32.0; HRMS (DART$^+$) Calculated: ($C_{18}H_{25}$) 241.1951 ([M + H]$^+$); Found: 241.1957.

^{1}H NMR (400 MHz, $CDCl_3$)

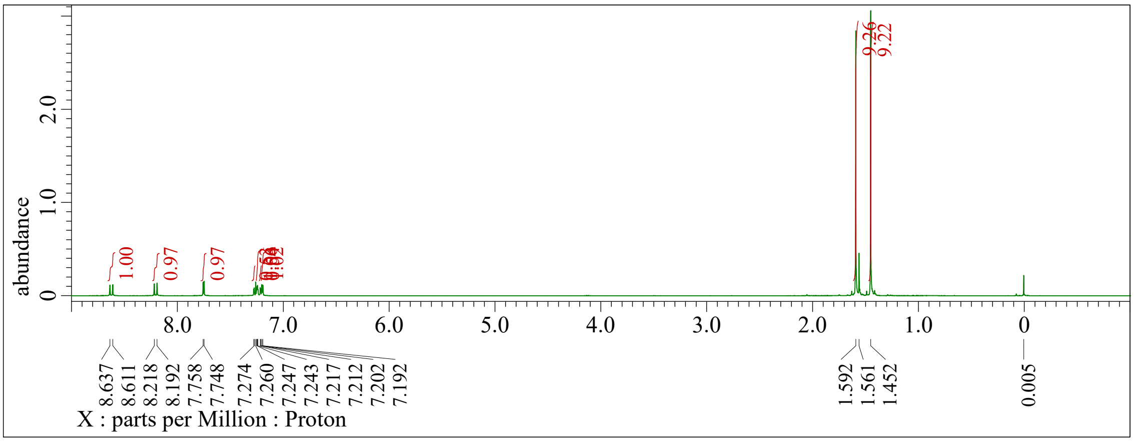

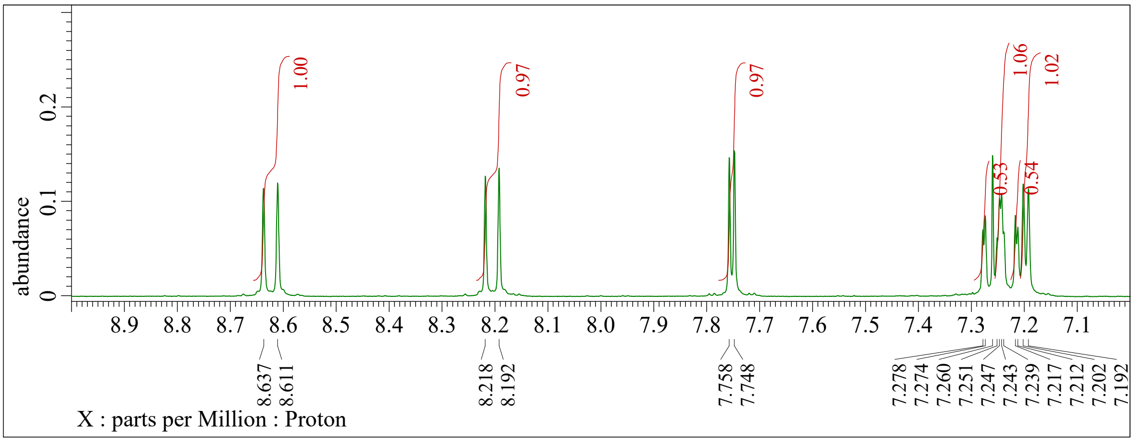

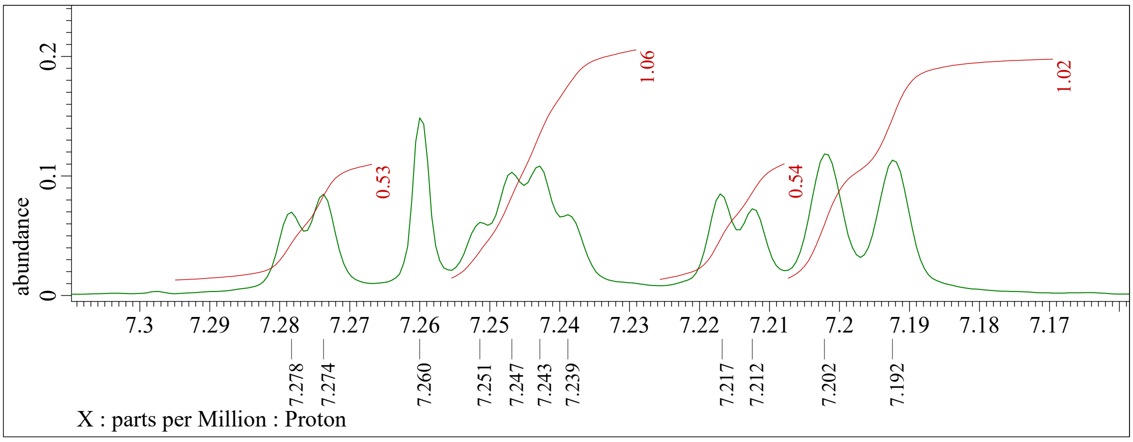

$^{13}C\{^{1}H\}$ NMR (100 MHz, $CDCl_3$)

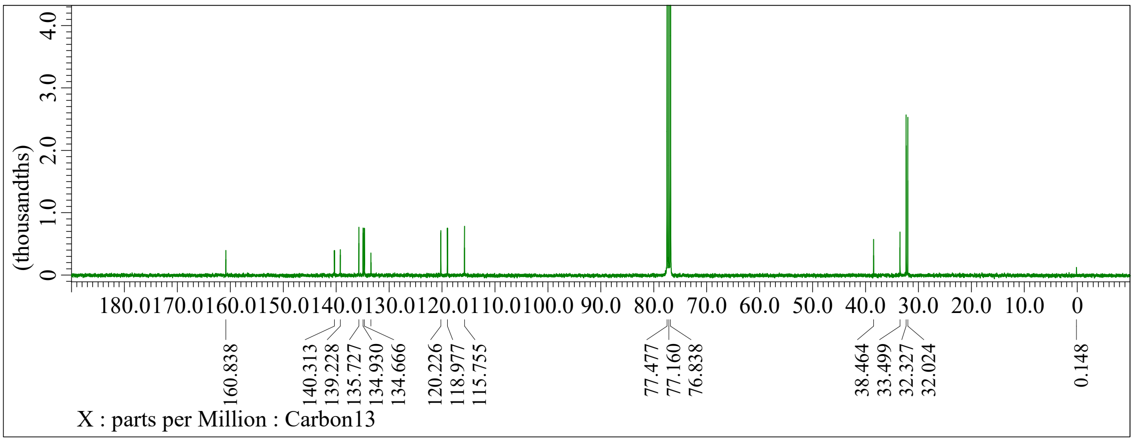

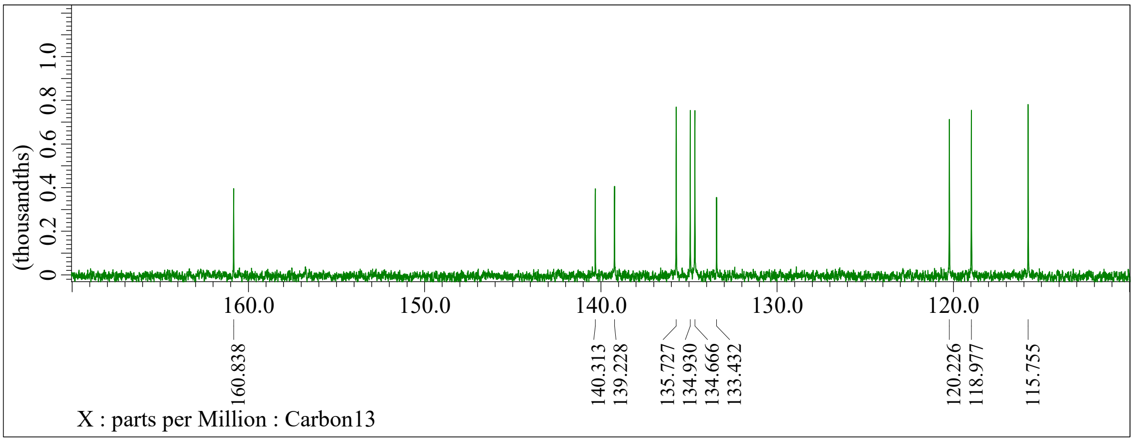

1,6-Di-*tert*-butyl-4-methylazulene 5

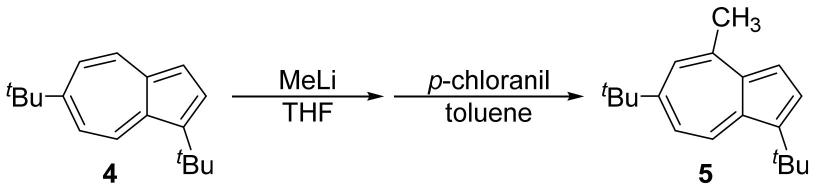

A solution of MeLi (6.2 mL, 19.2 mmol, 3.1 M in diethoxymethane) was added to a solution of **4** (1.84 g, 7.65 mmol) in THF. The mixture was stirred at 50 °C for 3 h. After 1 M HCl aq. was added to the reaction mixture, the organic phase was extracted with ether and washed with water. The extracts were dried over Na_2SO_4 and the filtrate was concentrated in vacuum. The brown residue was dissolved in toluene (20 mL), then *p*-chloranil (2.82 g, 11.5 mmol) was added. The mixture was stirred at 50 °C for 3 h. After the reaction, the solvents were removed in vacuum. The residues were purified by column chromatography on silica gel with hexane to give **5** as blue sticky gum (1.52 g, 5.97 mmol, 78%).

IR (ATR) ν = 3085 (w), 2963 (s), 2904 (m), 2870 (m), 1695 (w), 1578 (m), 1366 (m), 1261 (m), 1033 (m), 809 (m) cm^{-1}; ^1H NMR (400 MHz, acetone-d_6) 8.70 (d, J = 10.4 Hz, 1H), 7.62 (d, J = 4.0 Hz, 1H), 7.35 (s, 1H), 7.34 (d, J = 11.6 Hz, 1H), 7.19 (d, J = 3.6 Hz), 2.86 (s, 3H), 1.57 (s, 9H), 1.46 (s, 9H); $^{13}C\{^1H\}$ NMR (100 MHz, acetone-d_6) 159.9, 145.2, 139.6, 138.4, 135.5, 134.9, 133.5, 124.7, 118.6, 114.1, 38.9, 33.7, 32.6, 32.2, 25.6; HRMS (DART$^+$) Calculated: ($C_{19}H_{27}$) 255.2107 ($[M + H]^+$), Found: 255.2115.

^1H NMR (400 MHz, acetone-d_6)

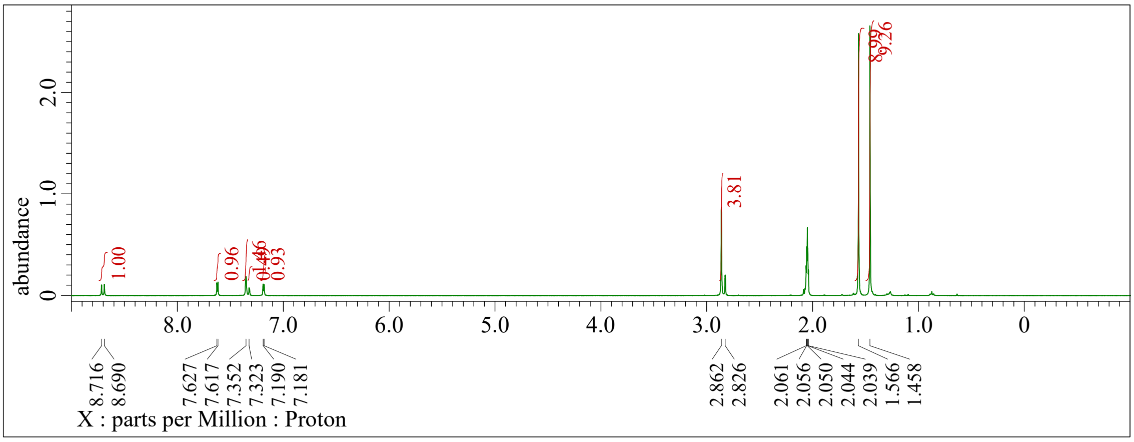

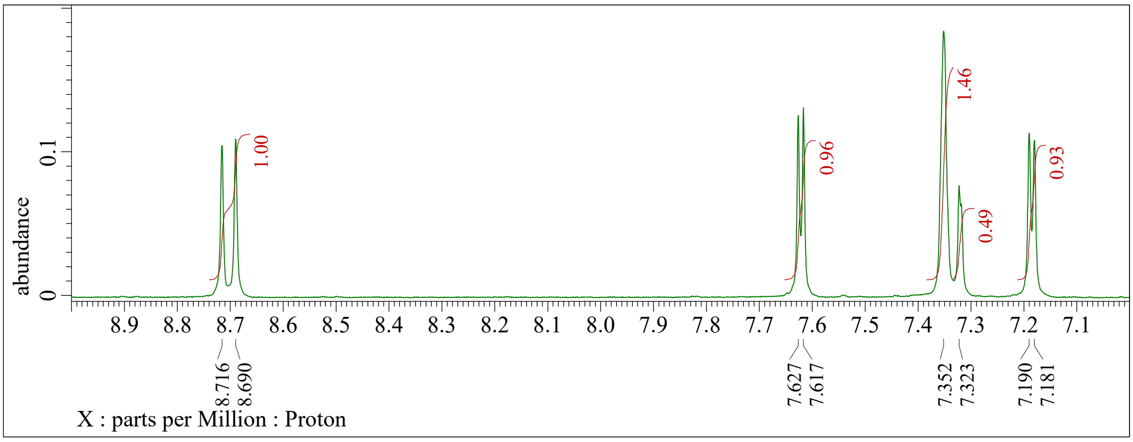

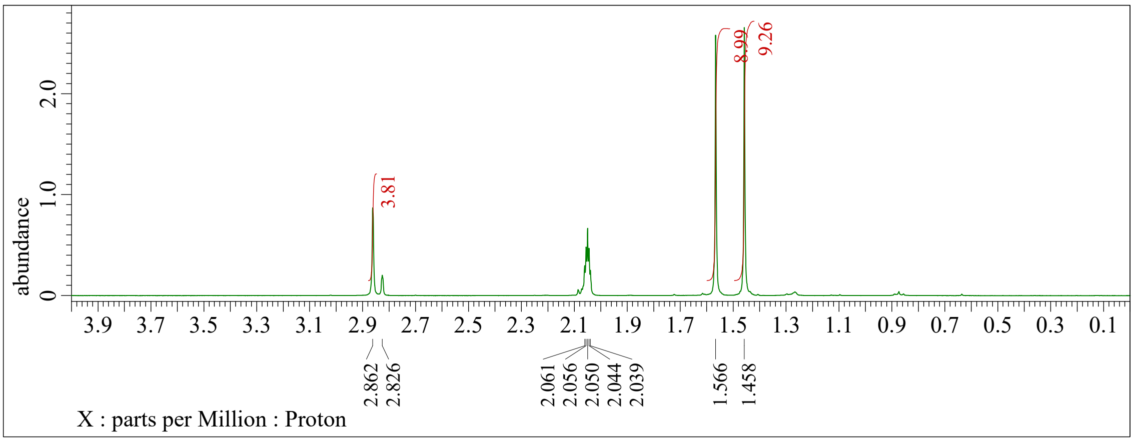

$^{13}C\{^{1}H\}$ NMR (100 MHz, acetone-d_6)

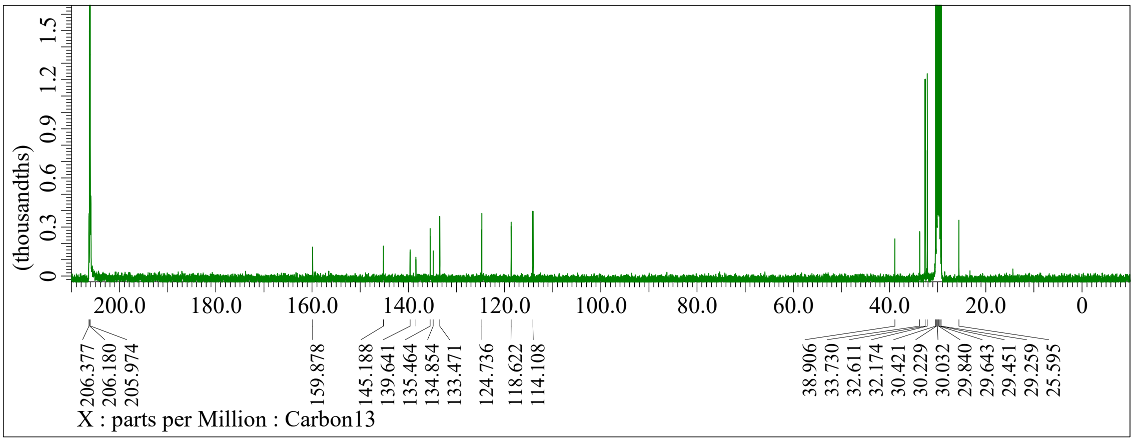

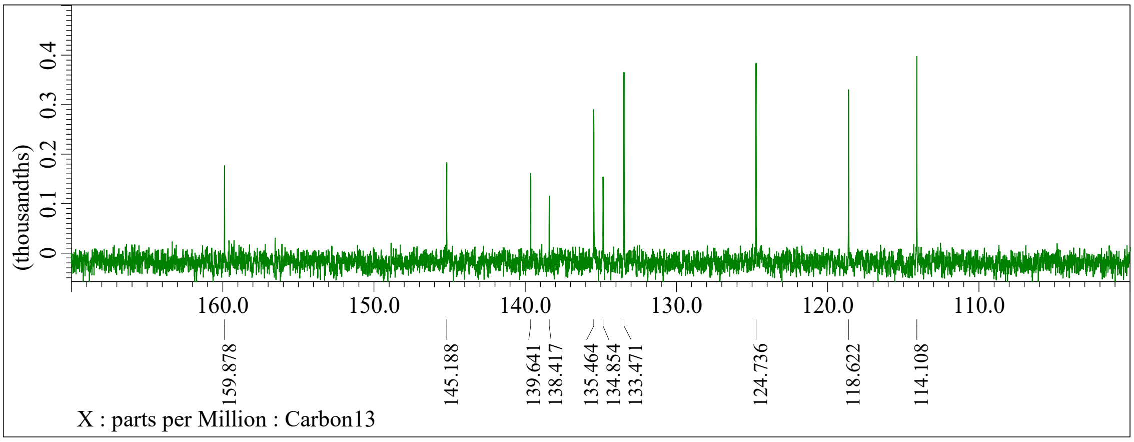

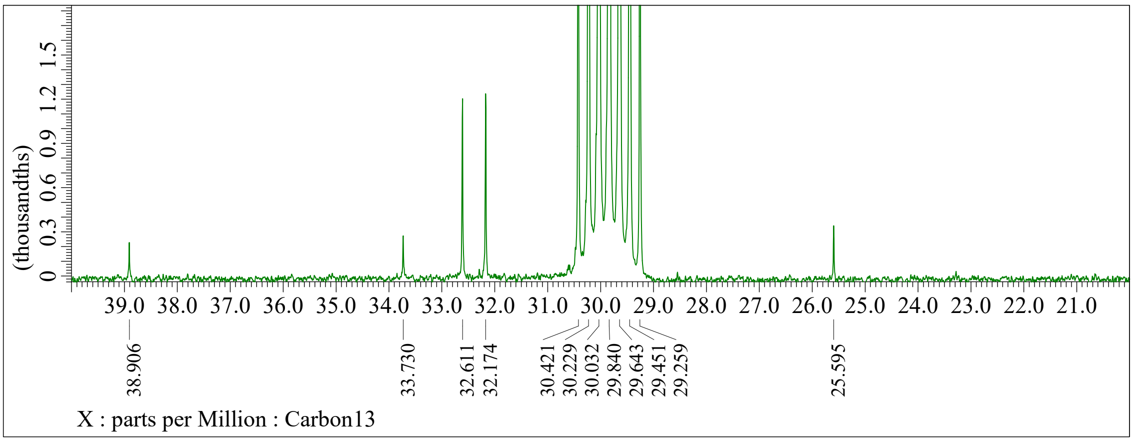

2-{(1,6-Di-*tert*-butylazulen-4-yl)methyl}-2-hydroxy-3,3-dimethylbutanoic acid 6

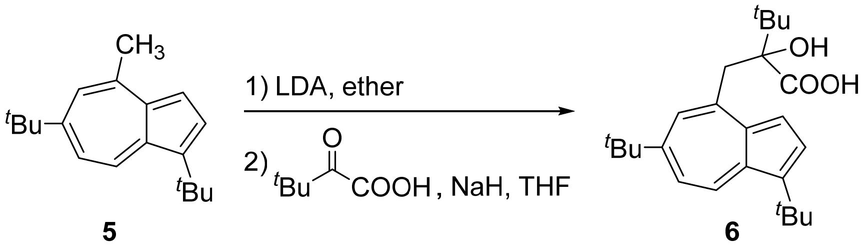

Under a nitrogen atmosphere, to a solution of **5** (0.609 g, 2.39 mmol) in ether (20 mL) was added LDA (0.656 M, 4.4 mL in THF, prepared from $^{i}Pr_2NH$ (1 mL, 7.19 mmol) and ^{n}BuLi (4.4 mL, 6.83 mmol, 1.55 M in hexane) at 0 °C and the reaction mixture was stirred for 15 min. In another flask, a solution of 3,3-dimethyl-2-oxobutanoic acid (0.46 mL, 3.61 mmol) in THF (5 mL) was treated with NaH (0.144 g, 3.61 mmol, 60% in oil) at 0 °C. After the deprotonation of **5** with LDA, a suspension of carboxylate in THF was added to the solution at 0 °C and additional THF (6~8 mL) was used for washing the suspension to the reaction mixture. The reaction mixture was stirred overnight at room temperature. After 1 M HCl aq. was added to the reaction mixture, the organic phase was extracted with ether. The collected ether phase was washed with NaOH aq. The collected NaOH aq. was acidified with 3 M HCl aq. and re-extracted with ether. The organic phase was washed with water and dried over Na_2SO_4. The filtrate was evaporated to give **6** as a blue solid (0.505 g, 1.31 mmol, 55%).

mp 179.0–179.5°C; IR (ATR) ν = 3223 (w), 3088 (w), 2955 (s), 2871 (m), 1697 (m), 1579 (m), 1365 (m), 1230 (w), 1105 (m), 793 (m) cm^{-1}; ^{1}H NMR (400 MHz, $CDCl_3$) 8.69 (d, J = 10.4 Hz, 1H), 7.73 (d, J = 3.6 Hz, 1H), 7.35 (d, J = 1.6 Hz, 1H), 7.31 (dd, J = 10.8, 2.0 Hz, 1H), 7.16 (d, J = 3.2 Hz, 1H), 3.86 (d, J = 14.0 Hz, 1H), 3.75 (d, J = 13.6 Hz, 1H), 3.18 (s, 1H), 1.58 (s, 9H), 1.44 (s, 9H), 1.24 (s, 9H); $^{13}C\{^{1}H\}$ NMR (100 MHz, $CDCl_3$) 174.4, 161.2, 141.5, 141.2, 139.8, 135.6, 134.4, 134.2, 125.2, 119.3, 111.5, 84.7, 40.9, 38.9 (Two signals were overlapped.), 33.5, 32.3, 31.8, 25.4; HRMS (ESI^+) Calculated: ($C_{25}H_{37}O_3$) 385.2737 ($[M + H]^+$), Found: 385.2727.

^1H NMR (400 MHz, $CDCl_3$)

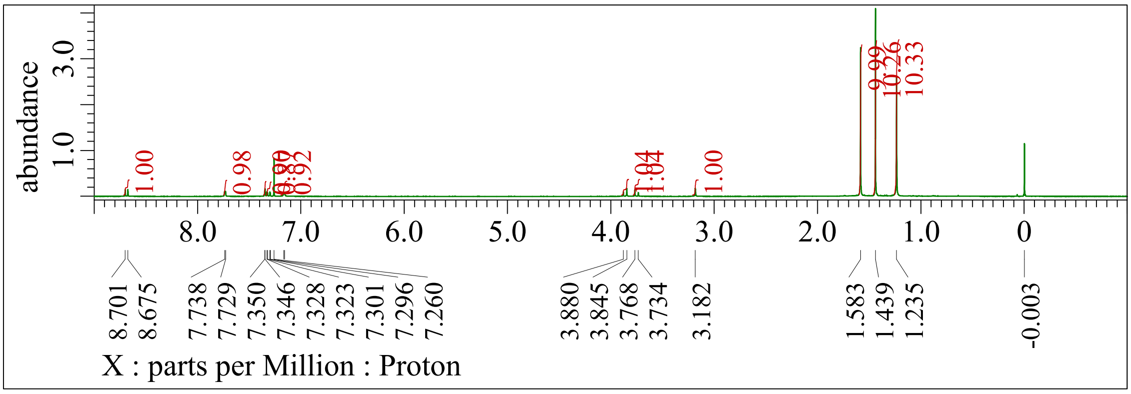

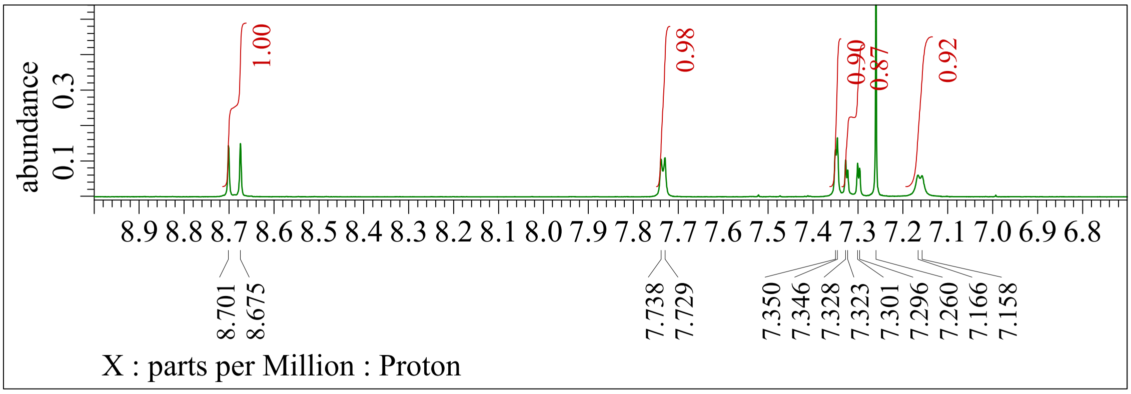

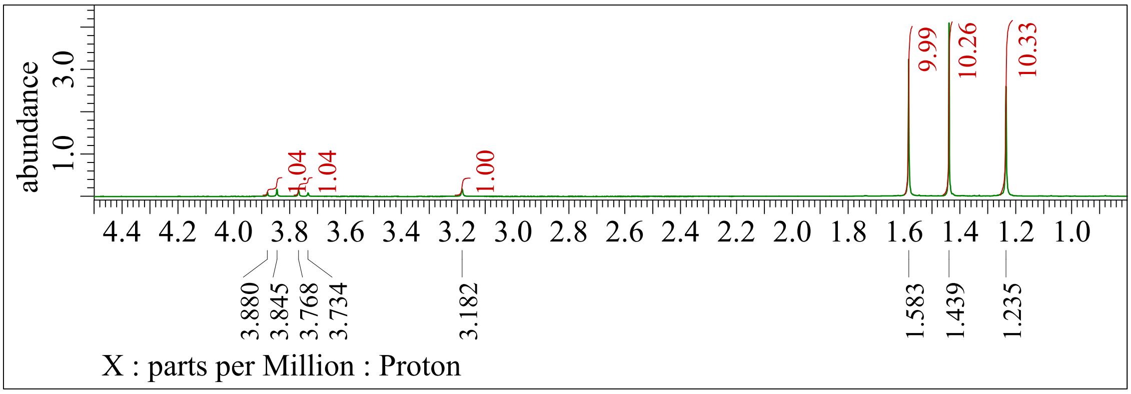

$^{13}C\{^1H\}$ NMR (100 MHz, $CDCl_3$)

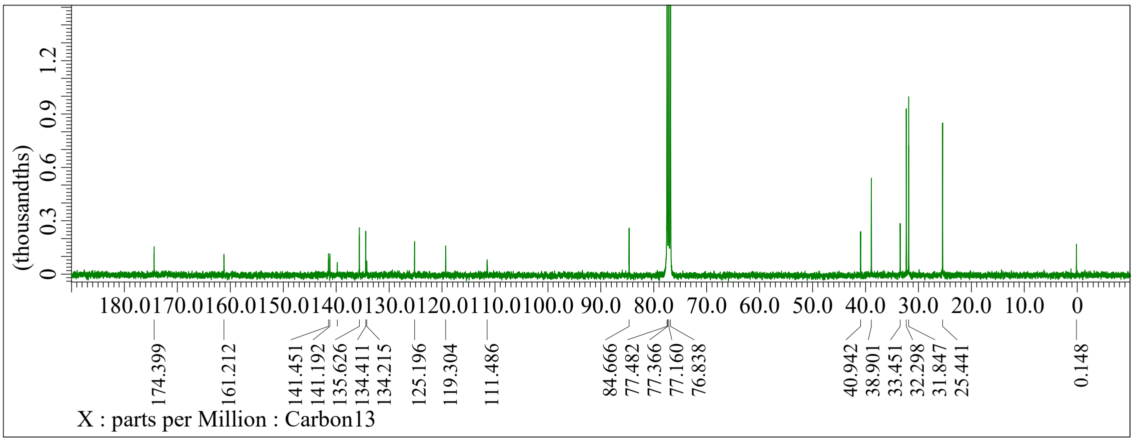

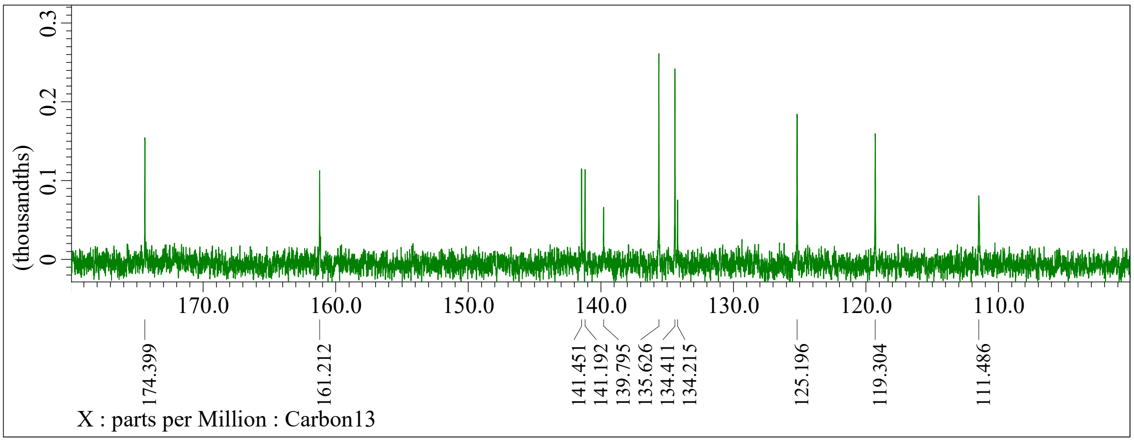

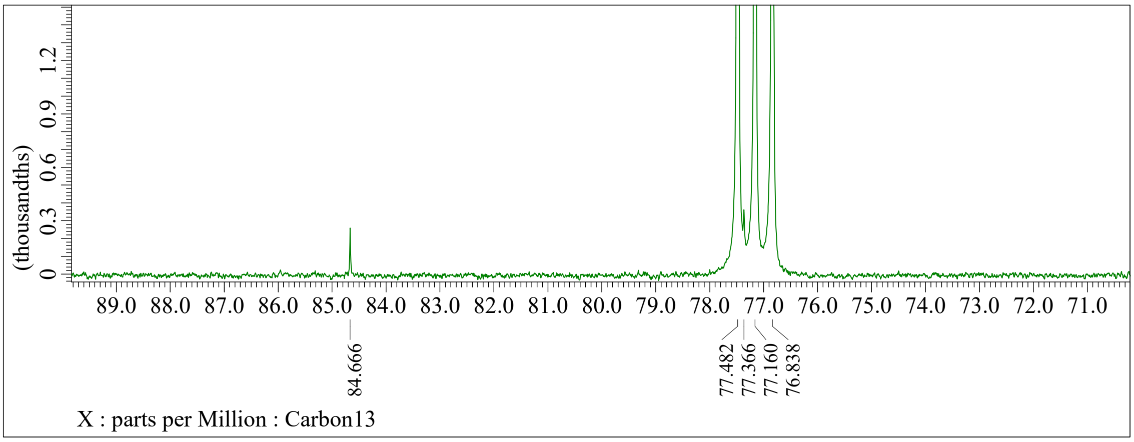

1,4,7-Tri-*tert*-butyl-4-hydroxy-4,5-dihydro-3*H*-benzo[*cd*]azulen-3-one 7

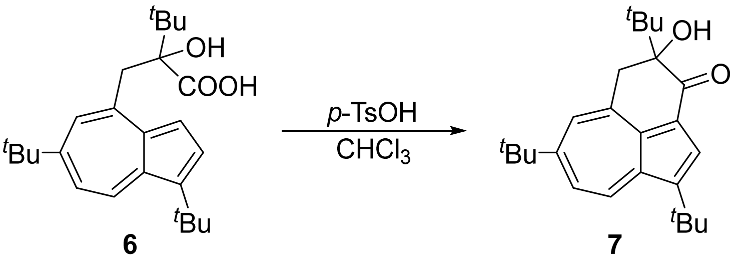

6 **7**

To a solution of **6** (0.464 g, 1.21 mmol) in $CHCl_3$ (20 mL) was added *p*-TsOH (0.138g, 0.725 mmol). The mixture was stirred at 60 °C overnight. After the solvents were removed in vacuum, the obtained solids were dissolved in ether. The ether phase was washed with 1 M NaOH aq. and dried over Na_2SO_4. The filtrate was concentrated in vacuum, and the residues were purified by column chromatography on silica gel (hexane/ethyl acetate 90:10) to give **7** (0.345 g, 0.941 mmol, 78%) as a violet solid.

mp 194.5–195.0 °C; IR (ATR) ν = 3265 (w), 3160 (w), 2959 (s), 2871 (m), 1636 (s), 1577 (m), 1460 (m), 1228 (m), 1015 (w), 681 (w) cm^{-1}; ^{1}H NMR (400 MHz, $CDCl_3$) 8.70 (d, J = 10.4 Hz, 1H), 8.02 (s, 1H), 7.61 (s, 1H), 7.58 (dd, J = 10.8 Hz, 1.6 Hz), 3.89 (d, J = 17.6 Hz, 1H), 3.66 (d, J = 17.2 Hz, 1H), 1.57 (s, 9H), 1.50 (s, 9H), 0.84 (s, 9H); $^{13}C\{^{1}H\}$ NMR (100 MHz, $CDCl_3$) 194.4, 162.9, 147.4, 143.1, 143.0, 137.2, 137.0, 130.2, 126.4, 124.0, 121.4, 79.9,

45.5, 39.9, 39.1, 33.4, 32.1, 31.9, 26.0; HRMS (MALDI-TOF-MS) Calculated: ($C_{25}H_{34}O_2Na$) 389.2451 ([M + Na]$^+$), Found: 389.2459.

^{1}H NMR (400 MHz, $CDCl_3$)

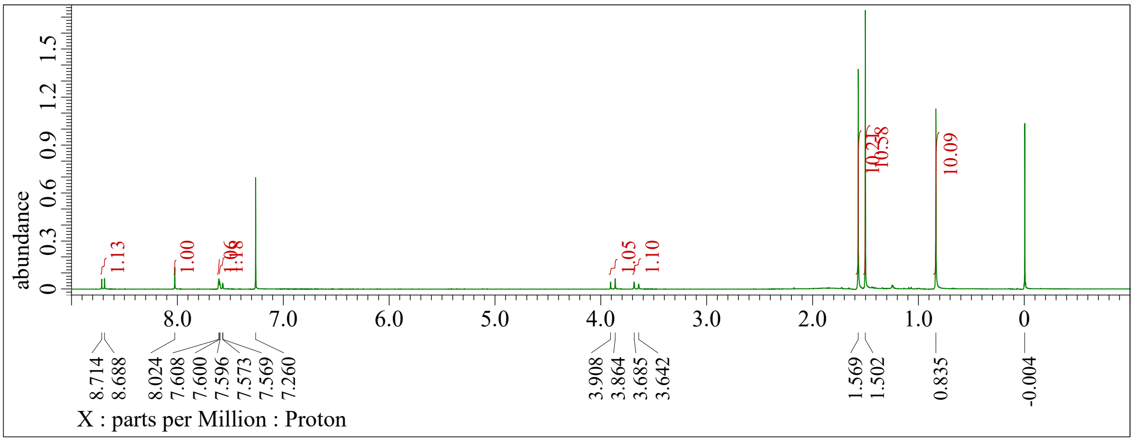

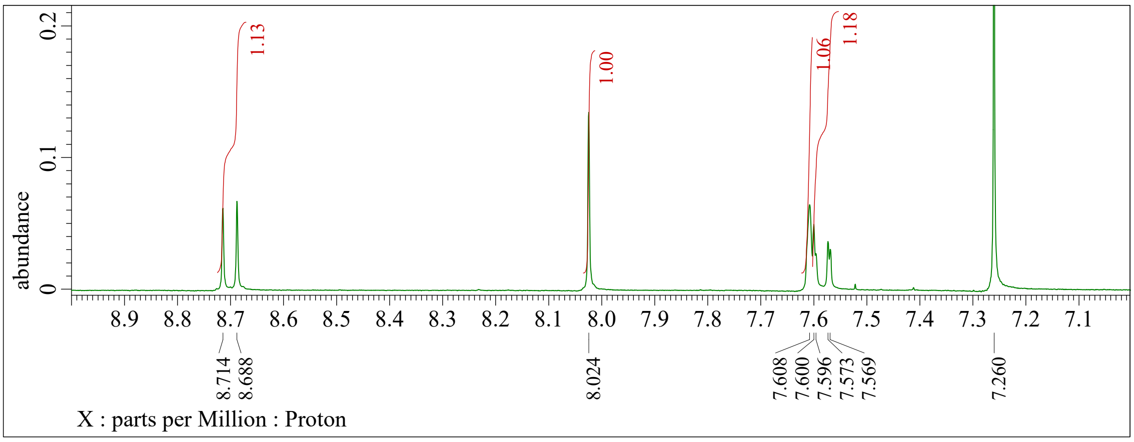

^{13}C{^{1}H} NMR (100 MHz, $CDCl_3$)

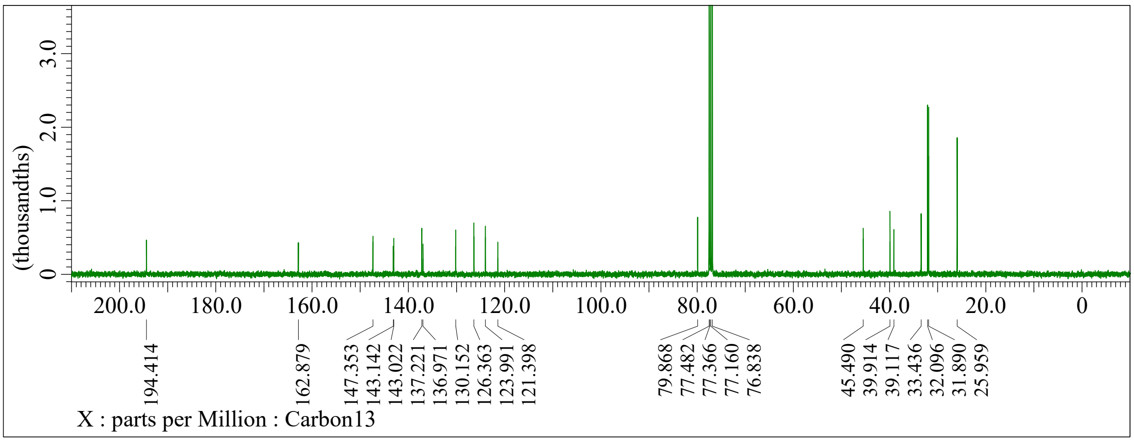

1,4,7-Tri-*tert*-butyl-4,5-dihydro-3*H*-benzo[*cd*]azulene-3,4-diol 8

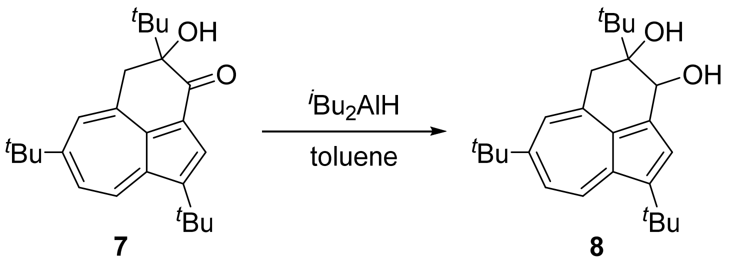

To a solution of **7** (0.122 g, 0.333 mmol) in toluene (12 mL) was added $^{i}Bu_2AlH$ (1.4mL, 1.40 mmol, 1.0 M in toluene) at −78 °C. The reaction mixture was allowed to be warmed to room temperature and stirred overnight. After 1 M HCl aq. was added to the reaction mixture, the organic phase was extracted with ether and washed with water. The extracts were dried over Na_2SO_4 and the filtrate was concentrated in vacuum. The residues were purified by column chromatography on silica gel (hexane/ethyl acetate 95:5) to give **8** (55.5 mg, 0.151 mmol, 45%) as a blue solid of a single diastereomer.

mp 191.0–191.8 °C (decomp.); IR (ATR) v = 3542 (m), 3443 (m), 3059 (w), 2952 (s), 2906 (m), 1731 (w), 1578 (m), 1460 (m), 1295 (w), 1107 (w), 972 (m) cm^{-1}; ^{1}H NMR (400 MHz, $CDCl_3$) 8.61 (d, J = 10.8 Hz, 1H), 7.70 (s, 1H), 7.28 (dd, J = 10.4, 1.2 Hz, 1H) 7.23 (s, 1H), 5.09 (d, J = 4.4 Hz, 1H), 3.72 (d, J = 16.8 Hz, 1H), 3.13 (d, J = 16.8 Hz, 1H), 1.58 (s, 9H), 1.57 (s, 9H), 1.47 (s, 1H), 1.28 (s, 9H); ^{13}C{^{1}H} NMR (100 MHz, $CDCl_3$) 160.4, 144.2, 139.8, 135.4, 133.3, 132.3, 132.0, 124.7, 124.1, 119.0, 70.1, 38.8, 37.9, 37.2, 33.5, 32.4, 32.1, 31.1, 26.6; HRMS (MALDI-TOF-MS) Calculated: ($C_{25}H_{36}O_2$) 368.2710 ([M]), Found: 368.2695.

^{1}H NMR (400 MHz, $CDCl_3$)

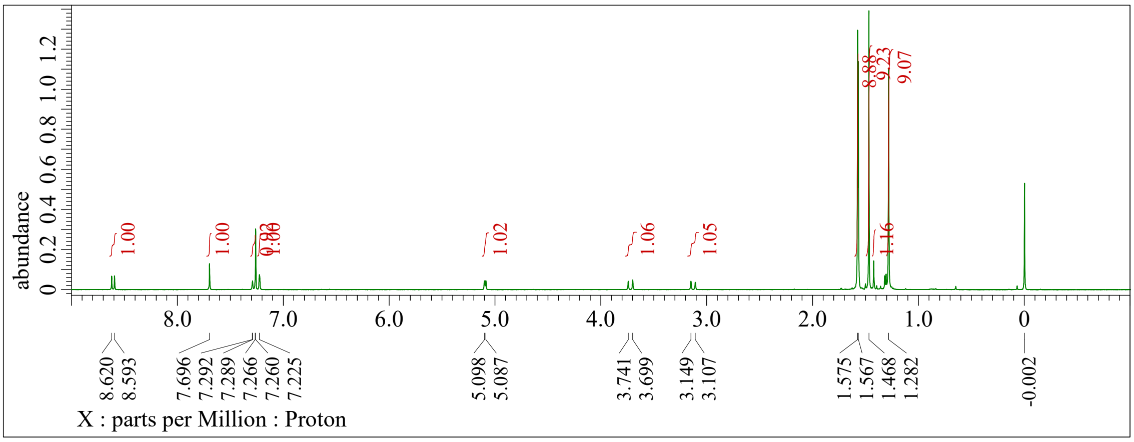

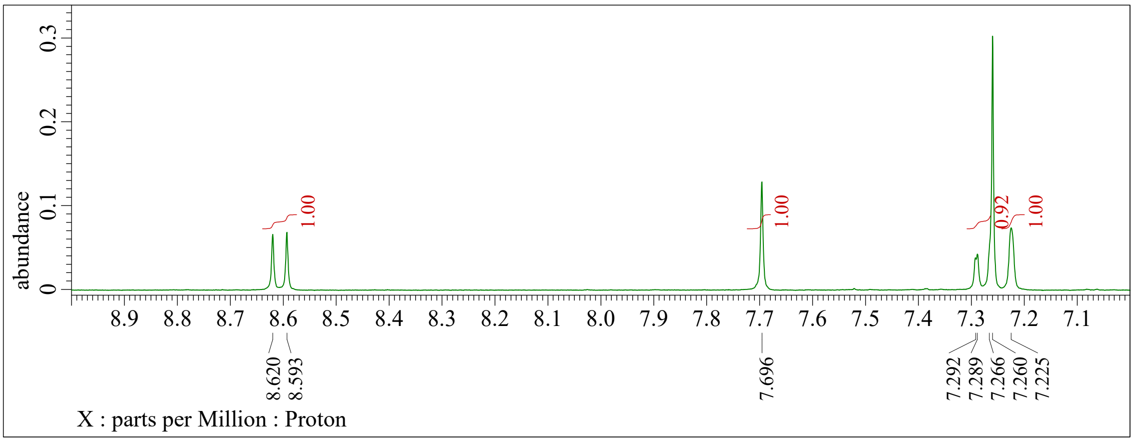

$^{13}C\{^{1}H\}$ NMR (100 MHz, $CDCl_3$)

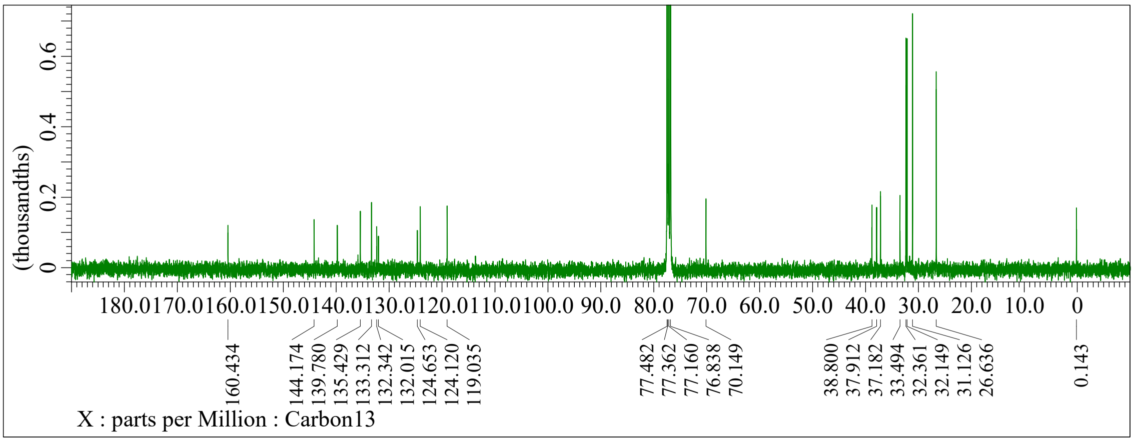

1,4,7-tri-*tert*-butyl-7*H*-benzo[*cd*]azulen-7-ylium tetrafluoroborate $1b^{+}\cdot BF_4^{-}$

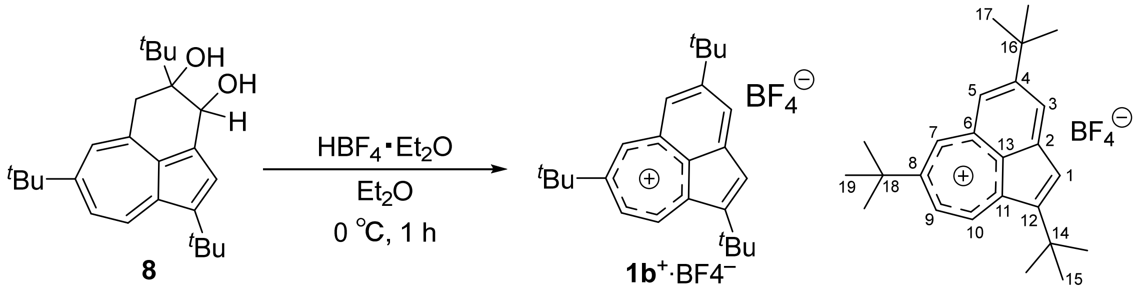

Tetrafluoroboric acid/diethyl ether complex (50–55% in diethyl ether, 0.05 mL) was added to a solution of **8** (49.8 mg, 0.135 mmol) in diethyl ether (3.5 mL). The reaction mixture was stirred at 0 ºC for 1 h. The layer of hexane (15 mL) was placed on the reaction mixture. Slow stirring the mixture led to the formation of a green precipitate. The precipitate was collected by filtration washed with diethyl ether and dried in a vacuum to give $\mathbf{1b}^{+}\cdot BF_4^{-}$ as a green solid. The recrystallization of $\mathbf{1b}^{+}\cdot BF_4^{-}$ from acetonitrile/diethyl ether solution afforded a single crystal (36.5 mg, 0.087 mmol, 64%) suitable for X-ray crystallographic analysis.

mp 243.5–244.0 ºC (decomp.); IR (ATR) v = 3110 (w), 2961 (m), 2914 (w), 2874 (w), 1574 (w), 1438 (m), 1397 (m), 1294 (w), 1225 (w), 1057 (s), 896 (w) cm^{-1}; ^{1}H NMR (400 MHz, $CDCl_3$) 9.72 (dd, J = 10.8, 2.0 Hz, 1H, 9-H), 955 (s, 1H, 7-H), 9.53 (d, J = 7.2 Hz, 1H, 10-H), 8.22 (d, J = 1.2 Hz, 1H, 3-H), 8.17 (d, J = 1.2 Hz, 1H, 5-H), 7.33 (s, 1H, 1-H), 1.71 (s, 9H, 19-H), 1.57 (s, 9H, 15-H), 1.50 (s, 9H, 17-H); $^{13}C\{^{1}H\}$ NMR (100 MHz, $CDCl_3$) 169.1 (s, C-8), 164.7 (s, C-11), 162.1 (s, C-4), 156.5 (s, C-12), 155.7 (d, C-7), 154.9 (d, C-9), 142.9 (s, C-2), 139.8 (s, C-6), 139.7 (d, C-10), 137.1 (d, C-3), 137.0 (s, C-13), 136.6 (d, C-1), 133.4 (d, C-5), 41.1 (s, C-18), 36.6 (s, C-16), 33.6 (s, C-14), 32.0 (q, C-19), 30.7 (q, C-15), 30.3 (q, C-17); $^{11}B\{^{1}H\}$ NMR (127 MHz, $CDCl_3$, $BF_3\cdot Et_2O$ in $CDCl_3$ as an external standard) –0.92; ^{19}F NMR (372 MHz, $CDCl_3$, $BF_3\cdot Et_2O$ in $CDCl_3$ as an external standard) –152.2; HRMS (MALDI-TOF-MS) Calculated: ($C_{25}H_{33}$) 333.2577 ($[M]^{+}$), Found: 333.2571.

^{1}H NMR (400 MHz, $CDCl_3$)

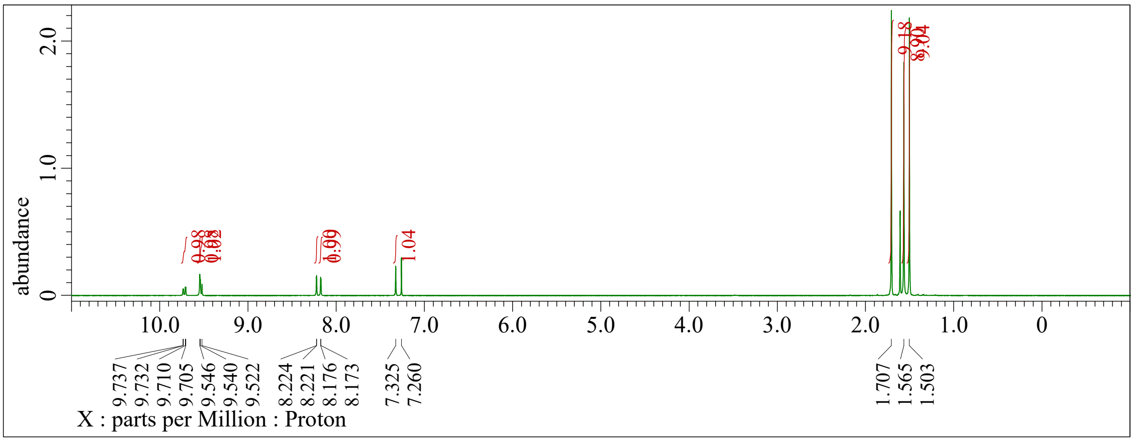

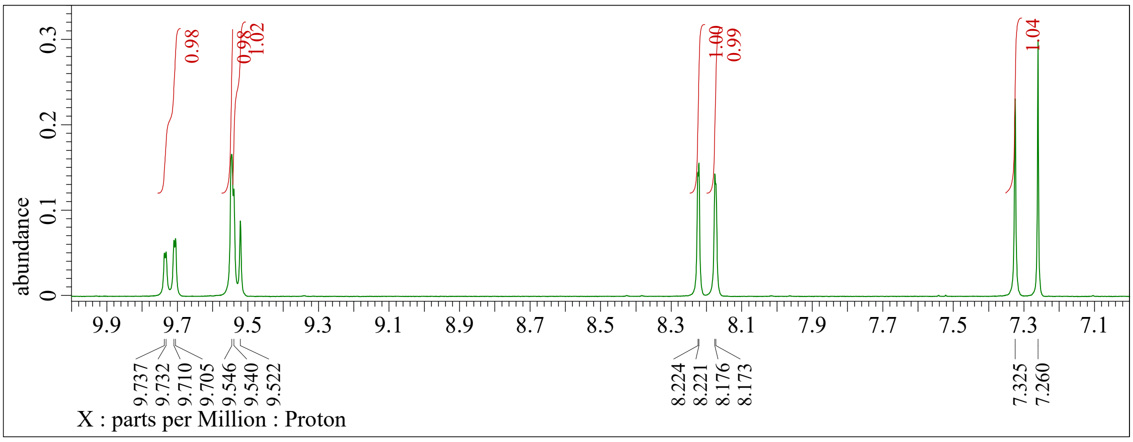

$^{13}C\{^1H\}$ NMR (100 MHz, $CDCl_3$)

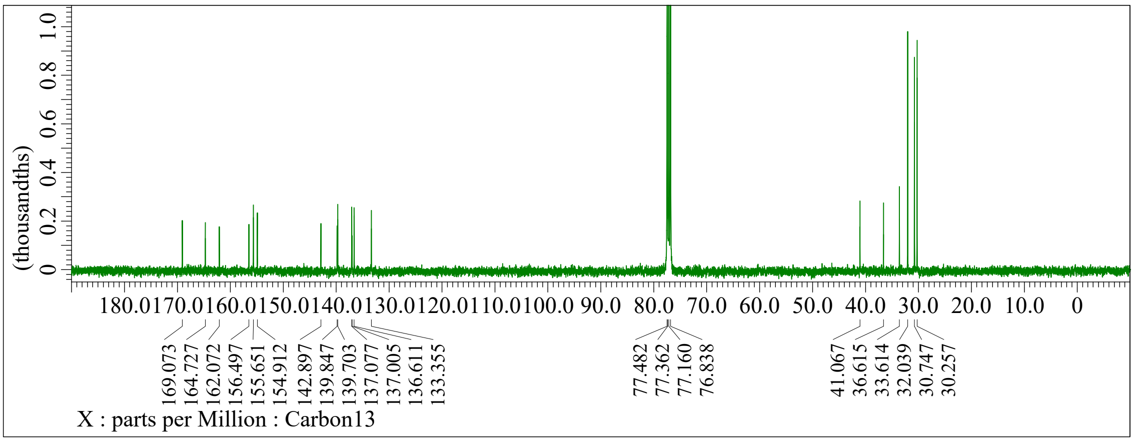

$^{11}B\{^1H\}$ NMR (127 MHz, $CDCl_3$, $BF_3{\cdot}Et_2O$ in $CDCl_3$ as an external standard)

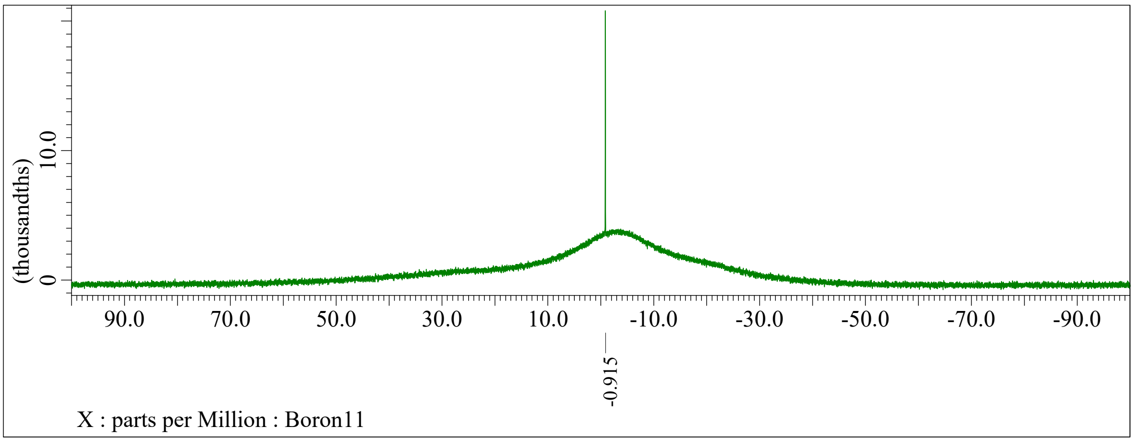

^{19}F NMR (372 MHz, $CDCl_3$, $BF_3 \cdot Et_2O$ in $CDCl_3$ as an external standard)

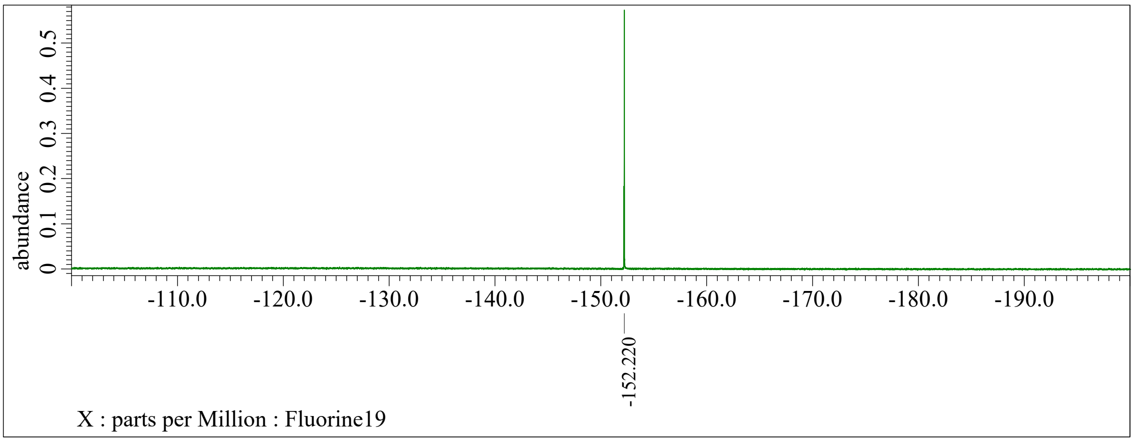

2D HSQC NMR (400 MHz, $CDCl_3$)

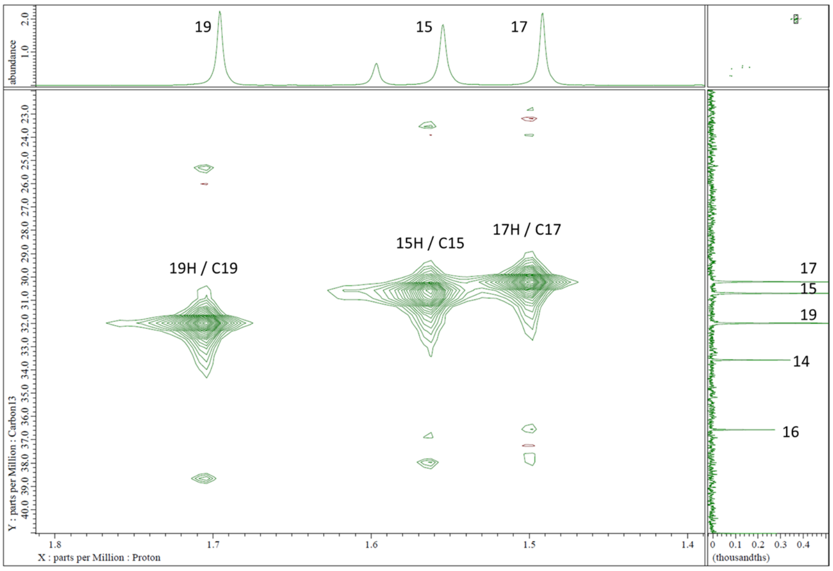

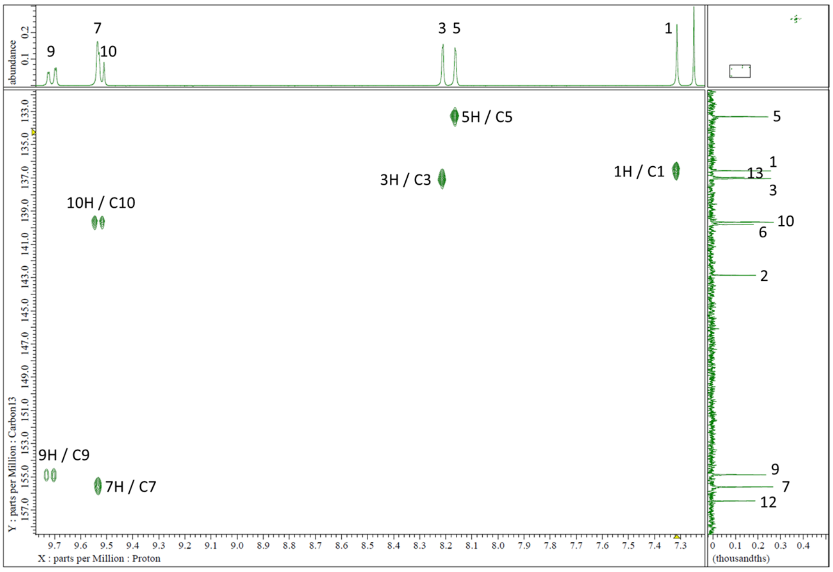

2D HMBC NMR (400 MHz, $CDCl_3$)

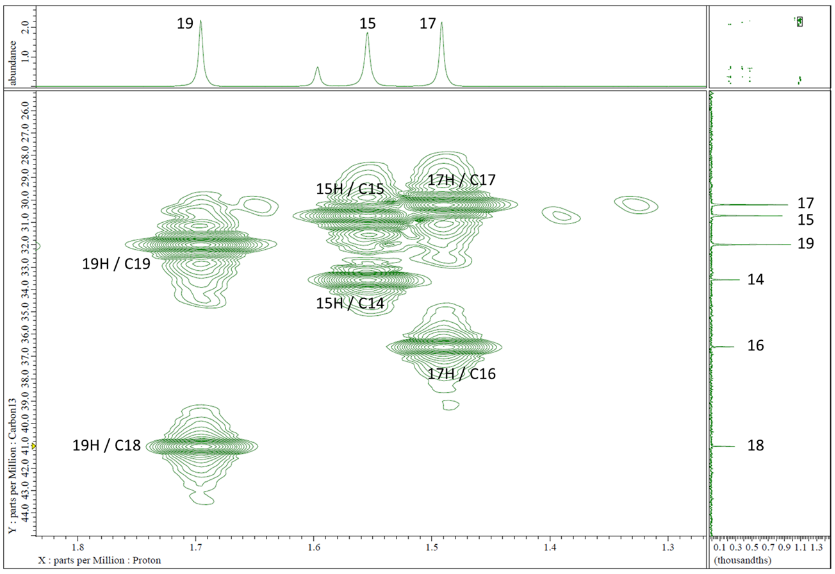

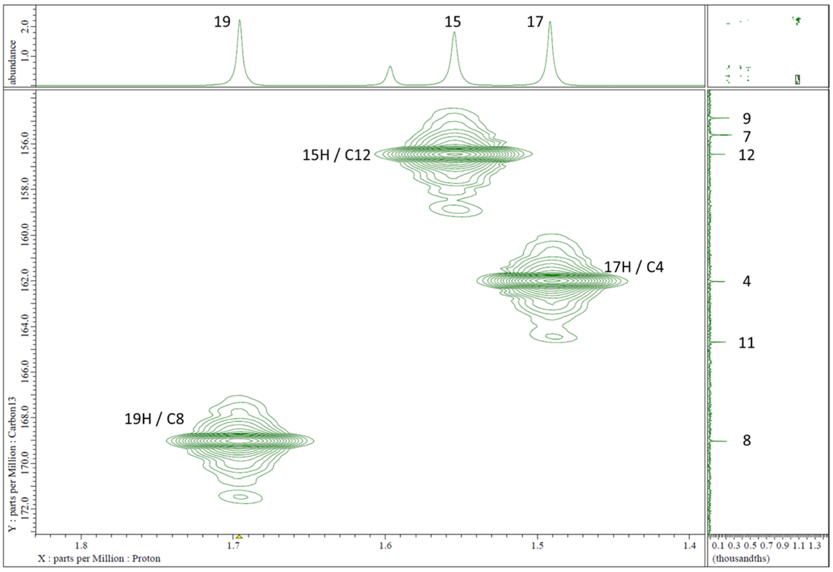

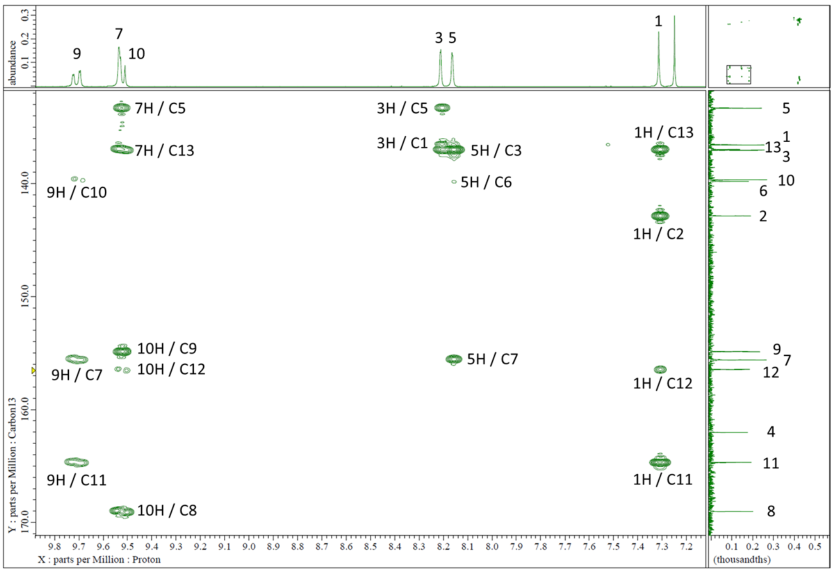

(6*R)-1,1',4,4',7,7'-Hexa-*tert*-butyl-6*H*,6'*H*-6,6'-bibenzo[*cd*]azulene 1b$_2$**

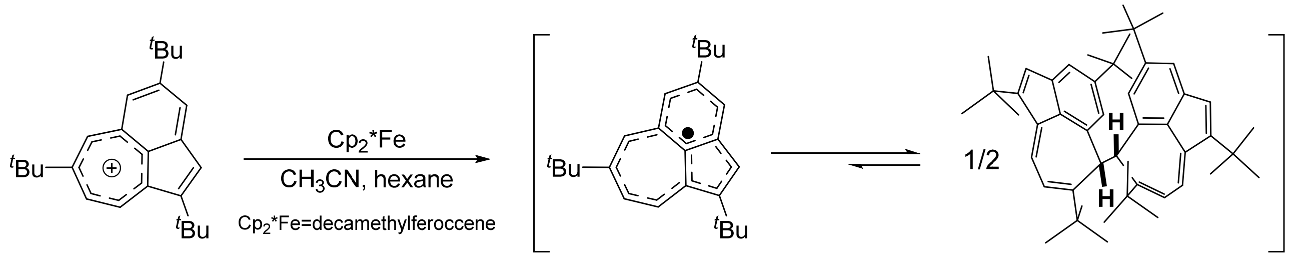

1b^{+}·BF$_4^{-}$ **1b** **1b$_2$**

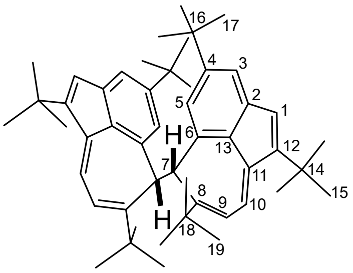

In a nitrogen-filled glovebox, a solution of decamethylferrocene (26.8 mg, 0.0821 mmol) in hexane (2 mL) was added to a solution of **1b**$^{+}$·BF_4^{-} (36.5 mg, 0.0867 mmol) in CH_3CN (4 mL). The mixture was stirred at room temperature for 1 h. The solvents were removed in vacuum, and the obtained solids were dissolved in hexane (10 mL) and insoluble materials were removed by filtration through a cotton. The filtrate was evaporated to obtain **1b**$_2$ as a yellow solid (28.9 mg, quant). The recrystallization of **1b**$_2$ from a benzene-ethanol solution outside afforded a single crystal suitable for the X-ray crystallographic analysis.

mp 234.2–235.0 °C; IR (ATR) ν = 3052 (w), 2956 (s), 2901 (m), 1476 (m), 1361 (m), 1239 (m), 1051 (w), 881 (m), 858 (m) cm^{-1}; ^{1}H NMR (400 MHz, $CDCl_3$) 7.22 (d, J = 7.2 Hz, 2H, 10-H), 6.76 (d, J = 1.2 Hz, 2H, 3-H), 6.45 (s, 2H, 1-H), 6.44 (d, J = 6.0 Hz, 2H, 9-H), 5.66 (d, J = 1.2 Hz, 2H, 5-H), 3.91 (s, 2H, 7-H), 1.50 (s, 18H, 15-H), 1.08 (s, 18H, 19-H), 1.02 (s, 18H, 17-H); ^{13}C{^{1}H} NMR (100 MHz, $CDCl_3$) 152.1 (s, C-12), 152.1 (s, C-4), 150.6 (s, C-8), 141.4 (s, C-2), 138.7 (s, C-11), 131.6 (s, C-6), 130.1 (s, C-13), 125.9 (d, C-10), 125.9 (d, C-1), 121.9 (d, C-5), 119.9 (d, C-9), 116.3 (d, C-3), 42.1 (d, C-7), 38.3 (s, C-18), 34.3 (s, C-16), 33.2 (s, C-14), 31.5 (q, C-15), 31.5 (q, C-19), 31.2 (q, C-17); HRMS (ESI^{+}) σ-dimer **1b**$_2$ Calculated: ($C_{50}H_{66}Na$) 689.5057 ([M + Na]$^{+}$), Found: 689.5047. HRMS (ESI^{+}) monomer **1b** Calculated: ($C_{25}H_{33}$) 333.2577 ([M]$^{+}$), Found: 333.2567.

^{1}H NMR (400 MHz, $CDCl_3$)

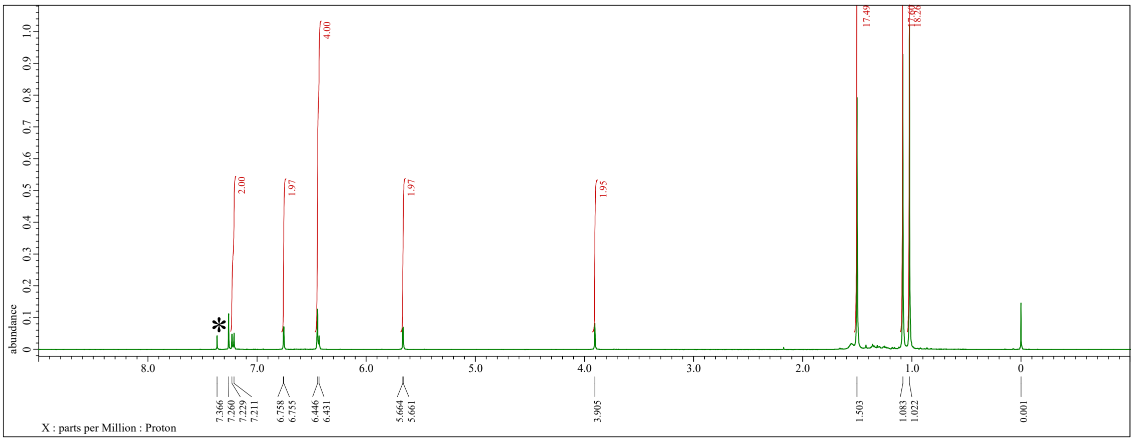

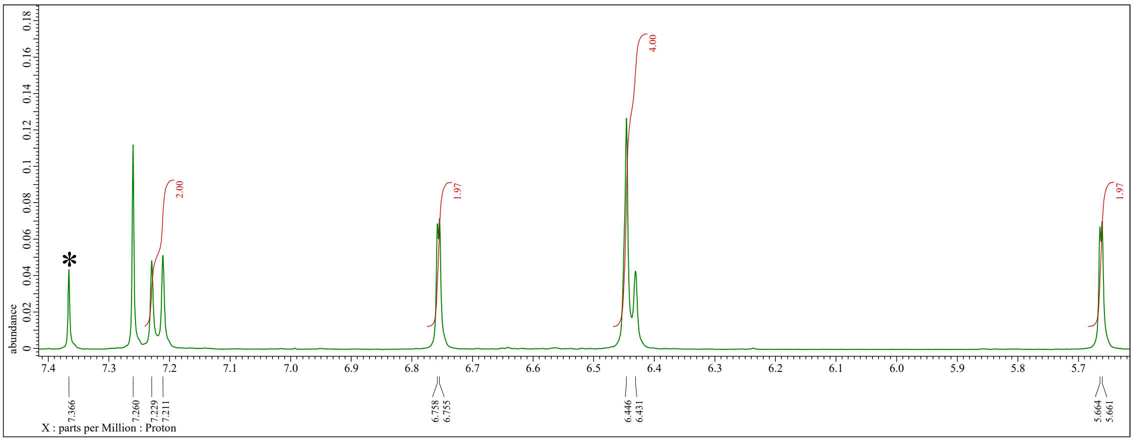

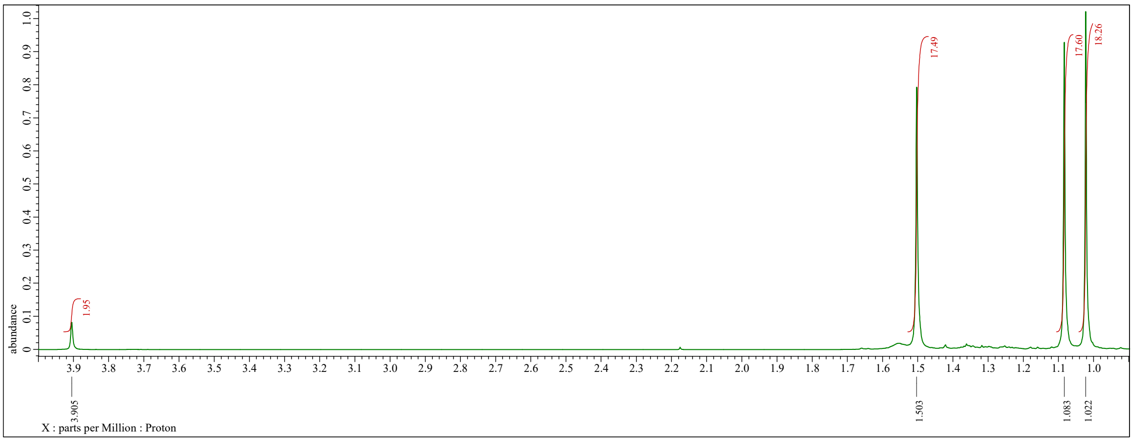

＊: An asterisk mark represents the signal of residual benzene for recrystallization.

$^{13}C\{^1H\}$ NMR (100 MHz, $CDCl_3$)

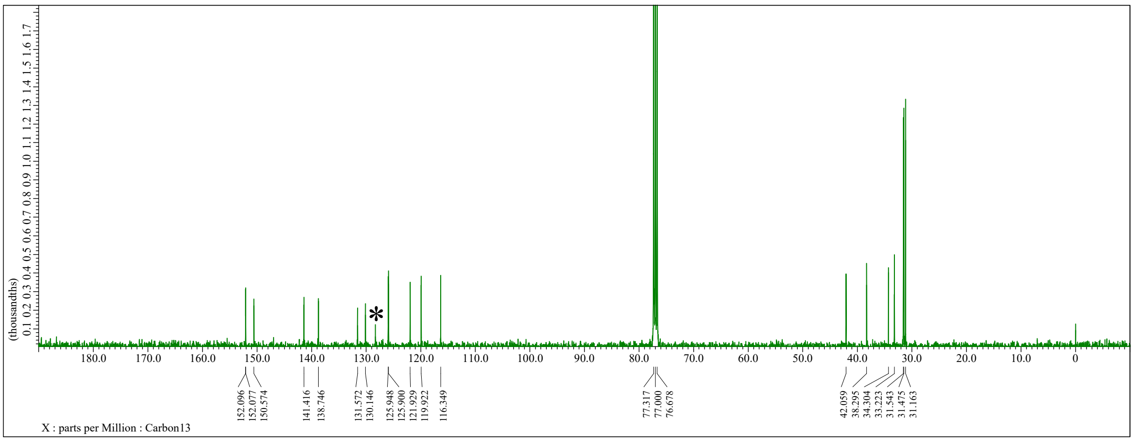

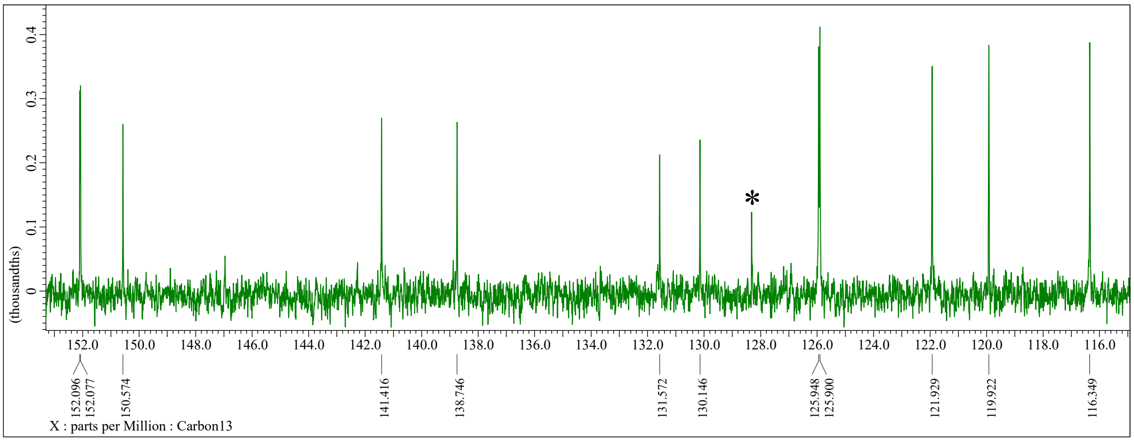

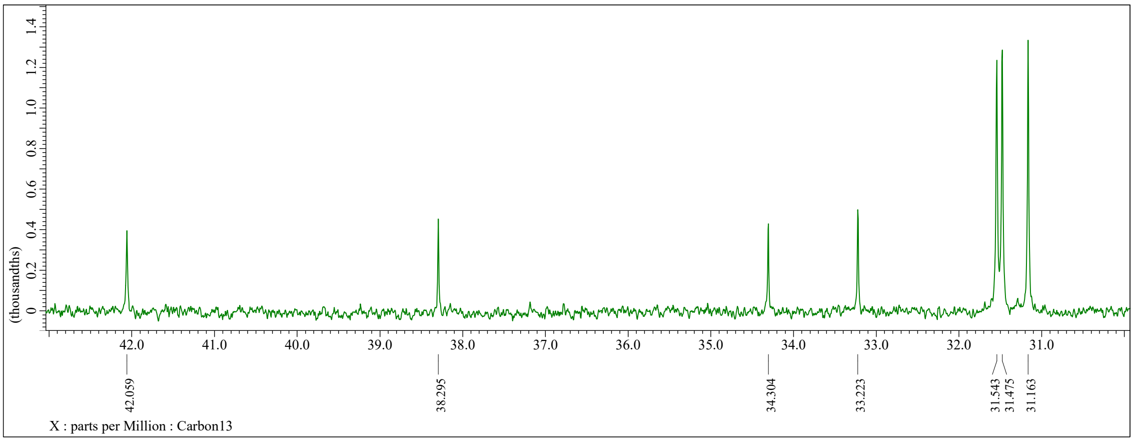

* : An asterisk mark represents the signal of residual benzene for recrystallization.

2D HSQC NMR (400 MHz, $CDCl_3$)

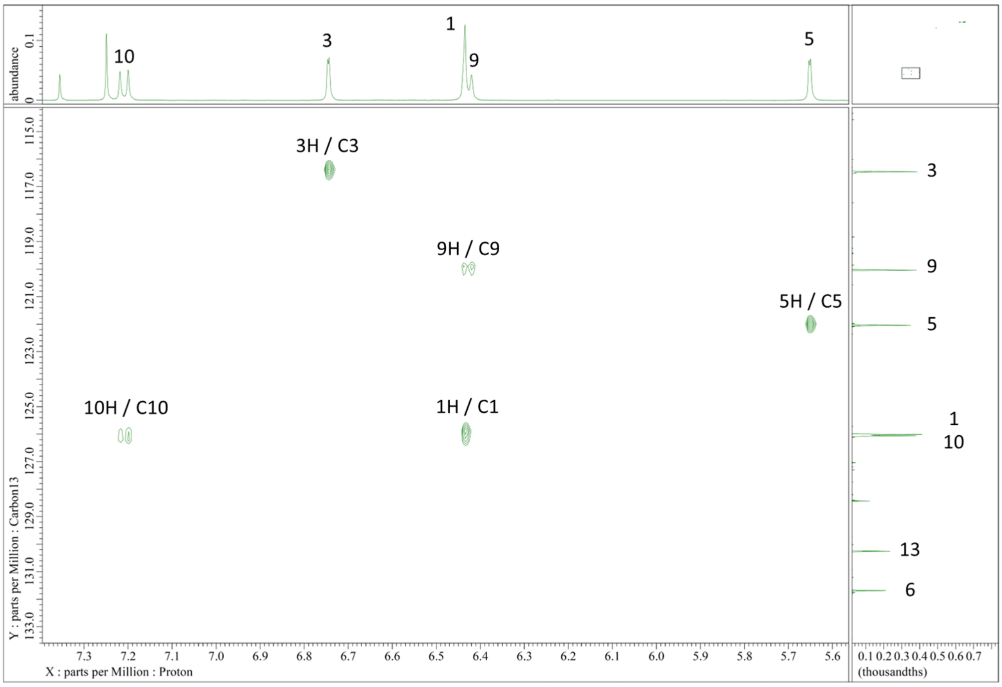

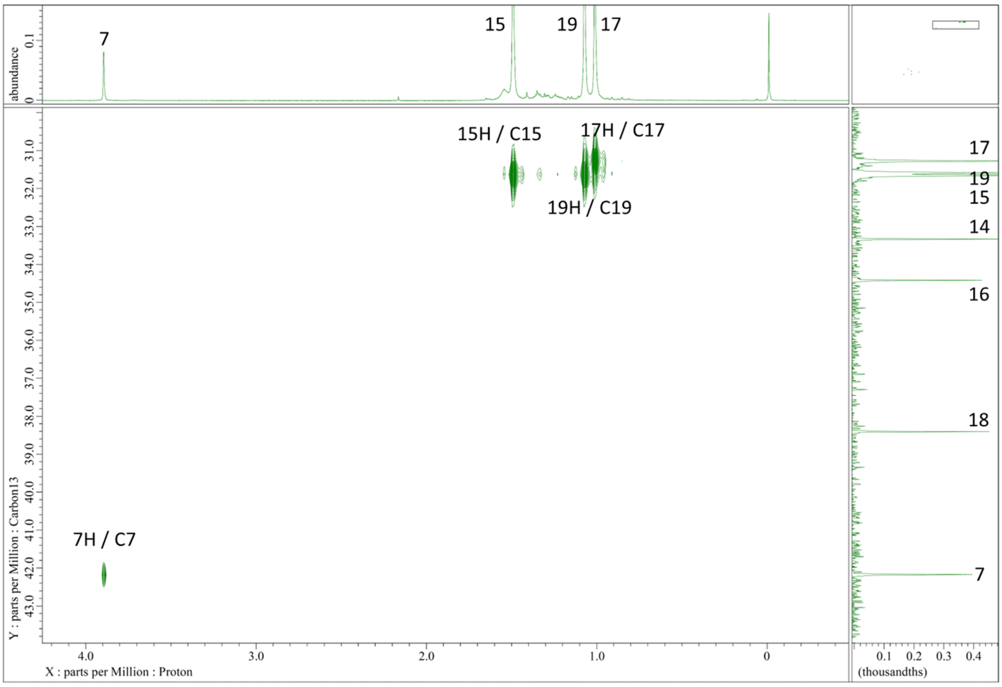

2D HMBC NMR (400 MHz, $CDCl_3$)

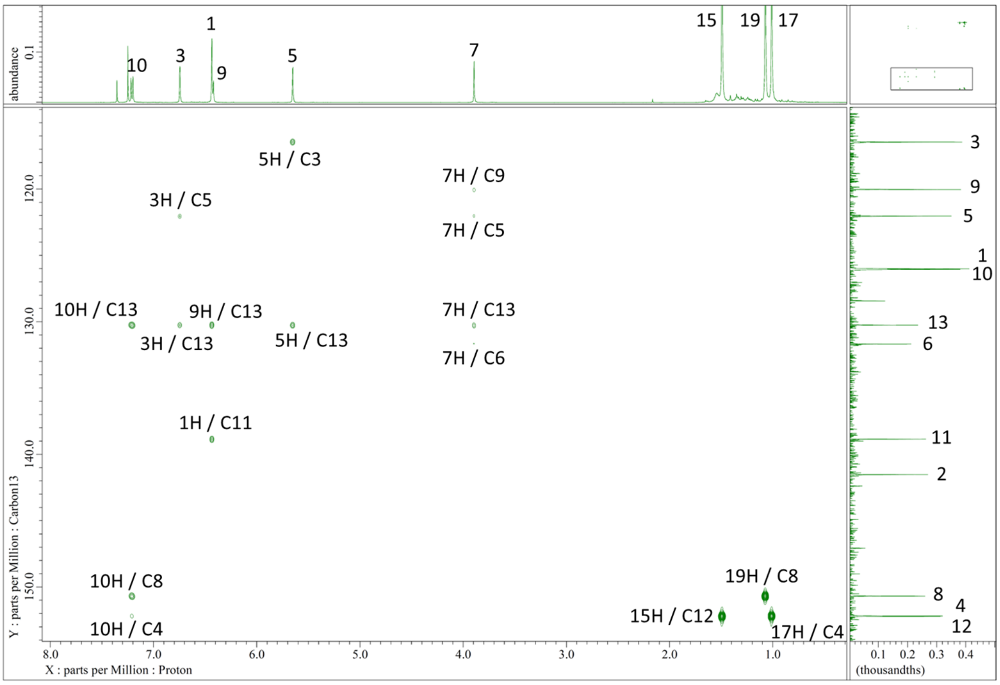

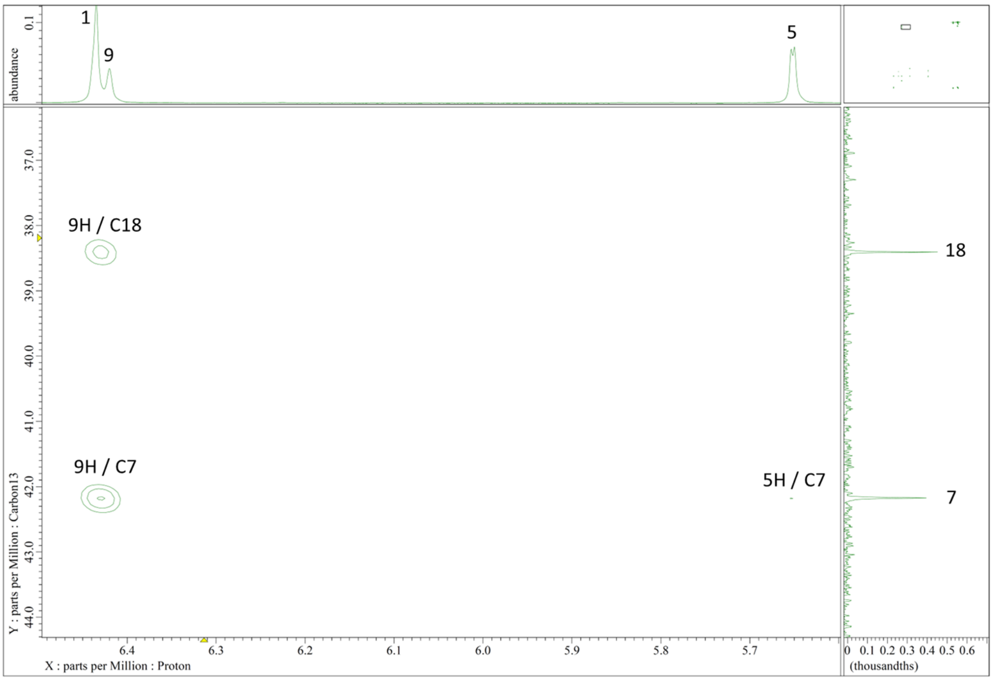

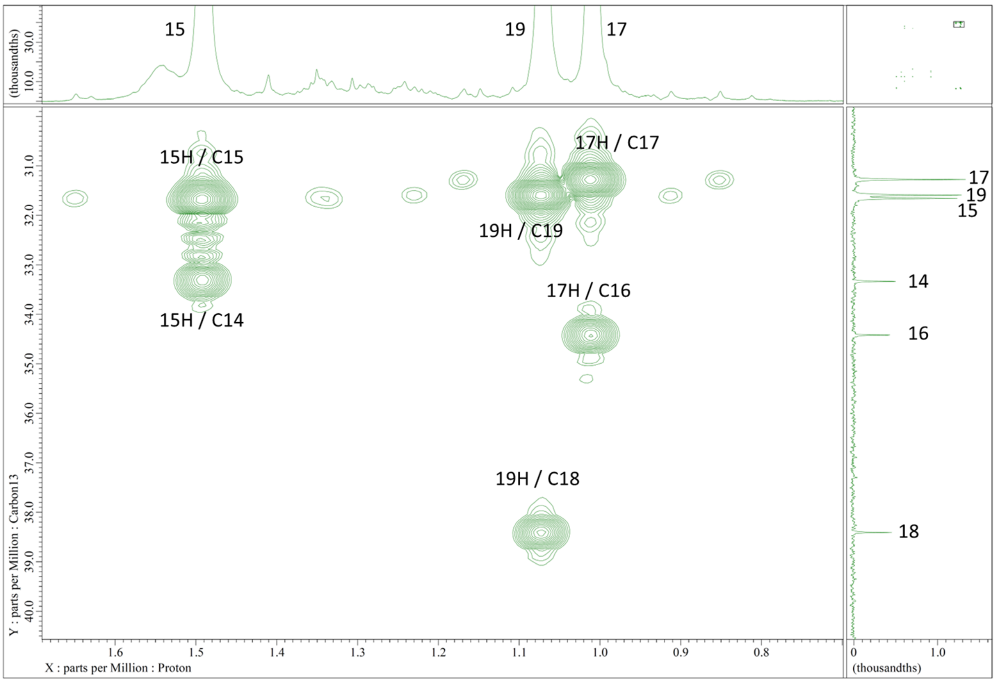
15
19
17
(thousandths)
15H / C15
17H / C17
19H / C19
15H / C14
17H / C16
19H / C18
17
19
15
14
16
18
Y : parts per Million : Carbon13
X : parts per Million : Proton
(thousandths)

4. Stability of 1b and $1b_2$

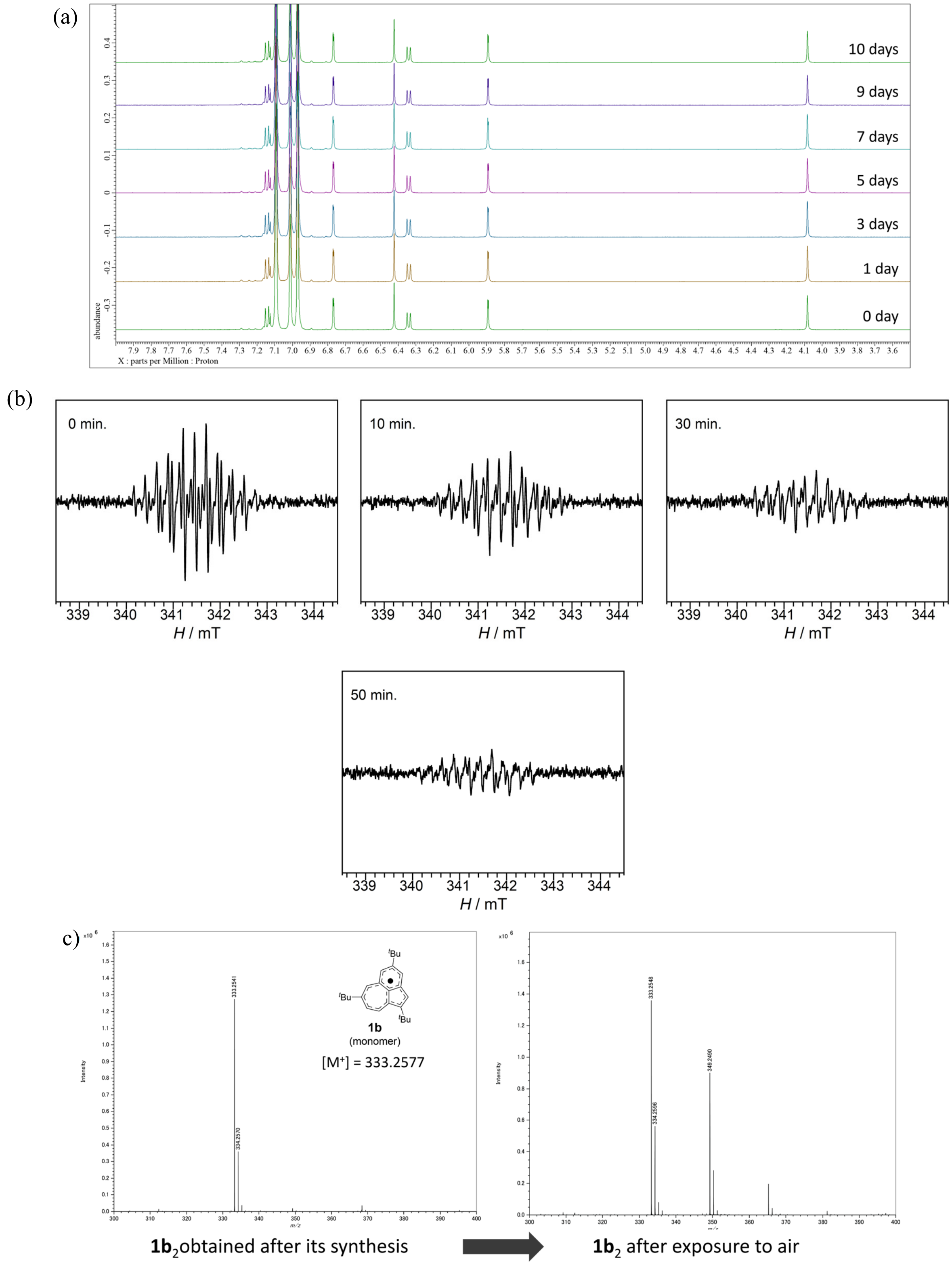

Figure S1. (a) Periodical ^{1}H NMR (400 MHz, toluene-d_8) spectra of $\mathbf{1b_2}$ upon exposure to air under indoor light at room temperature and (b) Periodical ESR (toluene) spectra of **1b** upon exposure to air at room temperature under dark conditions. (C) Changes in MALDI-TOF-MS spectra of $\mathbf{1b_2}$. Under the MALDI conditions, the molecular ion peak was observed as monomer **1b**.

5. X-ray crystallographic data

5-1. Summary for crystallographic data of 1b^{+}·BF_4^{-}

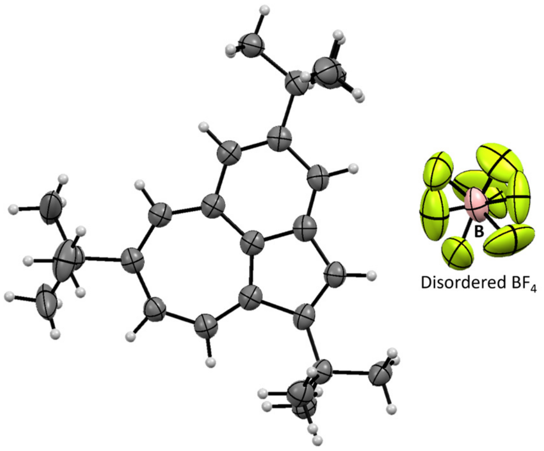

Figure S2. ORTEP drawing of **1b**$^{+}$·BF_4^{-}. The thermal ellipsoids are set at the 50% probability level.

No. CCDC	2520797		
Empirical Formula	$C_{25}H_{33}BF_4$	*Z* value	4
Formula Weight	420.32	D_{calc} /g·cm^{-3}	1.211
Crystal Color, Habit	green, block	F_{000}	896.0
Crystal Dimensions / mm	0.19 × 0.16 × 0.12	μ(CuK_{α}) / mm^{-1}	0.747
Crystal System	monoclinic	Data/restraints/parameters	4571/90/317
Space Group	$P2_1/n$ (#14)	Residuals: *R*1	0.0790
a / Å	9.9572(3)	($I > 2.00\sigma(I)$)	
b / Å	14.0723(5)	Residuals: *wR*2	0.2325
c / Å	16.5849(6)	(*all data*)	
α / °	90	Goodness of Fit Indicator	1.044
β / °	97.305(3)	Recrystallization from	CH_3CN/Et_2O
γ / °	90		
Volume / Å^3	2305.03(14)		
Temperature / K	123		

5-2. Summary for crystallographic data of 1b$_2$

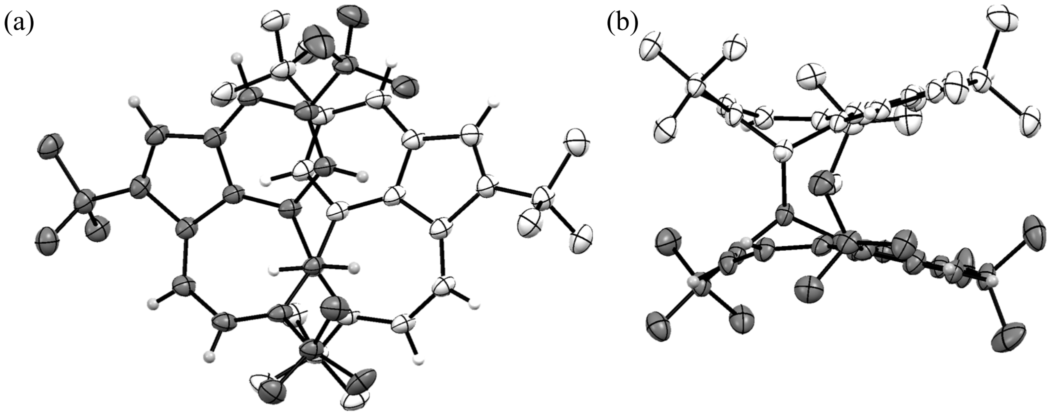

Figure S3. ORTEP drawing of (a) top and (b) side views for **1b**$_2$. The thermal ellipsoids are set at the 50% probability level. Some hydrogen atoms are omitted for clarity.

No. CCDC	2520798		
Empirical Formula	$C_{50}H_{66}$	Z value	2
Formula Weight	667.02	D_{calc} /g·cm^{-3}	1.058
Crystal Color, Habit	yellow, block	F_{000}	732.0
Crystal Dimensions / mm	0.22 × 0.18 × 0.17	μ(CuK$_\alpha$) / mm^{-1}	0.433
Crystal System	triclinic	Data/restraints/parameters	8237/0/469
Space Group	P-1 (#2)	Residuals: R1	0.0794
a / Å	10.6249(5)	($I > 2.00\sigma(I)$)	
b / Å	12.8381(5)	Residuals: wR2	0.2417
c / Å	17.3764(7)	(*all data*)	
α / °	80.967(4)	Goodness of Fit Indicator	1.099
β / °	73.742(4)	Recrystallization from	EtOH/benzene
γ / °	67.180(4)		
Volume / Å^3	2094.18(17)		
Temperature / K	123		

6. Summary of the observed geometries

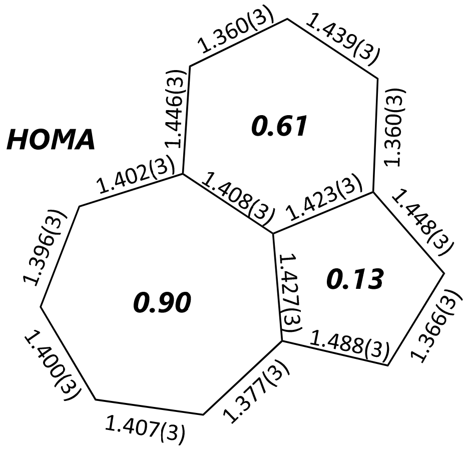

Figure S4. Summary for the observed bond lengths (/ Å) and HOMA values for **1b**$^{+}\cdot BF_4^{-}$ at 123 K.

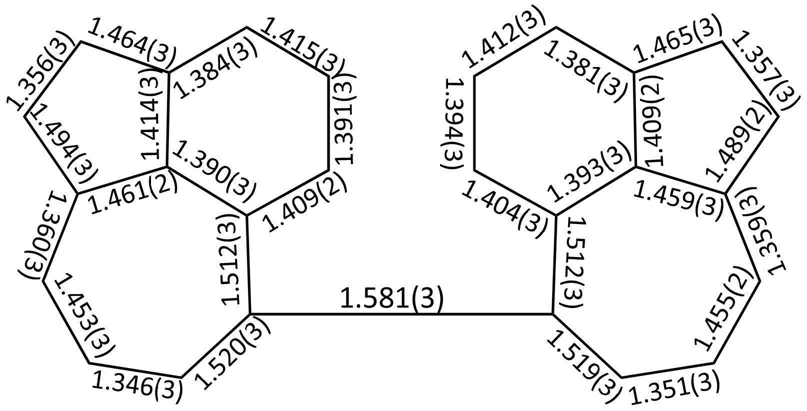

Figure S5. Summary for the observed bond lengths (/ Å) for $\mathbf{1b}_2$ at 123 K.

7. ESR measurements

7-1. ESR spectrum of 1b

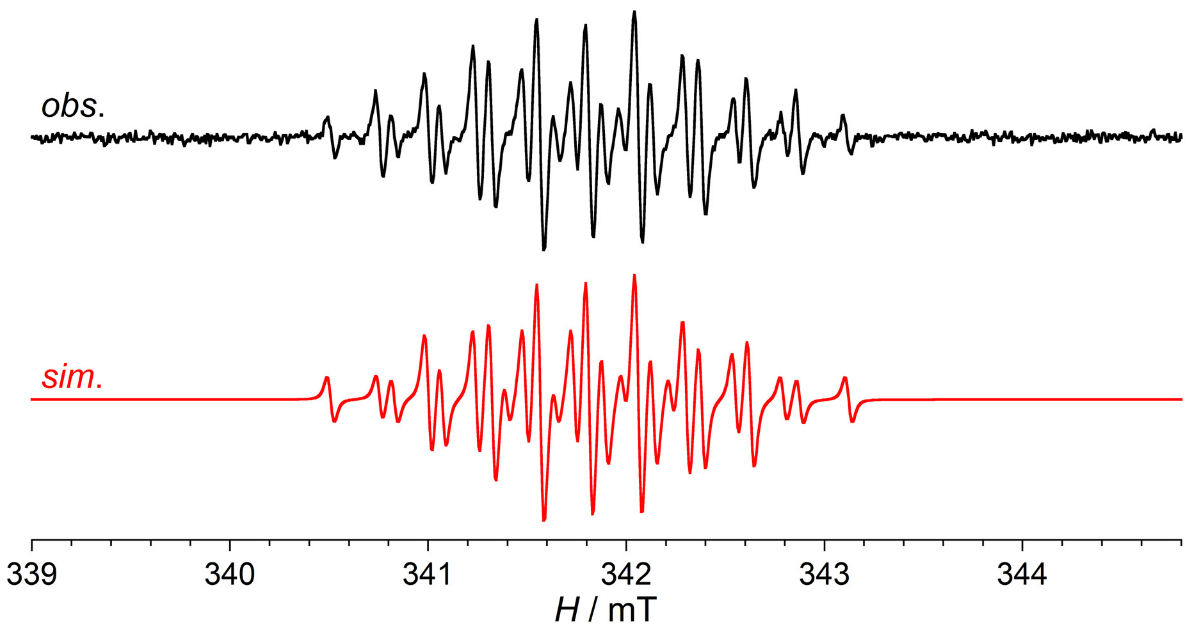

Figure S6. ESR spectrum of a hexane solution of $\mathbf{1b}_2$ (~10^{-4} M) at 300 K. The microwave frequency used was 9.581430 GHz. The value of the *g*-value was determined as 2.0027. Gain = 20000, sweep time = 3.1 min, modulation amplitude = 0.01 mT.

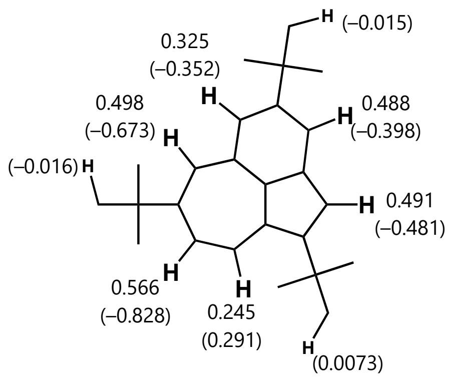

Figure S7. Summary of the hyper-fine coupling constants ($|a_H|$/mT) for **1b**. The values in the parentheses were calculated by the UB3LYP/EPR-II//UB3LYP-D3(BJ)/6-311G(d) method.

7-2. VT-ESR measurement of 1b

In a nitrogen-filled glovebox, a toluene solution of compound **1b**$_2$ was prepared at a concentration of 0.732 mM and placed into a J-Young quartz ESR tube with a diameter of 4 mm. The spin concentration of **1b** in the solution at various temperatures was determined using the ESR signal of a DPPH solution in toluene at the corresponding temperature, which served as an external reference. A toluene solution of DPPH was prepared at a concentration of 0.276 mM under inert conditions, and the same volume of this solution was used as that of **1b**$_2$, which was also placed in the same ESR tube.

To avoid saturation of the ESR signal, we examined the dependence of the ESR signal intensity on microwave power at 300 K (Figure S8). The signal intensities of both **1b** and DPPH increased linearly as a function of the square root of the microwave power, ranging from 2.0 µW to 16 µW, without reaching saturation. Consequently, the microwave power for the ESR measurements was selected to be 8.0 µW (= 2.83 $(\mu W)^{1/2}$).

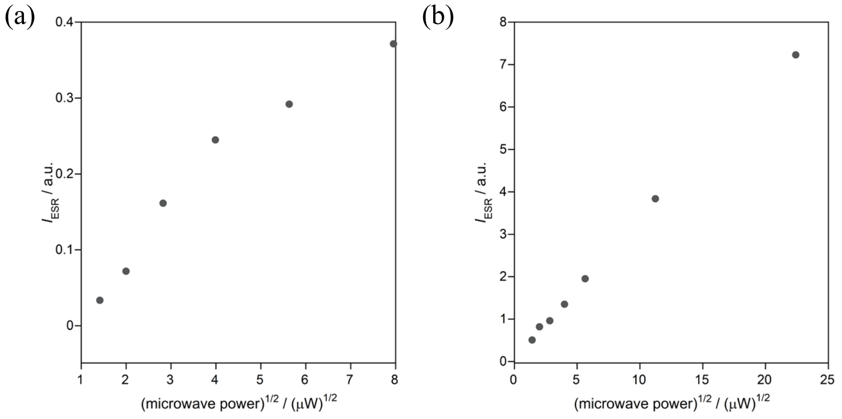

Figure S8. Correlation between ESR intensities and square root of microwave powers for (a) **1b** and (b) DPPH at 300 K.

After heating the sample from 240 K to 380 K, it was allowed to cool back to room temperature to evaluate the measurement's reproducibility. The ESR spectra at various temperatures are shown in Figure S9. The determined concentrations of **1b** and **1b**$_2$, along with the equilibrium constant for the dimerization of **1b** (K_{dimer}), are summarized in Table S1.

$$\text{2 monomer } (\mathbf{1b}) \overset{K_{dimer}}{\rightleftharpoons} \text{dimer } (\mathbf{1b}_2)$$

$$K_{dimer} = \frac{[\mathbf{1b}_2]}{[\mathbf{1b}]^2}$$

Finally, according to the van’t Hoff plot, the thermodynamic parameters of the dimerization were calculated by the least squares procedure.

$$\ln K_{dimer} = -\frac{\Delta H}{RT} + \frac{\Delta S}{R}$$

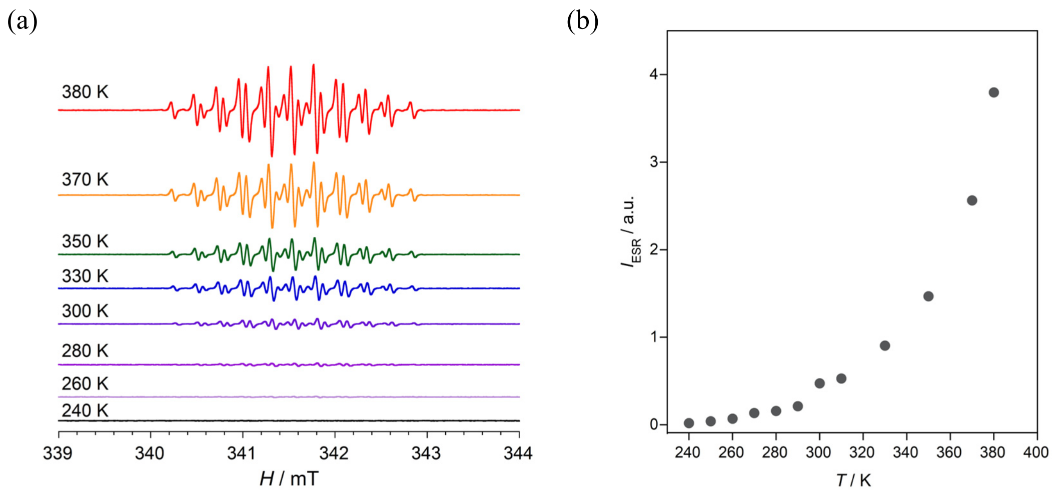

Figure S9. (a) Variable temperature ESR spectra for **1b** in toluene. (b) Temperature dependence of the ESR intensity (I_{ESR}) of **1b**.

Table S1. Summary for the equilibrium concentrations of **1b** and **1b**$_2$ determined from the ESR measurement at each temperature and the equilibrium constant, K_{dimer}, at the corresponding temperature.

Temp. / K	[**1b**] / mol·L^{-1}	[**1b**$_2$] / mol·L^{-1}	K_{dimer} / L·mol^{-1}
380	1.31×10^{-4}	6.66×10^{-4}	3.87×10^{4}
370	8.46×10^{-5}	6.90×10^{-4}	9.64×10^{4}
350	4.67×10^{-5}	7.09×10^{-4}	3.25×10^{5}
330	2.82×10^{-5}	7.18×10^{-4}	9.00×10^{5}
310	1.64×10^{-5}	7.24×10^{-4}	2.68×10^{6}
300	1.42×10^{-5}	7.25×10^{-4}	3.59×10^{6}
290	6.62×10^{-6}	7.29×10^{-4}	1.66×10^{7}
280	4.96×10^{-6}	7.30×10^{-4}	2.97×10^{7}
270	4.28×10^{-6}	7.30×10^{-4}	3.99×10^{7}
260	2.25×10^{-6}	7.31×10^{-4}	1.44×10^{8}
250	1.30×10^{-6}	7.31×10^{-4}	4.30×10^{8}
240	5.99×10^{-7}	7.32×10^{-4}	2.04×10^{9}

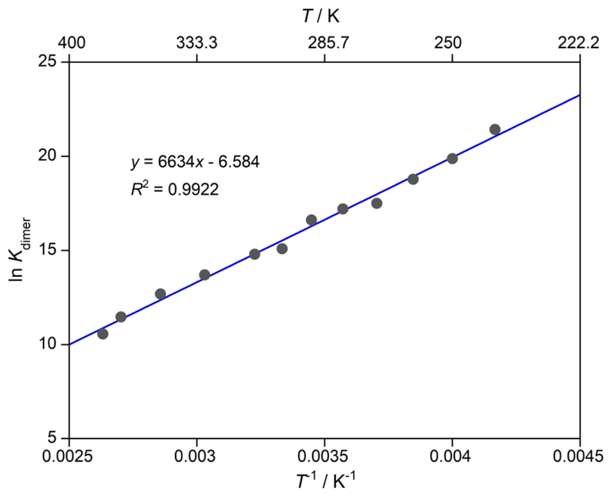

Figure S10. Temperature dependence of the dimerization constant K_{dimer}. According to the van’t Hoff plot, the thermodynamic parameters of the dimerization were calculated by the least squares procedure, to give $\Delta H = -13.2$ kcal·mol^{-1}, $\Delta S = -13.1$ cal·K^{-1}·mol^{-1}, and ΔG (298 K) = -9.29 kcal·mol^{-1}.

7-3. Evaluation of photo-stimulus dissociation of the σ-bond in $\mathbf{1b_2}$

In a nitrogen-filled glovebox, a toluene solution of compound $\mathbf{1b_2}$ was prepared at a concentration of 0.774 mM and placed into a J-Young quartz ESR tube with a diameter of 4 mm. To examine the photo-stimulus dissociation of $\mathbf{1b_2}$, the solution was placed in the ESR and set to the specified temperature. ESR measurements were conducted after subjecting the solution to photoirradiation with a 300 W Xe lamp for 5–7 minutes. The photoirradiation was carried out using an ASAHI SPECTRA MAX-351 Xe light source (300 W), which was equipped with a visible light mirror module without a light filter (385–740 nm). Following the photoirradiation, an ESR spectrum was immediately recorded at the specified temperature. Subsequently, periodic monitoring was conducted to assess the decay of the ESR signal intensity.

From the obtained decay profile of the ESR signal intensity, the reaction rate constant was estimated from the following self-dimerization reaction.

$$\mathbf{1b}_2 \xrightarrow[\text{5-7 min}]{\text{300 W Xe lamp}} 2\ \mathbf{1b} \xrightarrow[\text{dark}]{\boldsymbol{k}} \mathbf{1b}_2$$

Herein,

$$-\frac{d[\mathbf{1b}]}{dt} = k[\mathbf{1b}]^2$$

Thus,

$$\frac{1}{[\mathbf{1b}]} = \frac{1}{[\mathbf{1b}]_0} + kt$$

Because the concentration of **1b** (= [**1b**]) is proportional to the observed intensity of the ESR spectrum, the following equation is obtained.

$$\frac{1}{Int_{ESR}} = \frac{1}{I_{ESR0}} + kt$$

The plots based on the above equation furnished the linear relationship. From the slope of the linear plot, the rate constant at the specified temperature was obtained.

Finally, according to the Eyring–Polanyi and Arrhenius plots, the kinetic parameters of the dimerization were calculated by the least squares procedure.

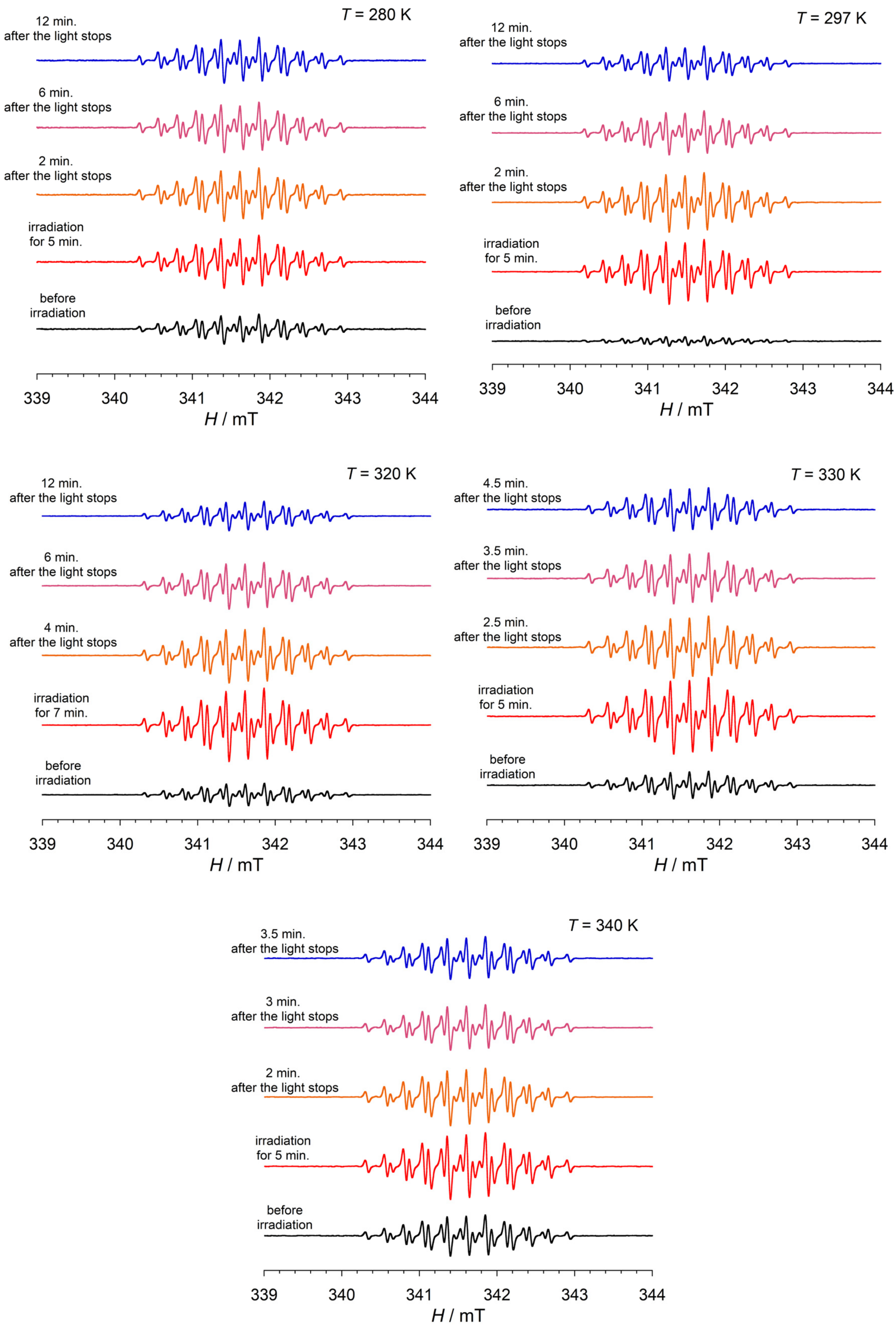

Figure S11. Time-dependent changes in the ESR spectra at the specified temperatures.

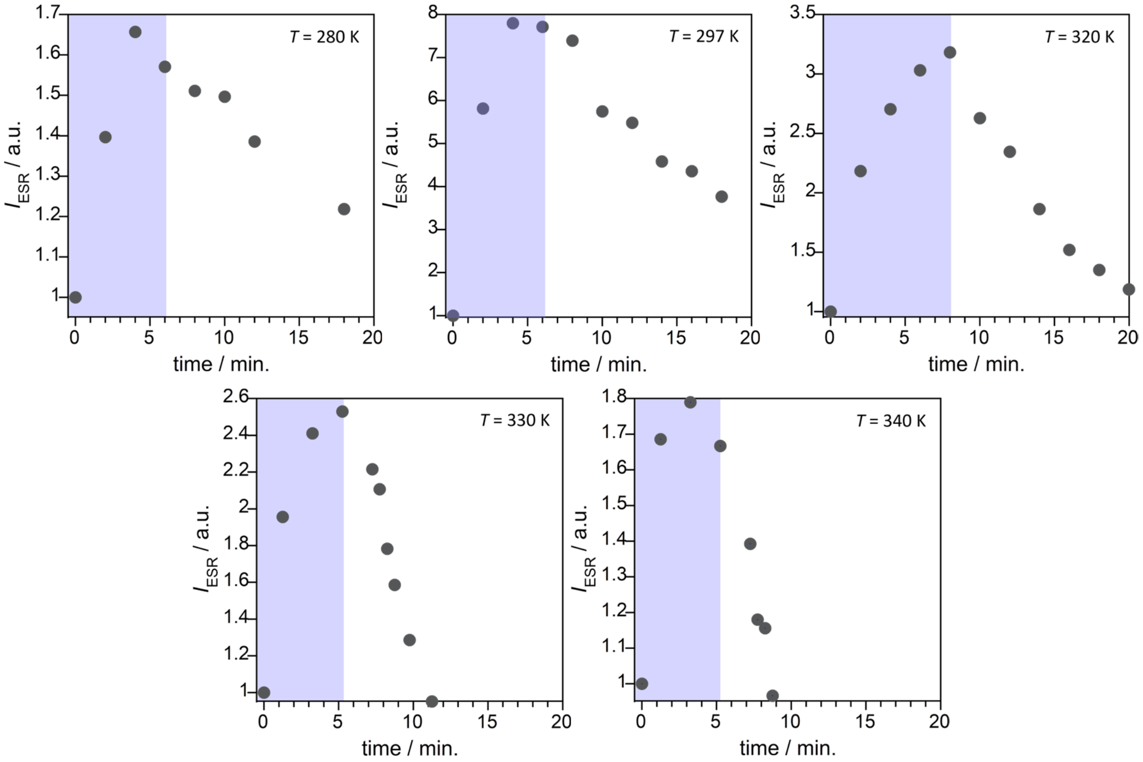

Figure S12. Profiles of ESR signal intensity at the specified temperatures. During the purple-colored period (the first 5 or 7 minutes), a Xe lamp was turned on.

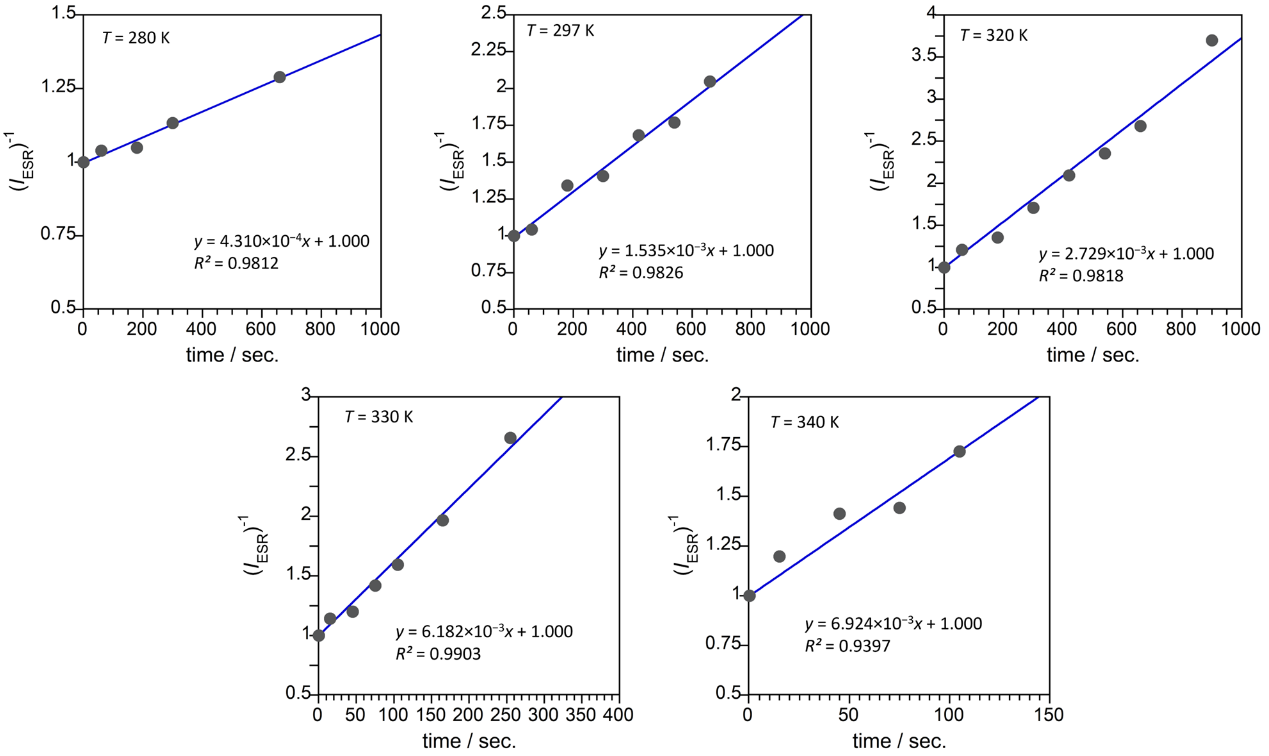

Figure S13. Decay profiles of ESR signal intensity after stopping photo-irradiation at the specified temperatures. From the slope of the linear plot, the rate constant at the specified temperature was obtained.

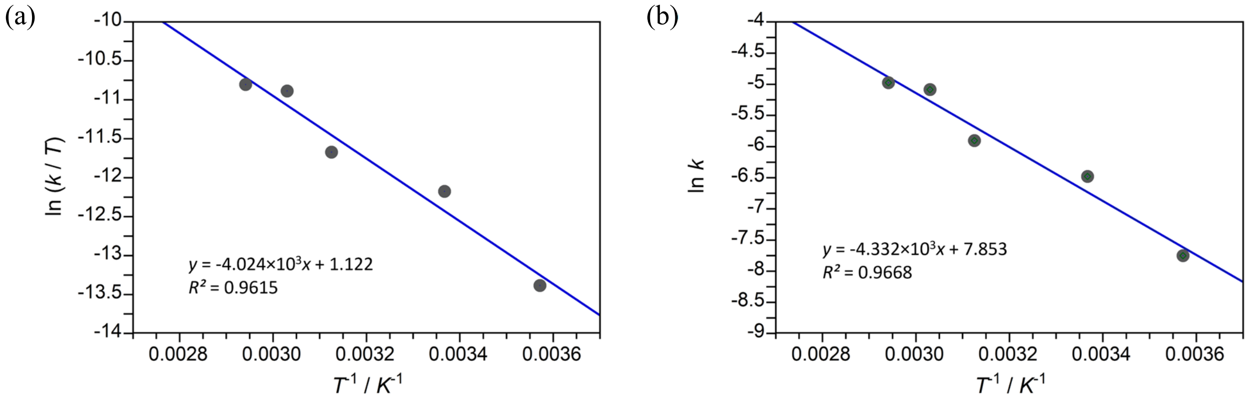

Figure S14. (a) Eyring–Polanyi and (b) Arrhenius plots for the dimerization of **1b**.

Table S2. Summary for the kinetic parameters of the dimerization of **1b** to **1b**$_2$.

	280 K	297 K	320 K	330 K	340 K
k / $M^{-1}s^{-1}$	0.000431	0.00154	0.00273	0.00618	0.00692

$\Delta H^{‡}$	$\Delta S^{‡}$	$\Delta G^{‡}$ (298 K)	E_a
8.00 kcal·mol^{-1}	−45.0 cal·K^{-1}·mol^{-1}	21.4 kcal·mol^{-1}	8.61 kcal·mol^{-1}

The use of the estimated reaction rate constants (k) for the radical recombination of **1b** (Table S2) and thermodynamic parameters for the σ-dimerization ($\Delta H = -13.2$ kcal·mol^{-1}, $\Delta S = -13.1$ cal·K^{-1}·mol^{-1} in Figure S10) affords the rate constants for the reverse reaction (k') by following the below equation. The obtained results are summarized in Table S3.

$$2\ \mathbf{1b} \underset{k'}{\overset{k}{\rightleftharpoons}} \mathbf{1b}_2 \qquad K_{dimer} = \frac{k}{k'}$$

Table S3. Summary for the kinetic parameters for the dissociation of $\mathbf{1b}_2$ to **1b**.

T / K	ΔG / kJ·mol^{-1} [a)]	K_{dimer} / M^{-1}	k / M^{-1}s^{-1}	k'/ s^{-1}
280	−39.8	2.69×10^{7}	0.000431	1.60×10^{-11}
297	−38.9	6.93×10^{6}	0.00154	2.22×10^{-10}
320	−37.6	1.39×10^{6}	0.00273	1.96×10^{-9}
330	−37.1	7.42×10^{5}	0.00618	8.33×10^{-9}
340	−36.5	4.11×10^{5}	0.00692	1.68×10^{-8}

a) The Gibbs-free-energy change (ΔG) for the σ-dimerization of **1b** at the sepcfic temperatures was estimated from the values of $\Delta H = -13.2$ kcal·mol^{-1}, $\Delta S = -13.1$ cal·K^{-1}·mol^{-1} in Figure S10.

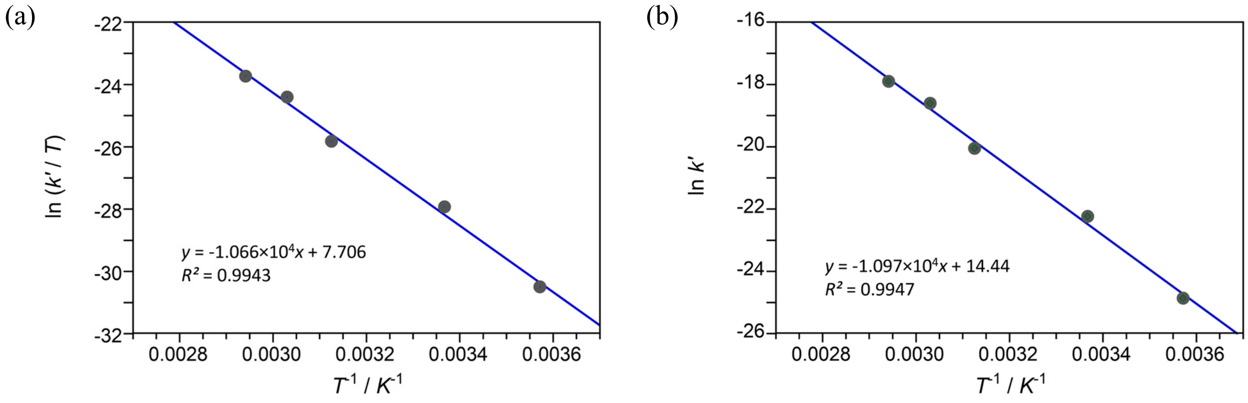

Figure S15. (a) Eyring–Polanyi and (b) Arrhenius plots for the dissociation of $\mathbf{1b}_2$ to **1b**.

Table S4. Summary for the kinetic parameters for the dissociation of $\mathbf{1b}_2$ to **1b**.

$\Delta H^{‡}$	$\Delta S^{‡}$	$\Delta G^{‡}$ (298 K)	E_a
21.2 kcal·mol^{-1}	−31.9 cal·K^{-1}·mol^{-1}	30.7 kcal·mol^{-1}	21.8 kcal·mol^{-1}

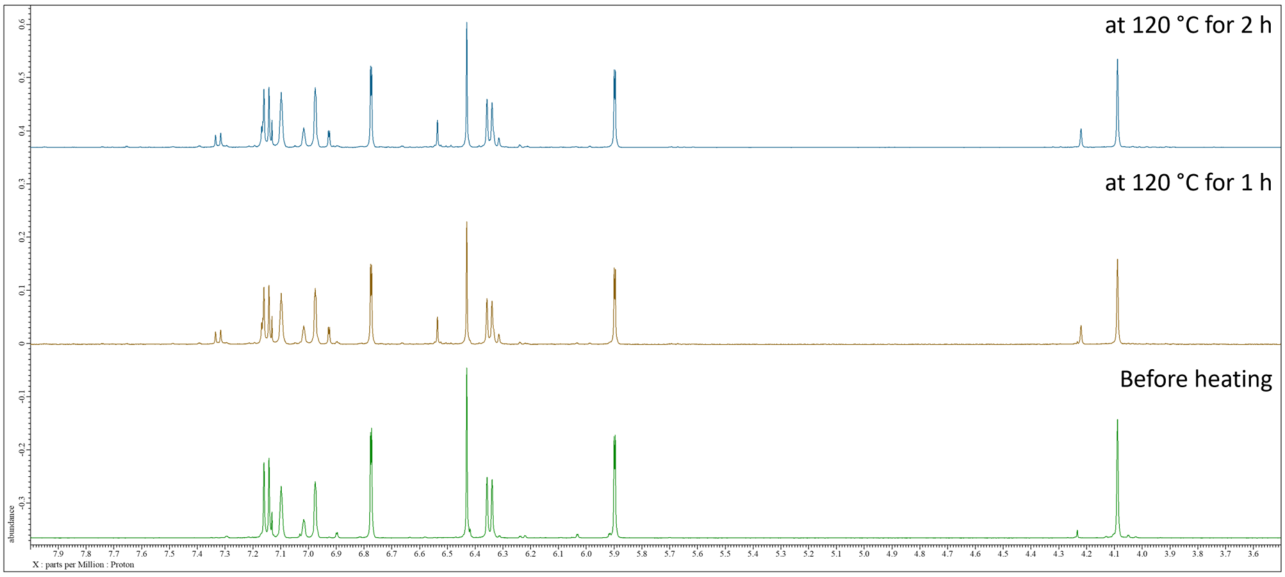

Figure S16. Periodical ^{1}H NMR (400 MHz, toluene-d_8) spectra of **1b**$_2$ under N_2 atmosphere at 120 °C.

8. Electrochemical properties

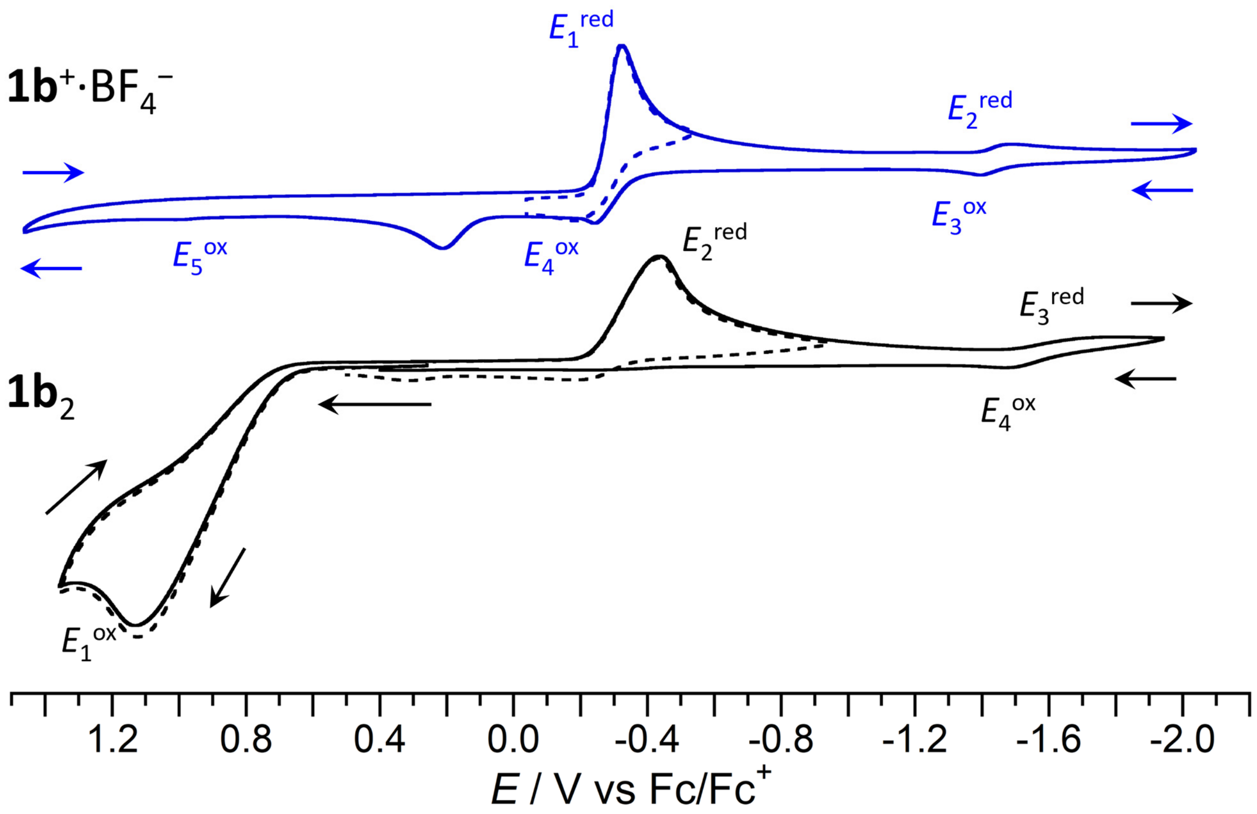

Figure S17. Cyclic voltammograms (CV) of **1b**$^{+}\cdot BF_4^{-}$ (V vs. Fc/Fc$^+$, in 0.1M nBu$_4$NClO$_4$/CH$_3$CN, scan rate = 100 mV/s, room temperature) and **1b**$_2$ (V vs. Fc/Fc$^+$, in 0.1M nBu$_4$NClO$_4$/THF, scan rate = 100 mV/s, room temperature).

Table S5. Summary for the redox potentials and events for **1b**$^{+}\cdot BF_4^{-}$ and **1b**$_2$ (V vs. Fc/Fc$^+$).

	E_5^{ox} / V	E_4^{ox} / V	E_1^{red} / V	E_2^{red} / V	E_3^{ox} / V	$E_{1/2}$ (E_1^{red}/ E_4^{ox})/ V	$E_{1/2}$ (E_2^{red}/E_3^{ox})/ V
	**1b$_2$→2 1b$^+$**	**1b→1b$^+$**	**1b$^+$→1b**	**1b→1b$^-$**	**1b$^-$→1b**	**1b$^+$↔1b**	**1b↔1b$^-$**
**1b**$^{+}\cdot BF_4^{-}$	+1.01	–0.24	–0.32	–1.49	–1.40	–0.28	–1.44
	E_1^{ox} / V		E_2^{red} / V	E_3^{red} / V	E_4^{ox} / V		$E_{1/2}$ (E_3^{red}/E_4^{ox})/ V
	**1b$_2$→2 1b$^+$**		**1b$^+$→1b**	**1b→1b$^-$**	**1b$^-$→1b**		**1b↔1b$^-$**
**1b**$_2$	+1.14		–0.44	–1.69	–1.47		–1.58

9. Electronic absorption spectra

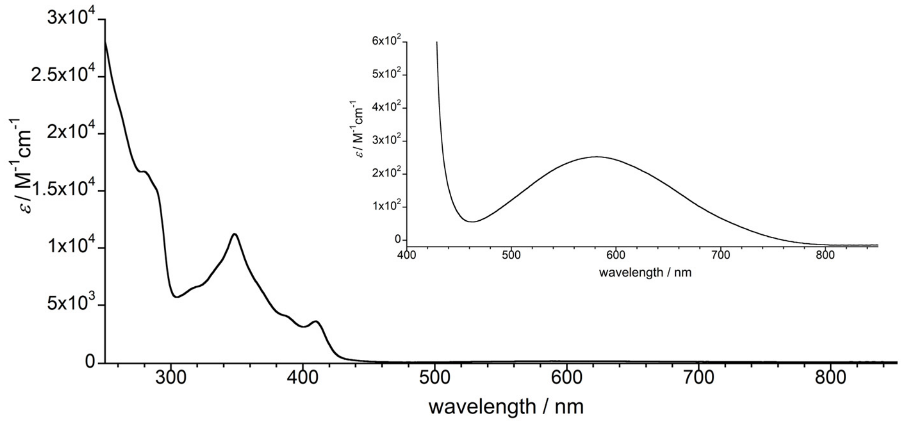

Figure S18. Electronic absorption spectra of **1b**$^+$$\cdot$$BF_4^-$ in CH_3CN.

λ / nm (ε / $M^{-1}cm^{-1}$): 594 (248), 410 (3590), 348 (11200), 281 (16600), 248 (28400).

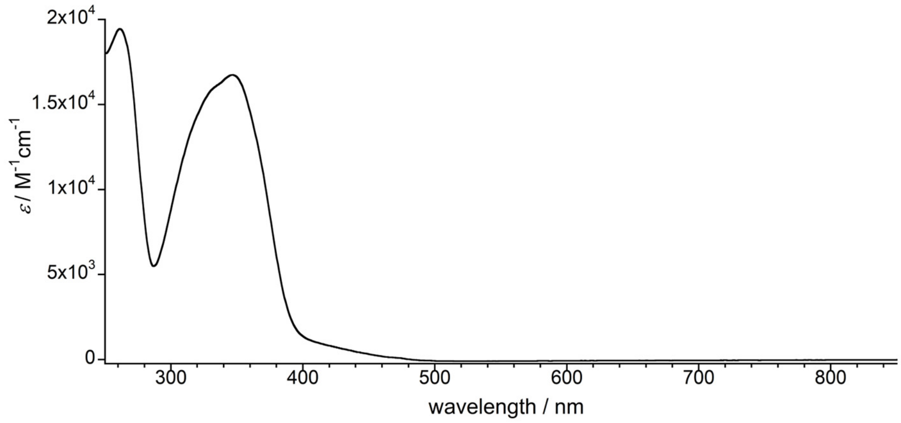

Figure S19. Electronic absorption spectra of **1b**$_2$ in hexane at room temperature.

λ / nm (ε / $M^{-1}cm^{-1}$): 347 (16700), 262 (19400).

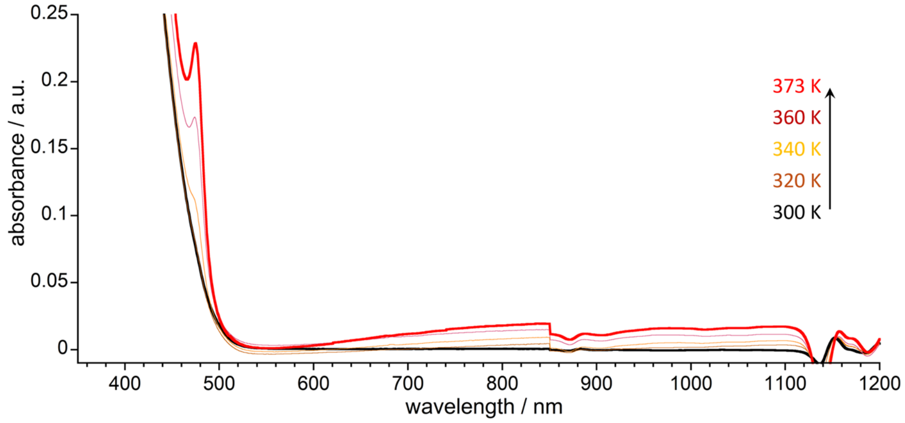

Figure S20. Variable-temperature electronic absorption spectra for **1b**$_2$ in toluene.

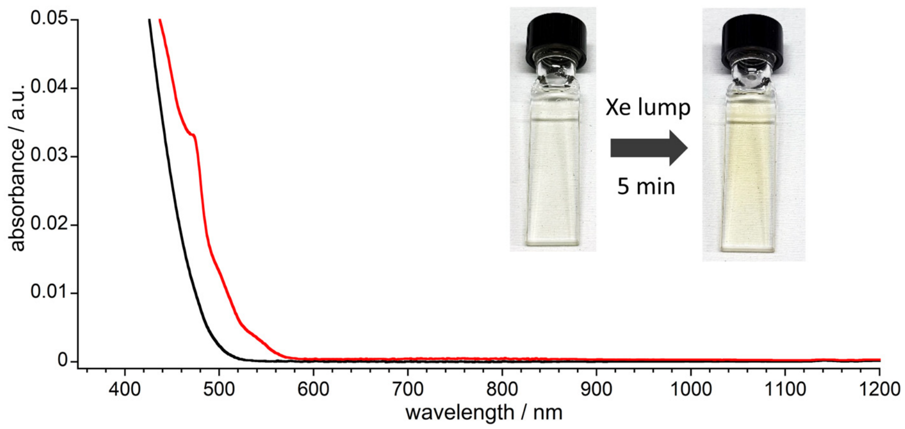

Figure S21. Electronic absorption spectral changes of **1b**$_2$ upon Xe-lamp irradiation in toluene: (black) before irradiation and (red) 5 min after irradiation. Inserts represent the photos for the measured solution before and after irradiation.

10. Computational method

10-1. General

All quantum chemical calculations were conducted using the Gaussian 16 Rev. C. 01 program.[5] The geometries of **1a**, **1a**$^+$, **1b**, **2a**, **2b**, and **1b**$_2$ were optimized with the (U)B3LYP-D3(BJ) functional and 6-311G(d) basis set. From the frequency analyses, these calculated geometries locate at the local minimum giving all positive vibrational frequencies. The obtained (U)B3LYP-D3(BJ) geometries were employed for the calculations of the other physical properties.

Symmetry-adapted molecular orbitals and spin density maps for **1a**, **1a**$^+$, **1b**, **2a**, **2b**, and **1b**$_2$ were evaluated at the (U)B3LYP/6-311+G(d,p) level. Electronic excitation properties for **1a**, **1a**$^+$, **1b**, **2a**, **2b**, and **1b**$_2$ were evaluated by the TD-DFT method with (U)B3LYP and 6-311+G(d,p) basis set.

NICS(1) values for **1a** and **1a**$^+$ were calculated at the GIAO-(U)B3LYP/6-311+G(d) method using the optimized structure. AICD plots for **1a** and **1a**$^+$ were calculated by using the method developed by Herges[6] and only π-electrons are considered at the CSGT-(U)B3LYP/6-311+G(d) level. The magnetic field is perpendicular to the molecular planes. Yellow surface is the isosurface of the induced current density under the magnetic field. Green allows with red head indicate the induced current density vectors. The clockwise and counterclockwise density vectors indicate diamagnetic and paramagnetic ring currents, respectively.

10-2. Molecular orbitals

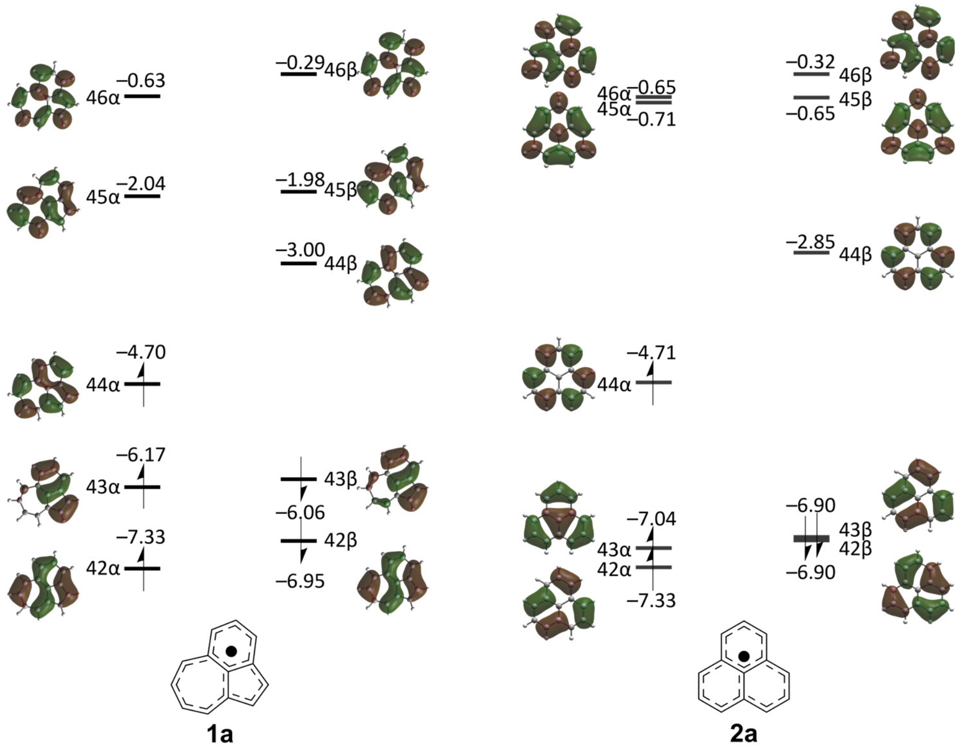

Figure S22. Calculated orbital energy diagram (/ eV) of selected molecular orbitals for **1a** and **2a** at the UB3LYP/6-311+G(d,p)//UB3LYP-D3(BJ)/6-311G(d) level. The orbital of ψ_{44} corresponds to the SOMO.

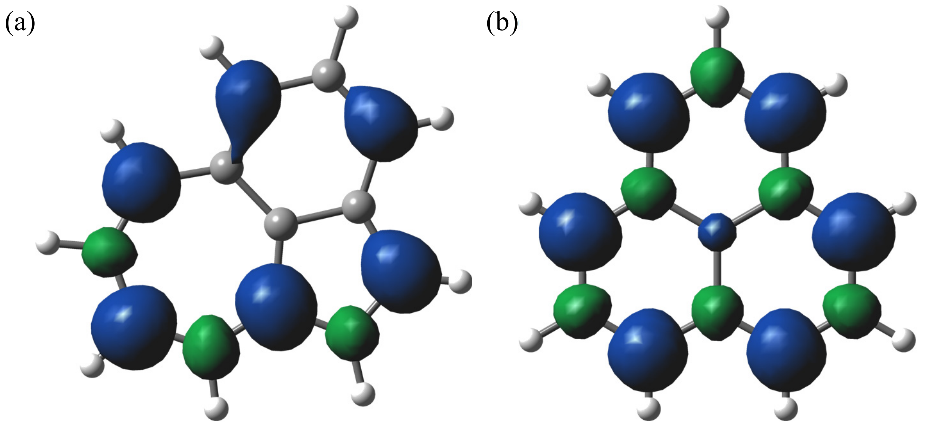

Figure S23. Spin density distribution for (a) **1a** and (b) **2a** at the UB3LYP/6-311+G(d,p)//UB3LYP-D3(BJ)/6-311G(d) level. Positive (blue) and negative (green) spin densities are shown.

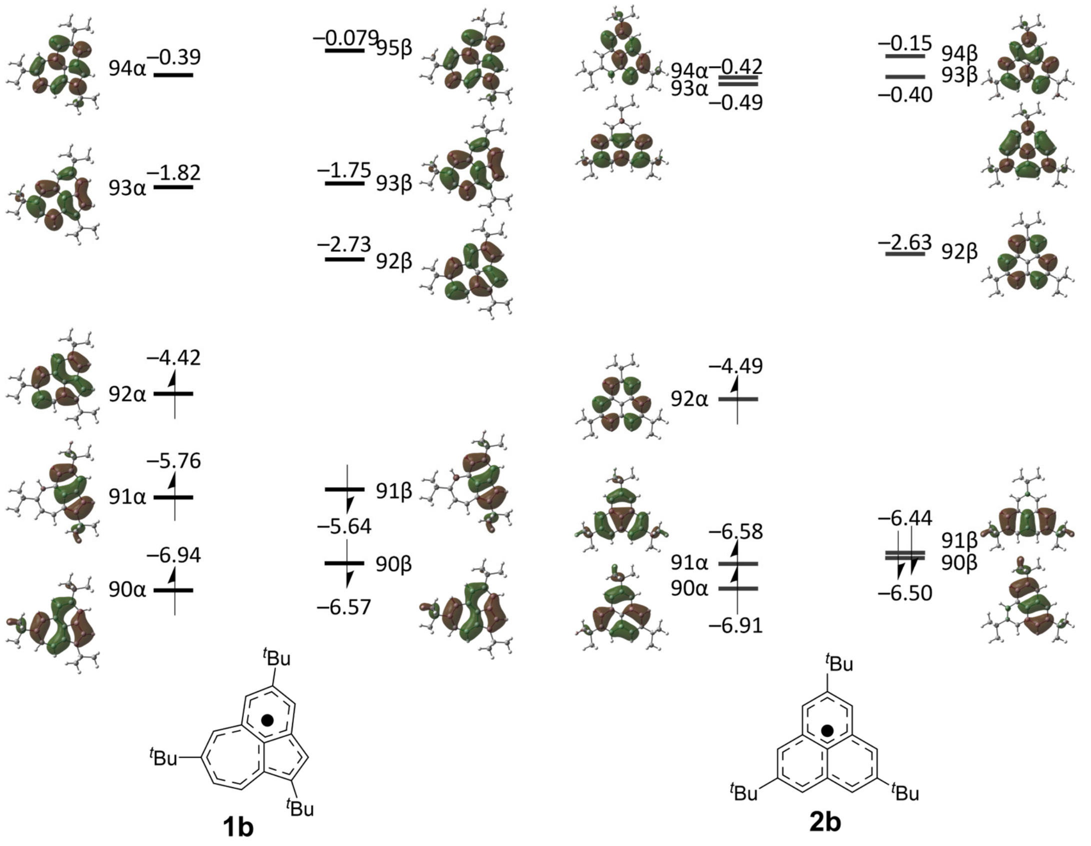

Figure S24. Calculated orbital energy diagram (/ eV) of selected molecular orbitals for **1b** and **2b** at the UB3LYP/6-311+G(d,p)//UB3LYP-D3(BJ)/6-311G(d) level. The orbital of ψ_{92} corresponds to the SOMO.

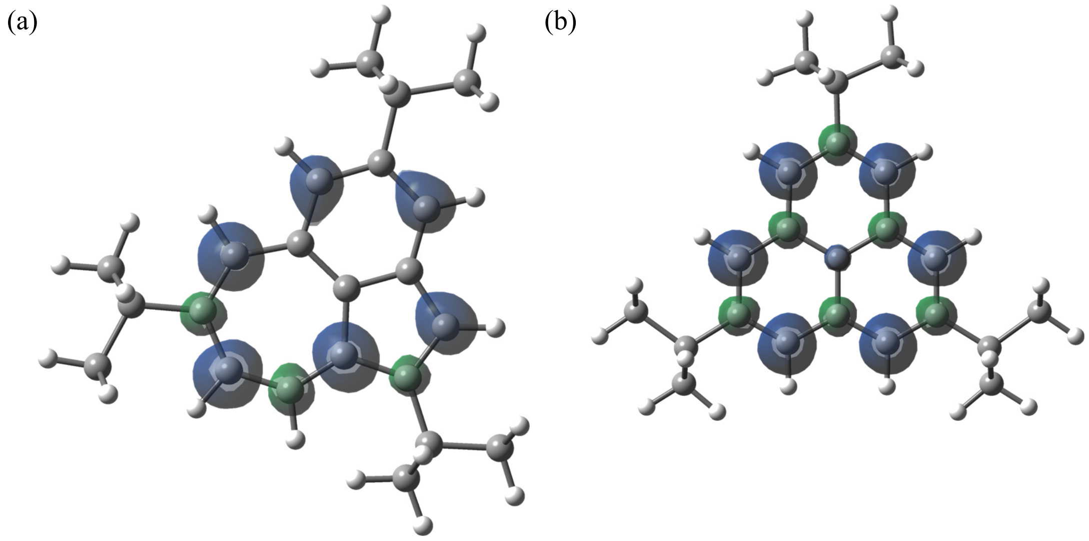

Figure S25. Spin density distribution for (a) **1b** and (b) **2b** at the UB3LYP/6-311+G(d,p)//UB3LYP-D3(BJ)/6-311G(d) level. Positive (blue) and negative (green) spin densities are shown.

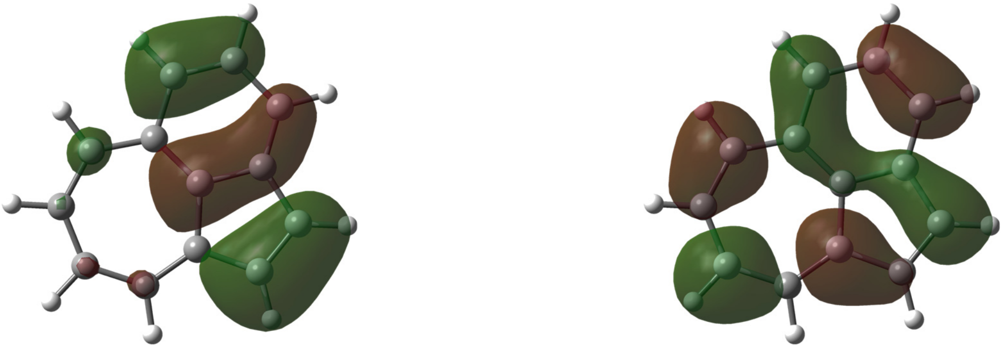

HOMO (−10.7 eV) LUMO (−7.84 eV)

Figure S26. Frontier molecular orbitals for **1a**$^{+}$ at the RB3LYP/6-311+G(d,p)//RB3LYP-D3(BJ)/6-311G(d) level.

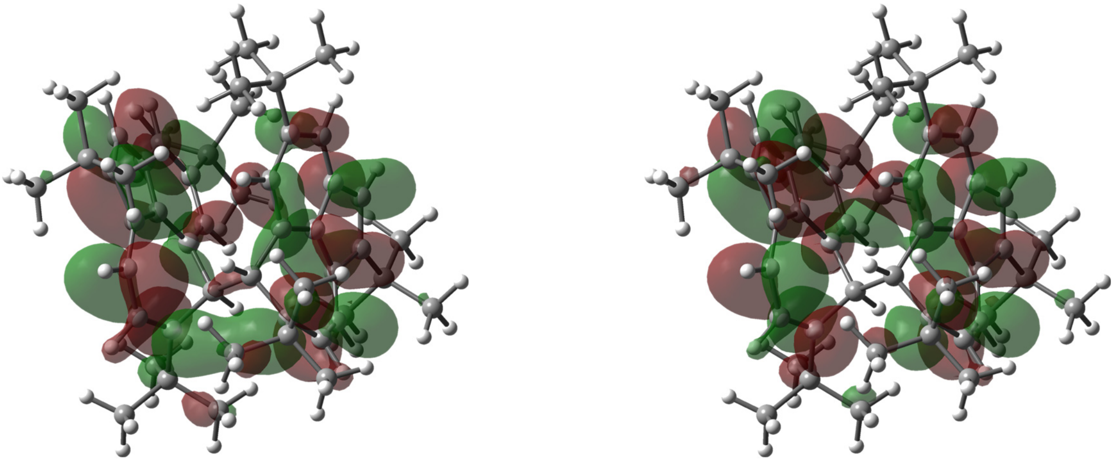

LUMO+1 (−1.95 eV) LUMO (−1.97 eV)

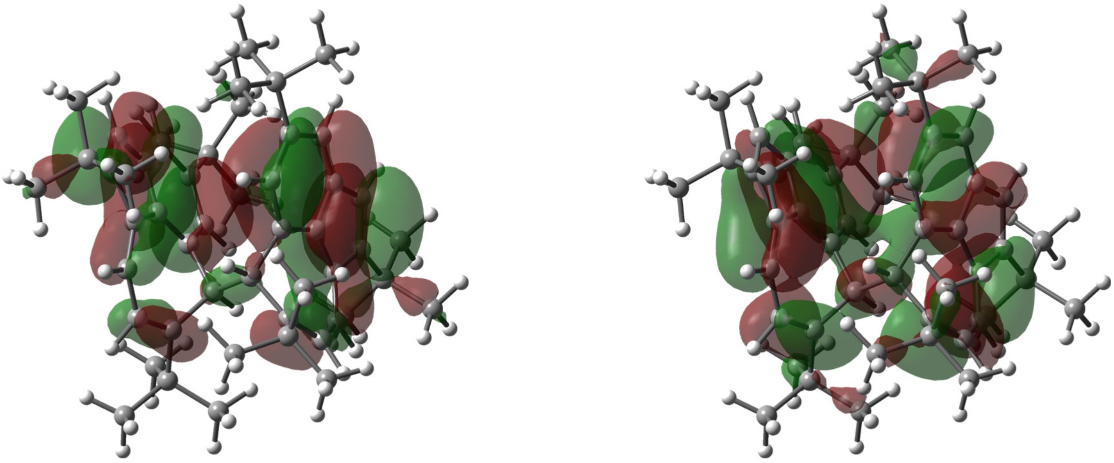

HOMO (−5.50 eV) HOMO−1 (−5.69 eV)

Figure S27. Frontier molecular orbitals for **1b**$_2$ at the RB3LYP/6-311+G(d,p)//RB3LYP-D3(BJ)/6-311G(d) level.

10-3. TD-DFT calculations

Table S6. Selected excitation energies of **1a** calculated at the TD-UB3LYP/6-311+G(d,p)//UB3LYP-D3(BJ)/6-311G(d) level.

Excited state number	Excitation energy / eV (wavelength / nm)	Excitation amplitudes		Oscillator strength
1	1.51 (822)	0.984	(44α – 45α)	0.0018
		−0.113	(42β – 44β)	
2	1.91 (649)	0.123	(44α – 46α)	0.0031
		0.971	(43β – 44β)	
3	2.81 (442)	−0.143	(42α – 45α)	0.0048
		−0.588	(43α – 45α)	
		−0.188	(44α – 46α)	
		0.118	(41β – 44β)	
		0.380	(41β – 44β)	
		0.120	(42β – 45β)	
		−0.103	(43β – 44β)	
		0.621	(43β – 45β)	
4	2.98 (417)	0.109	(44α – 45α)	0.0132
		0.384	(44α – 46α)	
		−0.145	(44α – 50α)	
		−0.189	(41β – 44β)	
		0.810	(41β – 44β)	
		−0.297	(43β – 45β)	
5	3.02 (410)	−0.286	(43α – 45α)	0.0198
		0.733	(44α – 46α)	
		−0.390	(41β – 44β)	
		0.189	(41β – 45β)	
		−0.304	(42β – 44β)	
		0.143	(42β – 45β)	
		−0.139	(43β – 44β)	
		0.179	(43β – 45β)	

Table S7. Selected excitation energies of **2a** calculated at the TD-UB3LYP/6-311+G(d,p)//UB3LYP-D3(BJ)/6-311G(d) level.

Excited state number	Excitation energy / eV (wavelength / nm)	Excitation amplitudes		Oscillator strength
1	2.71 (458)	0.696	($44\alpha - 46\alpha$)	0.0002
		0.711	($42\beta - 44\beta$)	
2	2.71 (458)	−0.696	($44\alpha - 47\alpha$)	0.0002
		0.711	($43\beta - 44\beta$)	
3	2.89 (429)	0.852	($44\alpha - 45\alpha$)	0.0000
		−0.513	($41\beta - 44\beta$)	
4	3.15 (393)	0.512	($44\alpha - 45\alpha$)	0.0000
		0.852	($41\beta - 44\beta$)	
5	3.67 (338)	0.141	($43\alpha - 46\alpha$)	0.175
		0.696	($44\alpha - 47\alpha$)	
		−0.156	($42\beta - 45\beta$)	
		0.676	($43\beta - 44\beta$)	
6	3.67 (338)	−0.141	($43\alpha - 47\alpha$)	0.174
		0.696	($44\alpha - 46\alpha$)	
		−0.676	($42\beta - 44\beta$)	
		−0.156	($43\beta - 45\beta$)	

Table S8. Selected excitation energies of **1b** calculated at the TD-UB3LYP/6-311+G(d,p)//UB3LYP-D3(BJ)/6-311G(d) level.

Excited state number	Excitation energy / eV (wavelength / nm)	Excitation amplitudes		Oscillator strength
1	1.46 (848)	0.983	(92α – 93α)	0.0022
		−0.123	(90β – 92β)	
2	1.79 (691)	−0.106	(91α – 94α)	0.0024
		0.971	(91β – 92β)	
		−0.106	(91β – 93β)	
3	2.69 (461)	−0.116	(90α – 93α)	0.0043
		−0.613	(91α – 93α)	
		0.113	(92α – 94α)	
		−0.310	(90β – 92β)	
		0.136	(91β – 92β)	
		0.666	(91β – 93β)	
4	2.88 (430)	−0.136	(91α – 93α)	0.0261
		0.121	(92α – 93α)	
		0.172	(92α – 94α)	
		0.113	(92α –100α)	
		0.104	(89β – 93β)	
		0.886	(90β – 92β)	
		0.291	(91β – 93β)	
5	2.96 (419)	0.221	(91α – 93α)	0.0164
		0.807	(92α – 94α)	
		−0.470	(89β – 92β)	
		−0.166	(89β – 93β)	
		−0.109	(90β – 92β)	
		−0.103	(91β – 92β)	

Table S9. Selected excitation energies of **2b** calculated at the TD-UB3LYP/6-311+G(d,p)//UB3LYP-D3(BJ)/6-311G(d) level.

Excited state number	Excitation energy / eV (wavelength / nm)	Excitation amplitudes		Oscillator strength
1	2.60 (477)	−0.534	(92α – 93α)	0.0023
		−0.333	(92α – 94α)	
		−0.401	(90β – 92β)	
		0.651	(91β – 92β)	
2	2.69 (461)	−0.124	(92α – 93α)	0.0026
		0.300	(92α – 94α)	
		0.518	(92α – 95α)	
		0.583	(89β – 92β)	
		0.468	(90β – 92β)	
		0.226	(91β – 92β)	
3	2.80 (443)	−0.160	(92α – 93α)	0.0001
		0.296	(92α – 94α)	
		−0.291	(92α – 95α)	
		−0.538	(89β – 92β)	
		0.548	(90β – 92β)	
		0.446	(91β – 92β)	
4	3.08 (402)	−0.584	(92α – 93α)	0.0012
		0.624	(92α – 94α)	
		−0.264	(92α – 95α)	
		0.127	(89β – 92β)	
		−0.264	(90β – 92β)	
		−0.319	(91β – 92β)	
5	3.54 (350)	0.130	(91α – 95α)	0.199
		0.548	(92α – 93α)	
		0.515	(92α – 94α)	
		−0.119	(89β – 93β)	
		−0.425	(90β – 92β)	
		0.436	(91β – 92β)	
6	3.63 (341)	−0.127	(91α – 93α)	0.200
		0.191	(92α – 94α)	
		0.731	(92α – 95α)	
		−0.554	(89β – 92β)	
		−0.209	(90β – 92β)	

Table S10. Selected excitation energies of **1a**$^{+}$ calculated at the TD-RB3LYP/6-311+G(d,p)//RB3LYP-D3(BJ)/6-311G(d) level.

Excited state number	Excitation energy / eV (wavelength / nm)	Excitation amplitudes		Oscillator strength
1	2.06 (602)	0.701	(HOMO – LUMO)	0.0068
2	3.25 (381)	−0.123	(HOMO−2 – LUMO)	0.0015
		−0.159	(HOMO−1 – LUMO)	
		0.672	(HOMO – LUMO+1)	
3	3.39 (365)	−0.162	(HOMO−2 – LUMO+1)	0.0628
		0.654	(HOMO−1 – LUMO)	
		0.144	(HOMO – LUMO+1)	
		−0.109	(HOMO – LUMO+2)	
4	3.88 (320)	0.571	(HOMO−2 – LUMO)	0.0434
		0.372	(HOMO−1 – LUMO+1)	
		0.135	(HOMO – LUMO+1)	

Table S11. Selected excitation energies of **1b**$_2$ calculated at the TD-RB3LYP/6-311+G(d,p)//RB3LYP-D3(BJ)/6-311G(d) level.

Excited state number	Excitation energy / eV (wavelength / nm)	Excitation amplitudes		Oscillator strength
1	2.86 (434)	−0.135	(HOMO−3 – LUMO+1)	0.0112
		−0.133	(HOMO−2 – LUMO+1)	
		0.163	(HOMO−1 – LUMO)	
		0.659	(HOMO – LUMO)	
2	2.82 (430)	−0.160	(HOMO−3 – LUMO)	0.0051
		−0.115	(HOMO−2 – LUMO)	
		0.208	(HOMO−1 – LUMO+1)	
		0.644	(HOMO – LUMO+1)	
3	3.13 (396)	−0.367	(HOMO−2 – LUMO+1)	0.0059
		0.557	(HOMO−1 – LUMO)	
		−0.223	(HOMO – LUMO)	
4	3.14 (395)	−0.447	(HOMO−2 – LUMO)	0.0087
		0.486	(HOMO−1 – LUMO+1)	
		−0.244	(HOMO – LUMO+1)	
5	3.28 (378)	0.156	(HOMO−3 – LUMO+1)	0.0043
		0.557	(HOMO−2 – LUMO+1)	
		0.395	(HOMO−1 – LUMO)	
6	3.30 (376)	0.398	(HOMO−3 – LUMO)	0.0662
		0.407	(HOMO−2 – LUMO)	
		0.412	(HOMO−1 – LUMO+1)	

10-4. NICS(1) and ACID calculations

Table S12. Summary the NICS(1) values and ACID plots for **1a**, **1a**$^{+}$, and **2a**.

<table>
<tr><th>comp.</th><th>NICS(1) / ppm</th><th>ACID</th></tr>
<tr><td>1a</td><td>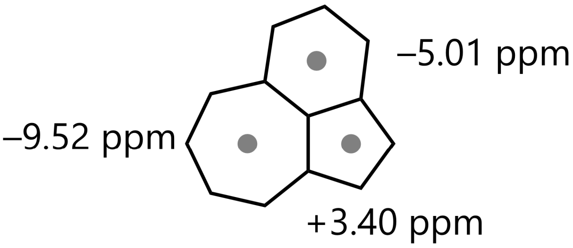

1a^{+}</td><td>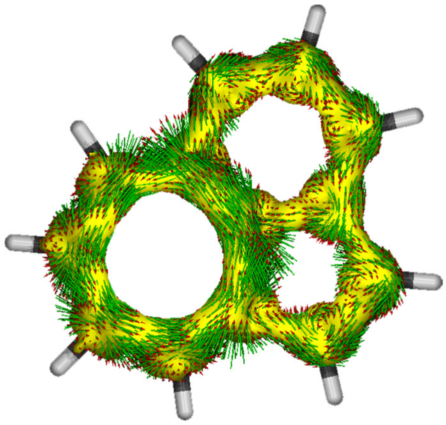</td></tr>
<tr><td>1a^{+}</td><td>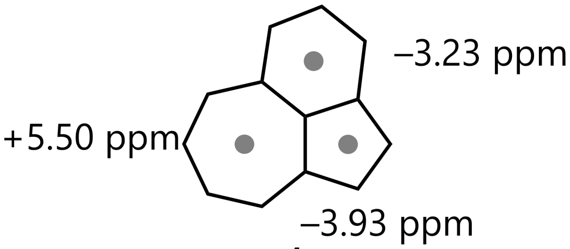

1a</td><td>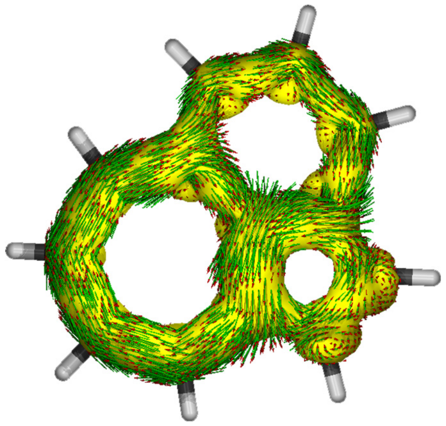</td></tr>
<tr><td>2a</td><td>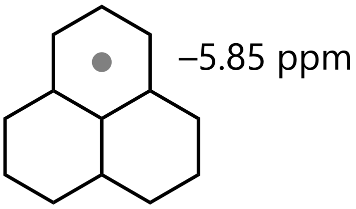

2a</td><td>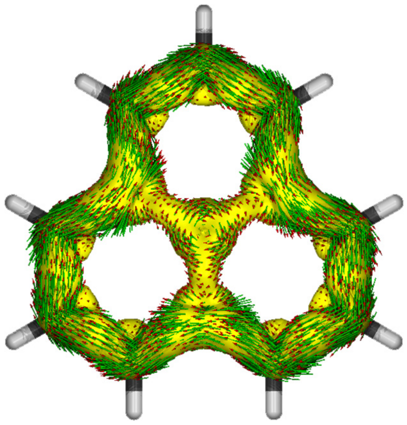</td></tr>
</table>

The calculations were performed at the (U)B3LYP/6-311+G(d)//(U)B3LYP-D3(BJ)/6-311G(d) level.

10-5. Radical stabilization energies (RSEs)

Thermodynamic stability of neutral radicals (R•) can be estimated by a radical stabilization energy (RSE) that is evaluated with a second-order restricted open-shell Møller–Plesset theory (ROMP2) calculation for isodesmic hydrogen-transfer reactions defined as below equation;

$$CH_3\bullet \; + \; R\text{–}H \xrightarrow[]{\Delta H \; (= \text{RSEs})} CH_4 \; + \; R\bullet$$

The geometries of the concerned radicals and their corresponding monohydro compounds were optimized using the (U)B3LYP-D3(BJ) functional and the 6-311G(d) basis set. From the frequency analyses, these calculated geometries are located at the local minimum, giving all positive vibrational frequencies. By using the optimized geometries, the energies were estimated at the ROMP2/6-31G(d,p) level.

Table S13. Total energies of monohydro compounds of **1b** calculated with RB3LYP-D3(BJ)/6-311G(d).

Monohydro compounds	Total energy / Hartree	Relative energy / kcal·mol^{-1}
2*H*	−973.4545219	3.44
3*H*	−973.4414151	11.7
5*H*	−973.4403547	12.3
**6*H***	**−973.4600001**	**0.00**
8*H*	−973.4558187	2.62
9a*H*	−973.4520067	5.02

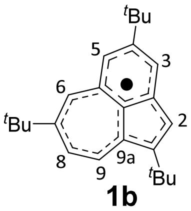

1b

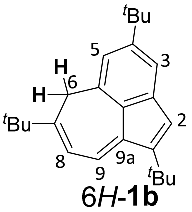

6*H*-**1b**

Table S14. Total energies of radicals and the corresponding monohydro compounds, calculated with ROMP2/6-31G(d,p).

Compounds	Total energy / Hartree	RSEs of radicals / kcal·mol^{-1}
$CH_3\bullet$	−39.6925479	0.00
CH_4	−40.3645630	
**2b**	−969.4677678	−47.3
1*H*-**2b**	−970.0643327	
**1b**	−969.4115014	−37.7
6*H*-**1b**	−970.0233666	

10-6. Cartesian coordinates for the optimized geometries

Table S15. Optimized geometries for **1a** in the singlet state optimized at the UB3LYP-D3(BJ)/6-311G(d) level.

Atom	X	Y	Z	Atom	X	Y	Z
C	0.00000000	0.29488800	0.00000000	H	1.88560700	3.16202800	0.00000000
C	0.31183300	1.67866200	0.00000000	C	-2.21328300	-0.99272900	0.00000000
C	1.01150000	-0.67674300	0.00000000	H	3.15378500	-0.94311600	0.00000000
C	-0.94114200	2.41391700	0.00000000	C	-0.41963400	-2.77899500	0.00000000
C	1.64085000	2.10478200	0.00000000	H	1.66511300	-2.71369300	0.00000000
C	-1.45606900	0.17730800	0.00000000	H	-3.03434100	1.75688600	0.00000000
C	2.35034500	-0.21366500	0.00000000	H	3.69350300	1.45580800	0.00000000
C	0.76771700	-2.10042300	0.00000000	C	-1.75978000	-2.30257100	0.00000000
C	-1.97889900	1.51683800	0.00000000	H	-3.29319200	-0.85944400	0.00000000
H	-1.02493400	3.49306600	0.00000000	H	-0.33038800	-3.86263700	0.00000000
C	2.65480400	1.14277600	0.00000000	H	-2.52459600	-3.07316000	0.00000000

Table S16. Optimized geometries for **1a**$^{+}$ in the singlet state optimized at the RB3LYP-D3(BJ)/6-311G(d) level.

Atom	X	Y	Z	Atom	X	Y	Z
C	0.00000000	0.28768300	0.00000000	H	1.96283300	3.08698600	0.00000000
C	0.35821800	1.66152900	0.00000000	C	-2.23668700	-0.91082400	0.00000000
C	0.98216100	-0.71903800	0.00000000	H	3.13545700	-1.04669100	0.00000000
C	-0.88355200	2.44747400	0.00000000	C	-0.50615600	-2.76355600	0.00000000
C	1.67951800	2.04019100	0.00000000	H	1.59086600	-2.74869800	0.00000000
C	-1.44028700	0.22365200	0.00000000	H	-2.98585100	1.85904700	0.00000000
C	2.35794200	-0.29183000	0.00000000	H	3.73130800	1.33338700	0.00000000
C	0.71582700	-2.10539900	0.00000000	C	-1.80384900	-2.23900600	0.00000000
C	-1.93782600	1.59524300	0.00000000	H	-3.31121500	-0.75573400	0.00000000
H	-0.92855600	3.52804400	0.00000000	H	-0.44894900	-3.84712900	0.00000000
C	2.68969400	1.03599400	0.00000000	H	-2.59590500	-2.98189200	0.00000000

Table S17. Optimized geometries for **1b** in the singlet state optimized at the UB3LYP-D3(BJ)/6-311G(d) level.

Atom	X	Y	Z	Atom	X	Y	Z
C	0.48867000	-0.14614500	0.00000000	C	3.49816500	-3.88497200	0.00000000
C	-0.43035200	0.91213500	0.00000000	C	1.41368000	-4.36055300	1.26655900
C	1.87477100	0.11209300	0.00000000	H	1.77454100	4.18184500	2.16654900
C	0.10310000	2.22407100	0.00000000	H	1.74746500	5.71443900	1.27973300
C	-1.86418900	0.76788200	0.00000000	H	0.32241100	4.67248000	1.29409500
C	0.30500800	-1.59224300	0.00000000	H	3.92310300	3.61288400	-0.88544600
C	2.36645700	1.41816400	0.00000000	H	3.76963400	5.12885600	0.00000000
C	2.55519700	-1.16857300	0.00000000	H	3.92310300	3.61288400	0.88544600
C	1.47341500	2.49962500	0.00000000	H	1.77454100	4.18184500	-2.16654900
H	-0.60304700	3.04716500	0.00000000	H	1.74746500	5.71443900	-1.27973300
C	-2.66732400	-0.35077700	0.00000000	H	0.32241100	4.67248000	-1.29409500
H	-2.38030300	1.72208700	0.00000000	H	-4.30850800	0.16938200	-2.16765500
C	-0.92711000	-2.23380600	0.00000000	H	-5.65643700	0.90157300	-1.28511300
C	1.63839300	-2.19308400	0.00000000	H	-4.07815300	1.68778200	-1.30218100
H	3.43646900	1.58280800	0.00000000	H	-4.87320700	-1.97953000	0.88636100
H	3.63063800	-1.27767700	0.00000000	H	-6.10697000	-1.09257700	0.00000000
C	1.94782300	3.95932400	0.00000000	H	-4.87320700	-1.97953000	-0.88636100
C	-4.19822200	-0.08400300	0.00000000	H	-4.30850800	0.16938200	2.16765500
C	-2.21439500	-1.69946300	0.00000000	H	-5.65643700	0.90157300	1.28511300
H	-0.90708900	-3.31704800	0.00000000	H	-4.07815300	1.68778200	1.30218100
C	1.97386200	-3.67765200	0.00000000	H	1.82388600	-3.89408300	-2.16560400
C	1.41368000	4.67259400	1.25907900	H	1.68691700	-5.42014800	-1.27939800
C	3.47942800	4.07518800	0.00000000	H	0.32731000	-4.29825400	-1.33306500
C	1.41368000	4.67259400	-1.25907900	H	3.96391800	-3.44488200	0.88521100
C	-4.57812700	0.71882400	-1.26208300	H	3.72915300	-4.95338100	0.00000000
C	-5.04894900	-1.36512800	0.00000000	H	3.96391800	-3.44488200	-0.88521100
C	-4.57812700	0.71882400	1.26208300	H	1.82388600	-3.89408300	2.16560400
H	-2.98824900	-2.45340600	0.00000000	H	1.68691700	-5.42014800	1.27939800
C	1.41368000	-4.36055300	-1.26655900	H	0.32731000	-4.29825400	1.33306500

Table S18. Optimized geometries for **1b**$_2$ in the singlet state optimized at the RB3LYP-D3(BJ)/6-311G(d) level.

Atom	X	Y	Z	Atom	X	Y	Z
C	1.35731600	-0.30270000	0.14142200	H	1.88673200	-2.39042500	3.61864600
C	1.28971800	-1.62094400	-0.27583700	H	-2.09293300	1.27214200	-3.57103500
C	-1.28971800	1.62094400	-0.27583700	H	0.94192700	1.18296500	1.58003800
C	2.07134300	0.33864900	-2.11724800	H	-1.21025900	3.90674200	-2.72102400
C	-1.35731600	0.30270000	0.14142200	H	-4.08631100	-0.15319300	2.19219900
C	-0.78197000	3.90189500	-0.58412200	H	-4.12234100	-1.51879200	3.32259100
C	0.30923800	-5.32464900	-0.34269400	H	-3.06112300	-1.55997600	1.91398300
C	-0.78230700	-0.11323500	1.47636700	H	-1.61955000	-3.13122900	-2.13315600
C	-2.07134300	-0.33864900	-2.11724800	H	-2.89444400	-3.52108200	-3.28820700
C	1.50555500	-0.60642200	2.60178700	H	-3.31773900	-2.84415400	-1.71623300
C	0.91749900	-2.83121200	0.44435800	H	-0.98006600	-4.83001800	1.37403500
C	1.91744000	-1.00148700	-2.53548200	H	-1.39925800	-6.33677200	0.55587900

C	-1.78501400	-0.65867900	-0.79176900	H	-1.80019400	-4.78179700	-0.18387700
C	1.51968300	-1.96442700	-1.62797200	H	-2.31416700	6.13350800	-0.04764300
C	2.49281300	1.39888300	-3.14171800	H	-1.02349900	7.14693100	0.61715400
C	-1.51968300	1.96442700	-1.62797200	H	-1.52750400	5.68226200	1.46141900
C	-2.49281300	-1.39888300	-3.14171800	H	2.31416700	-6.13350800	-0.04764300
C	0.78197000	-3.90189500	-0.58412200	H	1.02349900	-7.14693100	0.61715400
C	-1.50555500	0.60642200	2.60178700	H	1.52750400	-5.68226200	1.46141900
C	0.97143500	-2.95232900	1.79970700	H	-0.61520500	-5.54614000	-2.31453300
C	-1.45653400	1.95358900	2.72435800	H	-0.26443600	-7.07140000	-1.49501400
C	-0.97143500	2.95232900	1.79970700	H	1.04600500	-6.15205800	-2.23286300
C	1.78501400	0.65867900	-0.79176900	H	0.98006600	4.83001800	1.37403500
C	-0.30923800	5.32464900	-0.34269400	H	1.39925800	6.33677200	0.55587900
C	1.17624400	-3.37800700	-1.77830200	H	1.80019400	4.78179700	-0.18387700
C	-2.36451200	-0.24891800	3.54119600	H	-1.38264500	-0.49160700	-4.80707100
C	1.45653400	-1.95358900	2.72435800	H	-1.73251500	-2.20911100	-5.01815200
C	-1.91744000	1.00148700	-2.53548200	H	-0.46704800	-1.68609400	-3.89050000
C	-0.91749900	2.83121200	0.44435800	H	0.61520500	5.54614000	-2.31453300
C	0.78230700	0.11323500	1.47636700	H	0.26443600	7.07140000	-1.49501400
C	-1.17624400	3.37800700	-1.77830200	H	-1.04600500	6.15205800	-2.23286300
C	2.36451200	0.24891800	3.54119600	H	4.08631100	0.15319300	2.19219900
C	-3.47310900	-0.90939100	2.68701000	H	4.12234100	1.51879200	3.32259100
C	-2.58353100	-2.80231500	-2.52522600	H	3.06112300	1.55997600	1.91398300
C	-1.04449400	-5.31390800	0.39919500	H	2.32185400	-1.05754800	5.30170600
C	-1.35513500	6.11377400	0.47549300	H	3.66233000	0.08423100	5.26297300
C	1.35513500	-6.11377400	0.47549300	H	3.70535300	-1.34395500	4.23227400
C	0.10891700	-6.06054400	-1.67828800	H	-2.32185400	1.05754800	5.30170600
C	1.04449400	5.31390800	0.39919500	H	-3.66233000	-0.08423100	5.26297300
C	-1.45653400	-1.44668100	-4.28320500	H	-3.70535300	1.34395500	4.23227400
C	-0.10891700	6.06054400	-1.67828800	H	-1.08862500	-2.04096500	3.50965300
C	3.47310900	0.90939100	2.68701000	H	-2.18861900	-1.94430600	4.88349600
C	3.04858400	-0.57404100	4.64326500	H	-0.73555800	-0.93738400	4.83155300
C	-3.04858400	0.57404100	4.64326500	H	-4.62808500	-0.99300400	-2.92331400
C	-1.54116900	-1.35724200	4.22584000	H	-4.19462500	-1.77302600	-4.45316700
C	-3.87576700	-1.03029100	-3.71548400	H	-3.86485700	-0.05580300	-4.20838000
C	2.58353100	2.80231500	-2.52522600	H	1.61955000	3.13122900	-2.13315600
C	1.54116900	1.35724200	4.22584000	H	2.89444400	3.52108200	-3.28820700
C	1.45653400	1.44668100	-4.28320500	H	3.31773900	2.84415400	-1.71623300
C	3.87576700	1.03029100	-3.71548400	H	1.08862500	2.04096500	3.50965300
H	-0.94192700	-1.18296500	1.58003800	H	2.18861900	1.94430600	4.88349600
H	2.09293300	-1.27214200	-3.57103500	H	0.73555800	0.93738400	4.83155300
H	-1.81957000	-1.69014400	-0.46753900	H	1.38264500	0.49160700	-4.80707100
H	0.80103200	-3.92886800	2.23821400	H	1.73251500	2.20911100	-5.01815200
H	-1.88673200	2.39042500	3.61864600	H	0.46704800	1.68609400	-3.89050000
H	-0.80103200	3.92886800	2.23821400	H	4.62808500	0.99300400	-2.92331400
H	1.81957000	1.69014400	-0.46753900	H	4.19462500	1.77302600	-4.45316700
H	1.21025900	-3.90674200	-2.72102400	H	3.86485700	0.05580300	-4.20838000

Table S19. Optimized geometries for **2a** in the singlet state optimized at the UB3LYP-D3(BJ)/6-311G(d) level.

Atom	X	Y	Z	Atom	X	Y	Z
C	0.00000000	0.00000000	0.00000000	H	-1.24296200	3.19587600	0.00000000
C	0.00000000	1.42809400	0.00000000	C	-1.20778500	-2.12938000	0.00000000
C	1.23676600	-0.71404700	0.00000000	C	-2.44798900	0.01871800	0.00000000
C	1.24020400	2.11066200	0.00000000	C	0.00000000	-2.81607700	0.00000000
C	-1.24020400	2.11066200	0.00000000	H	2.14622900	-2.67437500	0.00000000
C	-1.23676600	-0.71404700	0.00000000	H	3.38919100	-0.52150200	0.00000000
C	1.20778500	-2.12938000	0.00000000	H	3.37855500	1.95061000	0.00000000
C	2.44798900	0.01871800	0.00000000	H	-3.37855500	1.95061000	0.00000000
C	2.43879500	1.40803900	0.00000000	H	-2.14622900	-2.67437500	0.00000000
H	1.24296200	3.19587600	0.00000000	H	-3.38919100	-0.52150200	0.00000000
C	-2.43879500	1.40803900	0.00000000	H	0.00000000	-3.90121900	0.00000000

Table S20. Optimized geometries for **2b** in the singlet state optimized at the UB3LYP-D3(BJ)/6-311G(d) level.

Atom	X	Y	Z	Atom	X	Y	Z
C	-0.02065200	-0.01161200	0.00000000	H	-4.17468000	4.62302200	0.00000000
C	0.69125100	1.22009500	0.00000000	H	-4.05550300	3.10415700	-0.88552400
C	-1.43901100	-0.01104700	0.00000000	H	-4.05550300	3.10415700	0.88552400
C	-0.04377500	2.42646800	0.00000000	C	4.35660800	0.02585200	0.00000000
C	2.10436100	1.18595700	0.00000000	C	4.84669900	0.76938900	-1.25914700
C	0.68981100	-1.24175700	0.00000000	C	4.84669900	0.76938900	1.25914700
C	-2.12288000	-1.25133100	0.00000000	C	4.98169100	-1.37697000	0.00000000
C	-2.12264800	1.23029200	0.00000000	H	4.50704800	0.26362600	-2.16660100
C	-1.44324200	2.44717200	0.00000000	H	4.47831700	1.79658800	-1.29475800
H	0.51214300	3.35788500	0.00000000	H	5.93994300	0.80728800	-1.27975600
C	2.82182600	-0.01637300	0.00000000	H	4.50704800	0.26362600	2.16660100
H	2.63336400	2.13289500	0.00000000	H	5.93994300	0.80728800	1.27975600
C	-0.04397200	-2.44681800	0.00000000	H	4.47831700	1.79658800	1.29475800
C	2.10833600	-1.21282900	0.00000000	H	6.07147200	-1.29480500	0.00000000
C	-1.44393900	-2.46808500	0.00000000	H	4.69623400	-1.95046700	0.88554900
H	-3.20527200	-1.22687400	0.00000000	H	4.69623400	-1.95046700	-0.88554900
H	-3.20500400	1.20615200	0.00000000	C	-2.17408200	-3.81858100	0.00000000
H	0.51198600	-3.37824900	0.00000000	C	-1.77460500	-4.61467900	-1.25905300
H	2.62845700	-2.16228400	0.00000000	C	-1.77460500	-4.61467900	1.25905300
C	-2.17381500	3.79755700	0.00000000	C	-3.70159000	-3.65934600	0.00000000

C	-1.77460500	4.59373100	1.25907600	H	-2.04343100	-4.06813700	-2.16660900
C	-1.77460500	4.59373100	-1.25907600	H	-0.70058600	-4.80802300	-1.29464500
C	-3.70130600	3.63798500	0.00000000	H	-2.28729500	-5.58101300	-1.27944200
H	-2.04314000	4.04701700	2.16661300	H	-2.04343100	-4.06813700	2.16660900
H	-0.70066800	4.78752500	1.29464800	H	-2.28729500	-5.58101300	1.27944200
H	-2.28771800	5.55983500	1.27955600	H	-0.70058600	-4.80802300	1.29464500
H	-2.04314000	4.04701700	-2.16661300	H	-4.17480700	-4.64446300	0.00000000
H	-2.28771800	5.55983500	-1.27955600	H	-4.05587000	-3.12556100	0.88552100
H	-0.70066800	4.78752500	-1.29464800	H	-4.05587000	-3.12556100	-0.88552100

Table S21. Optimized geometries for CH_3• in the singlet state optimized at the UB3LYP-D3(BJ)/6-311G(d) level.

Atom	X	Y	Z	Atom	X	Y	Z
C	0.00000000	-0.00000100	0.00000000	H	0.77631400	-0.75195500	0.00000000
H	-1.03937500	-0.29631900	0.00000000	H	0.26306200	1.04828100	0.00000000

Table S22. Optimized geometries for CH_4 in the singlet state optimized at the RB3LYP-D3(BJ)/6-311G(d) level.

Atom	X	Y	Z	Atom	X	Y	Z
C	0.00000000	0.00000000	0.00000000	H	-0.62949000	0.62949000	-0.62949000
H	0.62949000	0.62949000	0.62949000	H	0.62949000	-0.62949000	-0.62949000
H	-0.62949000	-0.62949000	0.62949000				

Table S23. Optimized geometries for 2*H*-**1b** in the singlet state optimized at the RB3LYP-D3(BJ)/6-311G(d) level.

Atom	X	Y	Z	Atom	X	Y	Z
C	0.03135700	-0.53382100	-0.00000200	H	-3.58361900	-5.03641400	0.00000600
C	-1.41250600	-0.83068400	-0.00001400	H	-2.07762200	-4.80703600	-0.88436600
C	0.73402600	0.68426800	-0.00000100	H	-2.07762000	-4.80701100	0.88436600
C	-2.43086800	0.19770200	-0.00001300	C	4.38865400	-0.56174500	0.00003000
C	-1.60215200	-2.18651600	-0.00001700	C	4.88702200	0.17714500	-1.25881800
C	0.73448300	-1.74163000	0.00000900	C	4.88699600	0.17719400	1.25886000
C	0.14071000	2.01214400	-0.00000800	C	4.99985700	-1.97068400	0.00006300
C	2.13806000	0.60722800	0.00000900	H	4.54390800	-0.32579000	-2.16653600
C	-2.31129100	1.54600300	-0.00001100	H	4.52652000	1.20714900	-1.29469400
H	-3.45250500	-0.15952900	-0.00001100	H	5.98066400	0.20654900	-1.27858800
C	-0.25137700	-2.87529100	0.00000500	H	4.54385100	-0.32569800	2.16659000
C	2.12230600	-1.78625500	0.00001800	H	5.98063800	0.20658600	1.27865800
C	-1.14974500	2.42433000	-0.00001200	H	4.52650400	1.20720300	1.29468000
H	0.89274700	2.79539800	-0.00000100	H	6.09051000	-1.90056300	0.00006500
C	2.85465800	-0.59432800	0.00001700	H	4.70802700	-2.54125800	0.88553700
H	2.68532900	1.54411300	0.00001100	H	4.70803300	-2.54129600	-0.88538900
H	-3.26320800	2.05892300	-0.00000900	C	-1.38972700	3.96133200	-0.00000400
H	-0.12047600	-3.52435600	0.87476700	C	-0.75245100	4.58184100	1.26077900
H	-0.12045100	-3.52438200	-0.87473200	C	-0.75260400	4.58183900	-1.26086600
H	2.62365000	-2.74644400	0.00002500	C	-2.87665800	4.35658500	0.00008400
C	-2.90766100	-2.97441100	-0.00002800	H	-1.18533700	4.15041100	2.16704000
C	-3.73314800	-2.65954900	1.26718100	H	0.32550500	4.41627700	1.29925800
C	-3.73311300	-2.65959200	-1.26727100	H	-0.92346800	5.66247000	1.28435400
C	-2.63615300	-4.49153700	-0.00000400	H	-1.18558700	4.15039300	-2.16707300
H	-3.16580900	-2.91165400	2.16679300	H	-0.92364200	5.66246500	-1.28443100
H	-4.00456900	-1.60648600	1.33825500	H	0.32535000	4.41629500	-1.29946800
H	-4.65771600	-3.24542400	1.27582800	H	-2.95776800	5.44634700	0.00007600
H	-3.16572400	-2.91166500	-2.16685900	H	-3.40301800	3.99352700	-0.88614200
H	-4.65764700	-3.24551900	-1.27595400	H	-3.40290900	3.99354900	0.88638200
H	-4.00459500	-1.60654600	-1.33835700				

Table S24. Optimized geometries for 3*H*-**1b** in the singlet state optimized at the RB3LYP-D3(BJ)/6-311G(d) level.

Atom	X	Y	Z	Atom	X	Y	Z
C	0.01042000	-0.50968500	-0.03471700	H	6.07925400	-1.24566000	-1.09246300
C	0.66485600	0.73046200	-0.03032200	H	4.71483500	-2.35346100	-1.00077800
C	-1.43985300	-0.82193700	-0.00593800	H	4.64583700	-0.95983000	-2.08724100
C	0.07179500	1.99416400	-0.01727100	C	-1.52258600	3.91409400	-0.00130700
C	2.13318900	0.68156600	-0.02467400	C	-0.87585600	4.53285900	1.25687000
C	0.70330600	-1.71287600	-0.05665500	C	-0.89469100	4.53405000	-1.26840400
C	-2.44639000	0.12251700	0.01230600	C	-3.00953700	4.30328600	0.00986200
C	-1.55196600	-2.25045200	-0.00818400	H	-1.30417100	4.10079800	2.16484900
C	-1.27960700	2.38334300	-0.00406400	H	0.20275600	4.37320000	1.29132100
H	0.78812800	2.80809300	-0.01747100	H	-1.05167900	5.61227300	1.27954900
C	2.86968800	-0.44323100	-0.04391300	H	-1.33732500	4.10366100	-2.17029500
H	2.62676300	1.64518000	0.01092700	H	-1.06986500	5.61364600	-1.28688000
C	2.19418500	-1.79739200	-0.12017500	H	0.18306500	4.37331900	-1.31973900
C	-0.24842800	-2.76008100	-0.04024700	H	-3.09614400	5.39249700	0.00880400
C	-2.37817900	1.52499100	0.00970600	H	-3.53947600	3.93441800	-0.87153900
H	-3.46070800	-0.25965400	0.02877000	H	-3.52562400	3.93751700	0.90071900
H	2.58107200	-2.44881200	0.67407300	C	-2.82311200	-3.08915500	0.01867100
H	2.50136700	-2.28950900	-1.05317800	C	-3.63644200	-2.80745700	1.30064600
H	0.00777000	-3.81167000	-0.05097000	C	-3.68871300	-2.80863600	-1.22892300
H	-3.35097500	1.99593600	0.02200800	C	-2.47989800	-4.58907800	0.01223700
C	4.39801900	-0.43065000	0.02284000	H	-3.03682400	-3.02158700	2.18883400
C	4.98148000	0.98369700	-0.11354100	H	-3.96166400	-1.76904500	1.36930500

C	4.84841100	-1.00324300	1.38584000	H	-4.52992300	-3.43905800	1.33350000
C	4.98754200	-1.30194200	-1.10654100	H	-3.12629300	-3.02365500	-2.14091000
H	4.68617400	1.45336600	-1.05513400	H	-4.58279900	-3.44023900	-1.22428800
H	4.67062300	1.63558100	0.70620200	H	-4.01659200	-1.77030300	-1.28511900
H	6.07302900	0.93579500	-0.09331900	H	-3.39801500	-5.18213400	0.03255300
H	4.53051600	-2.03878200	1.52187500	H	-1.92324800	-4.87114700	-0.88509900
H	5.93937500	-0.98272800	1.46164900	H	-1.88467900	-4.86976200	0.88492100
H	4.43964100	-0.41353100	2.20994800				

Table S25. Optimized geometries for 5*H*-**1b** in the singlet state optimized at the RB3LYP-D3(BJ)/6-311G(d) level.

Atom	X	Y	Z	Atom	X	Y	Z
C	0.05754300	-0.48320900	-0.05844100	H	-2.80166100	-5.50454500	0.03868000
C	0.56824500	0.80127900	-0.07741000	H	-1.36442100	-5.03602700	-0.87146000
C	-1.34592100	-0.96267000	-0.02191900	H	-1.33889200	-5.02097600	0.89861400
C	2.08103600	0.94442600	-0.20172200	C	4.44318900	-0.09999900	0.03600900
C	-0.16084400	2.00185000	-0.03325000	C	4.93670900	0.79267700	-1.12266700
C	0.89283200	-1.61994900	-0.05871100	C	4.79213000	0.58279600	1.37774400
C	-2.45806600	-0.12891000	-0.00817000	C	5.20543500	-1.43141500	-0.03035600
C	-1.29579000	-2.38350100	-0.01256800	H	4.67474100	0.35815000	-2.09130700
C	2.93236700	-0.30241200	-0.05695700	H	4.51233700	1.79799700	-1.07740100
H	2.41737600	1.71346100	0.50153000	H	6.02450800	0.89769000	-1.08372300
H	2.27390000	1.37847500	-1.19267800	H	4.45479500	-0.02482100	2.22088000
C	-1.53467100	2.24200300	-0.00531700	H	5.87411100	0.71849400	1.46696900
H	0.46637400	2.88744400	-0.03334400	H	4.33238800	1.56949300	1.46940100
C	2.33131000	-1.50476200	-0.02553600	H	6.28176200	-1.24597700	0.01226800
C	0.06550400	-2.75385700	-0.03002400	H	4.95393100	-2.08617900	0.80732200
C	-2.54239400	1.26205300	-0.00120100	H	4.99550600	-1.96911100	-0.95832500
H	-3.42308700	-0.62235600	0.00473800	C	-1.94777500	3.73683000	0.02390000
H	2.90988800	-2.41831600	0.04875600	C	-1.35936700	4.40513200	1.28512500
H	0.43340500	-3.77117900	-0.01626500	C	-1.40858800	4.44223400	-1.23918900
H	-3.55901600	1.62794000	0.01640000	C	-3.46899400	3.95733200	0.05671700
C	-2.46464000	-3.36075600	0.01540400	H	-1.72250400	3.91219400	2.19046900
C	-3.30941000	-3.16356000	1.29270200	H	-0.26911500	4.36990900	1.30350400
C	-3.34971100	-3.18644300	-1.23768700	H	-1.65723600	5.45670900	1.32917400
C	-1.95537800	-4.81286400	0.02045100	H	-1.80629300	3.97516300	-2.14378400
H	-2.69350600	-3.30421000	2.18437100	H	-1.70829800	5.49420300	-1.24144100
H	-3.74912000	-2.16771100	1.35375800	H	-0.31979600	4.40909000	-1.30031600
H	-4.12679200	-3.89083900	1.32645300	H	-3.67582500	5.02997800	0.07764100
H	-2.76275200	-3.34422300	-2.14587200	H	-3.96525800	3.54861500	-0.82667300
H	-4.16813000	-3.91332400	-1.23189100	H	-3.93109100	3.52035000	0.94507300
H	-3.79074700	-2.19140500	-1.30306700				

Table S26. Optimized geometries for 6*H*-**1b** in the singlet state optimized at the RB3LYP-D3(BJ)/6-311G(d) level.

Atom	X	Y	Z	Atom	X	Y	Z
C	-0.29690500	-0.43162300	-0.23112100	H	4.60440800	-4.11125300	0.62291900
C	0.83067900	0.31070600	-0.54935200	H	3.10262600	-3.85156800	1.50928300
C	-1.70530800	-0.05215000	-0.20608600	H	3.05937700	-4.35991900	-0.18637300
C	0.66262600	1.67856700	-1.16269000	C	-3.97889100	-1.46779500	0.02901500
C	2.07774900	-0.30830600	-0.40556200	C	-4.56757200	-0.79300400	1.28829700
C	-0.19741200	-1.79835500	0.09077600	C	-4.60003400	-0.85098200	-1.24160600
C	-2.14894200	1.22923800	-0.08476300	C	-4.37303200	-2.95394800	0.07237700
C	-2.46747500	-1.31875900	-0.00071200	H	-4.13635900	-1.22881100	2.19271900
C	-0.06166500	2.65047200	-0.25230600	H	-4.37668400	0.27965000	1.31716500
H	0.07891700	1.56532600	-2.08524700	H	-5.65149000	-0.93933700	1.32364300
H	1.63532300	2.06796700	-1.46042900	H	-4.20803700	-1.33950800	-2.13731700
C	2.21171500	-1.65125700	-0.02413500	H	-5.68647500	-0.97979100	-1.23383100
H	2.96555100	0.26969900	-0.64008600	H	-4.39038100	0.21500000	-1.33199500
C	1.04687400	-2.40483300	0.20334500	H	-5.46158200	-3.05193500	0.05345300
C	-1.56131900	-2.31486300	0.20384800	H	-3.97162400	-3.49908400	-0.78540600
C	-1.34214300	2.41766700	0.10887300	H	-4.01558700	-3.44034900	0.98328400
H	-3.21242000	1.38849600	0.05115900	C	0.70816700	3.89270500	0.19281500
H	1.11015000	-3.45466900	0.46036700	C	1.98342600	3.44171000	0.94028100
H	-1.79488000	-3.35089700	0.40759400	C	1.10477300	4.73150200	-1.04187600
H	-1.85967500	3.19182700	0.66526400	C	-0.09940600	4.79274100	1.13880100
C	3.61700200	-2.25813900	0.09522000	H	1.72775200	2.84891400	1.82143700
C	4.33003700	-2.18405000	-1.27070700	H	2.63849200	2.83530600	0.31260400
C	4.42814400	-1.46457100	1.14011900	H	2.55555500	4.31427500	1.26836600
C	3.58407400	-3.72922300	0.53572900	H	0.21685500	5.07087800	-1.58147600
H	3.76937400	-2.73250300	-2.03195500	H	1.67091100	5.61489700	-0.73307600
H	4.43881400	-1.15427100	-1.61691100	H	1.72859300	4.17266800	-1.74207500
H	5.33130300	-2.62063000	-1.20485300	H	0.51163000	5.64481200	1.44711800
H	3.94204100	-1.50052000	2.11839400	H	-0.99545000	5.19238400	0.65734600
H	5.43375300	-1.88339200	1.24394300	H	-0.40431500	4.25981600	2.04247000
H	4.53256600	-0.41417600	0.86103800				

Table S27. Optimized geometries for 8*H*-**1b** in the singlet state optimized at the RB3LYP-D3(BJ)/6-311G(d) level.

Atom	X	Y	Z	Atom	X	Y	Z
C	0.19586100	-0.42125100	-0.21820100	H	-2.60342200	-5.44872400	0.61769900
C	-1.17993700	-0.92742900	-0.22311200	H	-1.12883400	-5.17657900	-0.31015700
C	0.69450900	0.88176200	-0.11442700	H	-1.17373300	-4.78549100	1.41583600
C	-2.24194800	-0.17932500	-0.57867300	C	4.48997300	0.19845600	0.02471000

C	-1.08728300	-2.39676200	0.02307900	C	4.90986600	1.02855800	-1.20557500
C	1.06211800	-1.52848200	-0.10293300	C	4.83463000	0.97740600	1.31053000
C	-0.15585000	2.05115600	0.11106200	C	5.30603100	-1.10284700	0.02259400
C	2.09610500	1.02189800	-0.03342100	H	4.67538400	0.49729400	-2.13157400
C	-2.08328100	1.19137400	-1.18381900	H	4.39718800	1.99199600	-1.23808300
H	-3.24586600	-0.58363700	-0.52993200	H	5.98660000	1.22308700	-1.18808500
C	0.23153800	-2.72533800	0.03185900	H	4.53811800	0.41294400	2.19829600
C	2.43690800	-1.35949500	-0.03871100	H	5.91143600	1.16297600	1.36919600
C	-1.43918700	2.20841200	-0.25296000	H	4.32913000	1.94440900	1.34824800
H	0.32114400	2.83198600	0.69371300	H	6.37347800	-0.86903800	0.04368100
C	2.97741400	-0.06059100	-0.03197800	H	5.08984700	-1.72207200	0.89694600
H	2.49191000	2.02880300	0.04992600	H	5.11758300	-1.69966100	-0.87347200
H	-3.05386100	1.54565200	-1.52720500	C	-2.29877800	3.39105700	0.19030700
H	-1.45571800	1.10026700	-2.07903600	C	-3.55737900	2.85096600	0.90390000
H	0.63530400	-3.72283200	0.14032900	C	-1.56514300	4.32303000	1.16533200
H	3.08004700	-2.22721800	0.03683700	C	-2.72142500	4.22342000	-1.03948500
C	-2.25453700	-3.35698400	0.15880200	H	-4.15950900	2.21364300	0.25265200
C	-3.18179600	-2.90035600	1.30481300	H	-3.28199000	2.26446800	1.78368200
C	-3.04853100	-3.43704800	-1.16376500	H	-4.19340600	3.67868700	1.23062100
C	-1.75408500	-4.77259400	0.48995300	H	-0.68631200	4.78239100	0.70614100
H	-2.63617700	-2.86827800	2.25122800	H	-2.23130400	5.13212400	1.47569900
H	-3.59587300	-1.90665300	1.13206500	H	-1.24347300	3.79354400	2.06518400
H	-4.01733100	-3.59745100	1.41858600	H	-3.34793200	5.06553700	-0.73154900
H	-2.40041500	-3.76303600	-1.98091800	H	-1.84495100	4.62456800	-1.55470100
H	-3.86635800	-4.15805900	-1.07053300	H	-3.29473800	3.63844700	-1.76160000
H	-3.48071400	-2.47805200	-1.44900600				

Table S28. Optimized geometries for 9a*H*-**1b** in the singlet state optimized at the RB3LYP-D3(BJ)/6-311G(d) level.

Atom	X	Y	Z	Atom	X	Y	Z
C	-0.16946200	-0.44906000	-0.11451000	H	-6.31155300	-1.05629400	0.24394500
C	1.27064500	-0.86864800	-0.20506000	H	-5.06014100	-1.95093400	-0.61584300
C	-0.98290700	-1.57458000	0.04750900	H	-4.97497200	-1.77022300	1.14375700
C	2.06301500	-0.12108600	0.84136000	C	2.12716700	3.55135800	-0.08141700
C	1.18601000	-2.38973300	-0.06624200	C	1.39252900	4.49920400	-1.04035300
H	1.66694100	-0.61015300	-1.19890600	C	2.26902700	4.27119200	1.27714400
C	-0.69690500	0.83231200	-0.20762400	C	3.52857000	3.27725600	-0.66585100
C	-2.36729400	-1.43986000	0.10289000	H	1.22610500	4.03814600	-2.01701400
C	-0.10868600	-2.74979900	0.06820400	H	0.42590800	4.81980500	-0.64421100
C	2.11228000	1.21625400	0.91857500	H	1.99198400	5.39976000	-1.19588900
H	2.60273000	-0.69911000	1.58236700	H	2.86105100	3.69873700	1.99375900
C	0.13825500	2.01849000	-0.35532500	H	2.76681500	5.23603900	1.14320000
C	-2.10146800	0.94122500	-0.16947900	H	1.28826300	4.45437700	1.72305700
C	-2.94381300	-0.16375600	-0.00776100	H	4.07767000	4.21386600	-0.79965100
H	-2.98774200	-2.32038700	0.21036600	H	4.12207800	2.63152500	-0.01581000
H	-0.47602800	-3.76507300	0.15539800	H	3.45290800	2.78614500	-1.63969100
C	1.39786100	2.20852400	0.10804200	C	2.39113100	-3.31317800	-0.12083000
H	2.76067500	1.62700700	1.68749200	C	3.50595700	-2.69127500	-0.98174800
H	-0.35757500	2.85313200	-0.83821900	C	2.93539400	-3.56254000	1.30448500
H	-2.53379500	1.93288900	-0.25343700	C	2.00015300	-4.67249100	-0.72809100
C	-4.46409400	0.05418900	0.04079500	H	3.16766200	-2.52408000	-2.00796200
C	-4.81282700	0.97258000	1.22980800	H	3.84372700	-1.73356800	-0.57907600
C	-4.93153500	0.71496200	-1.27192500	H	4.37097900	-3.35884000	-1.02034000
C	-5.23838400	-1.26118000	0.21311000	H	2.15842000	-3.98032300	1.94852000
H	-4.48603200	0.52784000	2.17324100	H	3.77064500	-4.26910100	1.27286500
H	-4.33442900	1.95002800	1.14175200	H	3.29981300	-2.64555400	1.77052800
H	-5.89368900	1.13420700	1.28591300	H	2.87949100	-5.31761900	-0.80997500
H	-4.69111900	0.08428300	-2.13167400	H	1.26832100	-5.19663300	-0.10910000
H	-6.01426100	0.87281000	-1.25778900	H	1.57217500	-4.55020700	-1.72582700
H	-4.45674900	1.68534500	-1.42999700				

Table S29. Optimized geometries for 1*H*-**2b** in the singlet state optimized at the RB3LYP-D3(BJ)/6-311G(d) level.

Atom	X	Y	Z	Atom	X	Y	Z
C	0.04122900	0.03156700	-0.06142800	H	-3.34909500	-5.10528100	-1.06220100
C	0.53110200	-1.29557700	-0.09365900	H	-2.71718700	-3.80621700	-2.08170900
C	-1.35983100	0.29436300	-0.05189800	H	-1.62735200	-4.74069400	-1.04914000
C	-0.43263200	-2.45502300	-0.21721900	C	-1.48308200	4.12480200	0.00819100
C	1.89137300	-1.51014600	-0.05656500	C	-2.34793500	4.33961700	-1.25103400
C	0.95403700	1.11701100	-0.01885700	C	-2.35607300	4.30622200	1.26713200
C	-2.28636100	-0.83078400	-0.02154300	C	-0.38808400	5.20064600	0.02617400
C	-1.80042300	1.60049100	-0.02893600	H	-1.75093800	4.21549200	-2.15810400
C	-1.89885600	-2.11492800	-0.06024300	H	-3.17816200	3.63222000	-1.30130300
H	-0.29174600	-2.91295400	-1.20574500	H	-2.77050100	5.34883500	-1.25704800
H	-0.14973300	-3.23962500	0.49307700	H	-1.76548600	4.15551800	2.17436500
C	2.82844000	-0.44695500	-0.00623200	H	-2.77652400	5.31585200	1.29842200
H	2.25268100	-2.53383600	-0.07503300	H	-3.18841100	3.59996500	1.29215700
C	0.44575300	2.44496200	0.00007900	H	-0.84506300	6.19344700	0.03513300
C	2.34352100	0.84452800	0.00477300	H	0.24439900	5.12216100	0.91402900
H	-3.33715400	-0.57663500	0.05771000	H	0.25367900	5.14191500	-0.85653500
C	-0.90630900	2.70502500	-0.00976100	C	4.32531300	-0.77575600	0.03098900
H	-2.87024800	1.78108400	-0.01749300	C	4.63074500	-1.62211200	1.28393300
H	1.16506900	3.25432600	0.02455600	C	4.70611000	-1.57422000	-1.23277700
H	3.02031300	1.68953600	0.03669000	C	5.19951900	0.48548000	0.08081200
C	-2.87352500	-3.28873300	0.03947800	H	4.36710800	-1.07764500	2.19421000

C	-2.66548900	-4.00788300	1.39117700	H	4.07453700	-2.56174400	1.28670900
C	-4.33921500	-2.83673900	-0.03717300	H	5.69616700	-1.86676000	1.33002100
C	-2.62064700	-4.29116200	-1.10638200	H	4.49533500	-0.99586500	-2.13593200
H	-1.65639600	-4.41340900	1.49072600	H	5.77304600	-1.81646600	-1.22516600
H	-2.83704000	-3.32343600	2.22542200	H	4.15393600	-2.51357100	-1.30387600
H	-3.36439000	-4.84388000	1.48760700	H	6.25504000	0.20302000	0.10656900
H	-4.54916400	-2.30665200	-0.96955000	H	5.05020800	1.11859400	-0.79745100
H	-4.99801300	-3.70763500	0.00717600	H	4.99787200	1.08498900	0.97207700
H	-4.60886200	-2.18281500	0.79543300				

Table S30. Optimized geometries for **1b**$_2$ in the first excited singlet state (S_1) optimized at the TD-RB3LYP-D3(BJ)/6-311G(d) level.

Atom	X	Y	Z	Atom	X	Y	Z
C	-0.31129600	0.17278700	1.34303200	H	-2.45371700	3.63198200	1.79571000
C	-1.62132400	-0.26541400	1.24572500	H	1.16974800	-3.66608300	-1.96673900
C	1.63991200	-0.30720200	-1.20721100	H	1.16600400	1.63003600	0.95318600
C	0.34991000	-2.08724200	2.05313000	H	3.81744200	-2.84650400	-1.11699000
C	0.31578400	0.13608200	-1.32204900	H	0.12997500	2.31805200	-4.12481300
C	3.85499800	-0.63474800	-0.74211300	H	-1.26293500	3.41375300	-4.20822900
C	-5.32558700	-0.37701700	0.27317900	H	-1.33130600	1.96367700	-3.20382000
C	-0.07460300	1.50346500	-0.80548800	H	-3.16633400	-2.03843400	-1.65360300
C	-0.35629500	-2.16110700	-2.01124000	H	-3.56700100	-3.15487600	-2.95769100
C	-0.65049400	2.62941600	1.47029800	H	-2.80017400	-1.61371200	-3.33459400
C	-2.83744600	0.43811500	0.86289500	H	-4.87010100	1.34772200	-1.02138100
C	-0.97651000	-2.52458000	1.87157500	H	-6.37468100	0.51406000	-1.41518500
C	-0.63839900	-0.77832800	-1.72807000	H	-4.81977200	-0.20713500	-1.84571300
C	-1.94834600	-1.62682500	1.46295300	H	6.01401900	-0.23863200	-2.39027300
C	1.41374400	-3.08803400	2.52006400	H	7.10306800	0.44005900	-1.17084100
C	1.94287300	-1.72938300	-1.39802200	H	5.62990400	1.30437700	-1.63290700
C	-1.45636300	-3.12223300	-2.48171200	H	-6.10816700	-0.09462800	2.29097400
C	-3.89490500	-0.60141700	0.72994500	H	-7.14827900	0.55889700	1.01602500
C	0.69099800	2.62751200	-1.47389400	H	-5.68687800	1.42085700	1.49981300
C	-2.97556400	1.79289700	0.90323400	H	-5.53780100	-2.35136200	-0.64989400
C	2.05858400	2.73375600	-1.33777000	H	-7.06632500	-1.54782400	-0.27936500
C	3.00478900	1.79248300	-0.88818900	H	-6.12346500	-2.27672800	1.01895600
C	0.65528900	-0.75035500	1.77889400	H	4.91047800	1.31218100	0.93408100
C	5.29991100	-0.44782900	-0.33485500	H	6.42883500	0.47497000	1.27961500
C	-3.35562000	-1.79343000	1.11931600	H	4.88869300	-0.22191600	1.79780900
C	-0.10737300	3.58692000	-2.35826400	H	-0.67265400	-4.84825700	-1.37284600
C	-1.99816100	2.73602400	1.38963400	H	-2.38438900	-4.96962100	-1.78978400
C	0.92364800	-2.62144800	-1.81235200	H	-1.85709800	-3.89360300	-0.48295500
C	2.84367200	0.38063500	-0.85434000	H	5.51159300	-2.40506500	0.62904800
C	0.10070800	1.50915600	0.77313200	H	7.03729800	-1.63037200	0.19655500
C	3.28557000	-1.90740400	-1.09647200	H	6.05137300	-2.37822600	-1.05717300
C	0.16914400	3.58806500	2.34248000	H	0.06822500	2.23257800	4.06097600
C	-0.67737300	2.76973600	-3.54397600	H	1.40416700	3.39619200	4.12666600
C	-2.82297200	-2.43292400	-2.61099800	H	1.50422800	1.99199200	3.06453800
C	-5.34416400	0.36796100	-1.07889000	H	-1.16992200	5.32325300	2.27732500
C	6.05240500	0.31767600	-1.45072600	H	-0.05494000	5.30266200	3.63952800
C	-6.10885900	0.43007000	1.33262100	H	-1.46453100	4.24523400	3.65302800
C	-6.04915900	-1.72070800	0.08124500	H	1.17317100	5.35419300	-2.15687500
C	5.38191800	0.33297700	0.99621000	H	0.14500200	5.35095900	-3.58828000
C	-1.59863700	-4.27819800	-1.47053500	H	1.58234600	4.32983900	-3.54147700
C	6.00875600	-1.79897900	-0.13194900	H	-1.99524500	3.51108300	-1.22627100
C	0.82498500	2.74653500	3.46402500	H	-1.83209100	4.91035500	-2.28447900
C	-0.68937800	4.67183900	3.01223500	H	-0.93595100	4.83430300	-0.76080400
C	0.75605400	4.71638800	-2.94097800	H	-0.95594800	-2.89033400	-4.59663200
C	-1.28593800	4.24384800	-1.60983100	H	-1.84195900	-4.37738800	-4.22302200
C	-1.06920900	-3.69175900	-3.86206300	H	-0.12642400	-4.24123000	-3.82252500
C	2.81078100	-2.45540800	2.59840000	H	3.13270800	-2.08067000	1.62511000
C	1.27882700	4.29415600	1.54037100	H	3.53785600	-3.20376100	2.92487800
C	1.48234100	-4.27402200	1.53670300	H	2.84495900	-1.63011900	3.31428000
C	1.03282400	-3.60534600	3.92229000	H	1.97989500	3.59269100	1.09207400
H	-1.13668700	1.62699400	-1.00317600	H	1.84318700	4.95762400	2.20237000
H	-1.23685400	-3.56354200	2.04127200	H	0.86039200	4.89804100	0.73294600
H	-1.66432500	-0.43951500	-1.77838800	H	0.52894800	-4.80094900	1.46363500
H	-3.95627200	2.21576700	0.71856400	H	2.23614500	-4.99631500	1.86437800
H	2.50282400	3.68095200	-1.62870800	H	1.74911600	-3.92708900	0.53773900
H	4.00046700	2.18500300	-0.73078900	H	0.97623400	-2.78179700	4.63872100
H	1.67862900	-0.40741300	1.84246700	H	1.77940800	-4.31966900	4.28266400
H	-3.87458100	-2.74136300	1.15304000	H	0.06376500	-4.10916200	3.91867100

11. Dimerization from 1b to $1b_2$

11-1. General

For the reaction profiles of the dimerization of **1b** to **$1b_2$** under thermal conditions, the (U)B3LYP-D3(BJ)/6-311G(d) level were employed. All molecular geometries were fully optimized at the singlet states and were confirmed to have all positive vibrational frequencies for local minimum states or an imaginary vibrational frequency for transition states at the same theoretical level. By using the obtained optimized structures, Gibbs free energies including contribution of vibrational entropy at an appropriate temperature were described in energy profiles, in which solvation effect was introduced using the SMD model (toluene).

11-2. Summary of each reaction step

Table S31. Summary for the energy for each reaction step

state	Total energy / Hartree[a]	Zero-point energy / Hartree[b]	Thermal correlation for Gibbs energy / Hartree[b]	Total energy +ZPE / Hartree	Gibus energy (298 K) / Hartree	imaginary frequency / cm^{-1} [b]	$<S^2>$ [b]
**1b**	−972.8622377	0.512609	0.458873	−972.3496287	−972.4033647	n.d.	0.782186
**1b** × 2	−1945.724475	1.025218	0.917746	−1944.699257	−1944.806729	n.d.	
**IM1**	−1945.748947	1.027485	0.945788	−1944.721462	−1944.803159	n.d.	1.02038
**TS1**	−1945.731073	1.027832	0.948828	−1944.703241	−1944.782245	303.2706*i*	0.384734
**$1b_2$**	−1945.775939	1.03269	0.954845	−1944.743249	−1944.821094	n.d.	n.d.

[a] (U)B3LYP-D3(BJ)/6-311G(d)/SMD (toluene)//(U)B3LYP-D3(BJ)/6-311G(d)

[b] (U)B3LYP-D3(BJ)/6-311G(d)

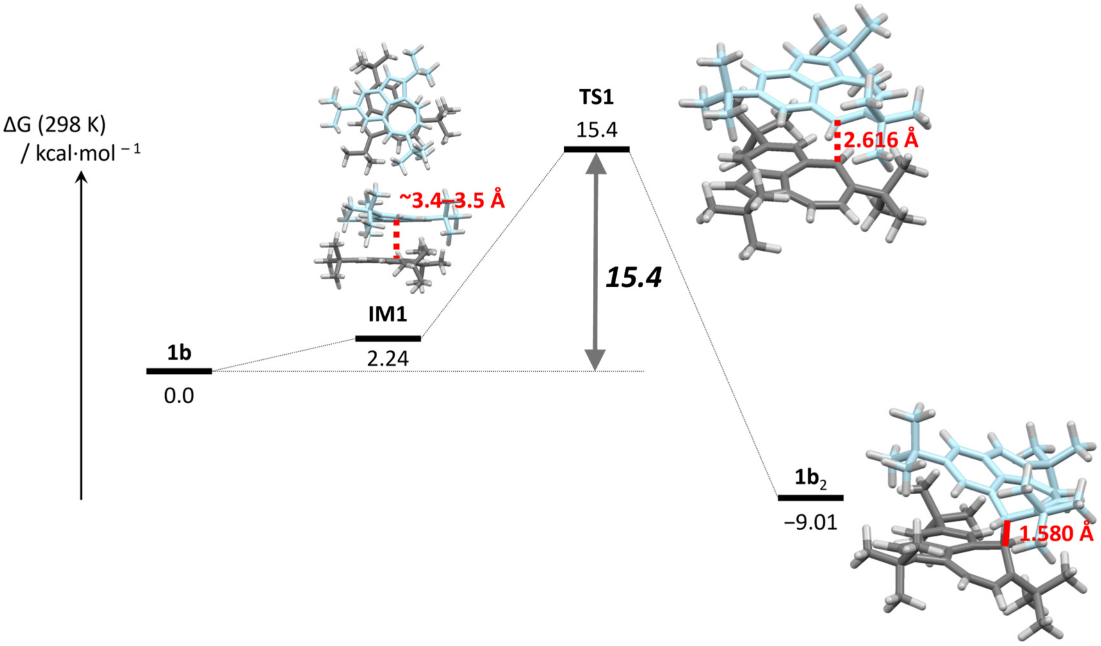

Figure S28. Energy diagram for the dimerization from **1b** to **$1b_2$**. The Gibbs-free-energy values relative to **1b** were calculated at the (U)B3LYP-D3(BJ)/6-311G(d)/SMD (toluene)//(U)B3LYP-D3(BJ)/6-311G(d). The intermediate **IM1** corresponds to the π-dimer of two molecules of **1b**, but the energy of **IM1** is endothermic relative to that of the monomeric **1b**. No observation of the π-dimer supports this estimation.

11-3. Cartesian coordinates for the optimized geometry for each reaction step

11-3-1. Cartesian coordinates for the optimized geometry for IM1

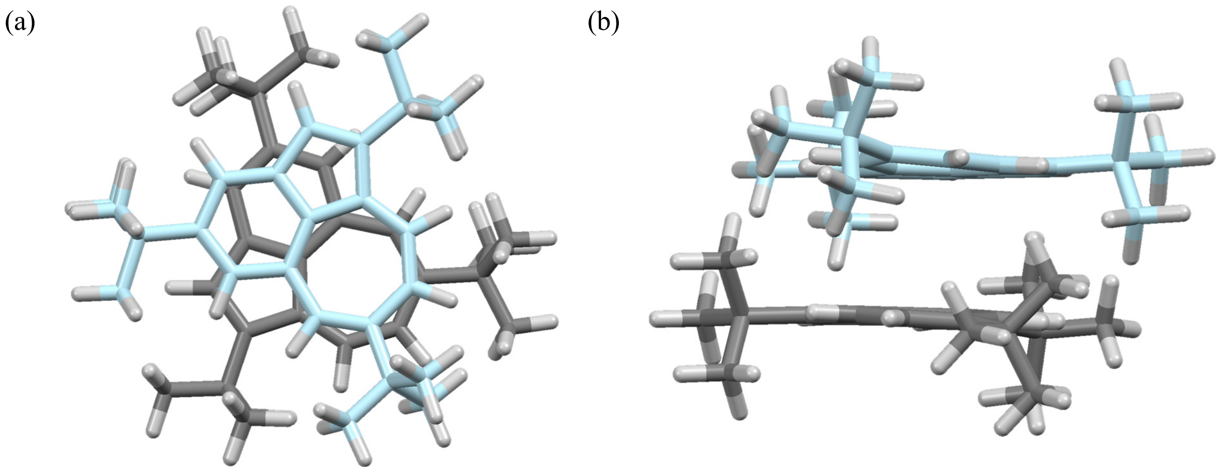

Figure S29. Optimized molecular structure of **IM1** shown in capped stick model. (a) Top and (b) side views.

Table S32. Optimized geometries for **IM1** optimized at the UB3LYP-D3(BJ)/6-311G(d) level.

Atom	X	Y	Z	Atom	X	Y	Z
C	-0.14598800	0.88182700	1.72476200	H	-3.94576200	-0.95134800	1.74829200
C	0.19240800	-0.47549900	1.68321100	H	3.90958100	0.49330600	-1.57511400
C	0.49240800	0.47828200	-1.65856600	H	-1.51027300	2.48924600	1.83454800
C	2.26492200	1.43128700	1.84271500	H	2.61447900	3.05924900	-1.65445700
C	0.24647700	-0.89804500	-1.69620200	H	-4.50515800	-0.34702700	-3.59165200
C	0.43736000	2.82336500	-1.70125800	H	-4.95479300	-2.01190800	-3.95811200
C	-0.08960700	-4.29659500	1.69664800	H	-3.38008400	-1.38377200	-4.46721100
C	-1.04854000	-1.51428400	-1.86153500	H	2.91095500	-3.95571900	-0.73051200
C	2.68780500	-1.29461700	-1.61859800	H	4.37945600	-4.34928800	-1.62599200
C	-2.70714000	0.77054600	1.82649400	H	2.89113700	-3.99340700	-2.49912700
C	-0.62754800	-1.68024800	1.64959300	H	-1.81812100	-4.12490700	0.34752700
C	2.57029600	0.05856900	1.79653000	H	-1.14389500	-5.75077600	0.45827900
C	1.37735800	-1.75737400	-1.65823900	H	-0.30189000	-4.49814500	-0.46392800
C	1.54638700	-0.87759300	1.71335700	H	-0.22922100	4.23190300	-3.97231600
C	3.39811000	2.44911700	2.02734600	H	-1.12682300	5.54118300	-3.18854700
C	1.81971800	0.96636000	-1.61278200	H	-1.73743800	3.88679600	-3.13464700
C	3.89704400	-2.23782400	-1.65742500	H	-0.34402300	-4.35667400	3.86165800
C	0.28518700	-2.82161500	1.66400000	H	-1.14837000	-5.68231100	3.00722100
C	-2.29564400	-0.95946900	-1.98875900	H	-1.85220800	-4.06433200	3.00433600
C	-2.01509900	-1.69041700	1.65394300	H	1.79850300	-5.02267500	0.85481900
C	-2.60571300	0.43123300	-1.86276700	H	0.88860200	-6.23221900	1.75952000
C	-1.79090600	1.54627600	-1.74649000	H	1.77544600	-4.97475700	2.62321400
C	0.92800300	1.81050000	1.78755600	H	-1.77280600	4.05176400	-0.49401600
C	-0.02185900	4.27136600	-1.80161200	H	-1.18367700	5.70694300	-0.64010600
C	1.56724900	-2.32722500	1.69696500	H	-0.30134300	4.53157700	0.34522500
C	-3.50439800	-1.86305900	-2.33848200	H	5.16751600	-0.93778100	-0.42356300
C	-2.91495200	-0.63143000	1.73652100	H	5.69928200	-2.61837700	-0.49369000
C	2.90144300	0.09620700	-1.59237200	H	4.29765700	-2.17158000	0.48745600
C	-0.40064600	1.62723800	-1.70243500	H	1.79772100	5.14517000	-0.94636400
C	-1.48608200	1.40656000	1.77740100	H	0.84252500	6.25734200	-1.92546200
C	1.74911300	2.41289300	-1.64537500	H	1.82498500	5.01924800	-2.71097900
C	-3.94981500	1.67871700	2.04599800	H	-3.74788800	1.61529100	4.22103500
C	-4.12214000	-1.36592300	-3.66510700	H	-4.69020500	2.99062400	3.62451900
C	3.48778600	-3.71710000	-1.62631000	H	-2.92938500	2.99134500	3.48325000
C	-0.88934400	-4.68677600	0.43764200	H	-5.46404700	0.40853400	1.08378000
C	-0.83124600	4.49129500	-3.09807800	H	-6.10348600	1.62342200	2.18550900
C	-0.91208500	-4.61451800	2.96460300	H	-5.34832300	0.17535200	2.83614500
C	1.17118500	-5.17654600	1.73558900	H	-4.17263700	-2.21065500	-0.29208500
C	-0.87363800	4.65940300	-0.57664800	H	-5.44368800	-2.39584700	-1.50845000
C	4.81805700	-1.97149200	-0.45042400	H	-4.90716300	-0.78798600	-1.02309800
C	1.18621800	5.22254700	-1.84816400	H	-2.40610900	-3.46892800	-3.35139900
C	-3.81809200	2.36061500	3.42471900	H	-4.02283000	-3.90646400	-2.80678500
C	-5.28504900	0.91533200	2.03512800	H	-2.71387800	-3.78905500	-1.63853800
C	-4.56801100	-1.80523300	-1.22427700	H	4.05419100	-2.15712500	-3.83469500
C	-3.12776700	-3.33839500	-2.54156900	H	5.54945200	-2.65137100	-3.02289600
C	4.68507900	-1.98303600	-2.95946400	H	5.05224500	-0.95603900	-3.01483900
C	2.90691900	3.89898600	1.90698600	H	2.43097500	4.07996800	0.94110100
C	-4.03685700	2.75641600	0.94944500	H	3.75189300	4.58634000	1.99840000
C	4.50113100	2.23281700	0.97341800	H	2.19277600	4.15575100	2.69313000
C	4.00231500	2.25508100	3.43424800	H	-3.16517300	3.40936100	0.94288300
H	-0.98841800	-2.59105600	-1.93891900	H	-4.91627400	3.38683400	1.10875600
H	3.59965600	-0.27499200	1.84533200	H	-4.12256800	2.29920000	-0.03828100

H	1.18441800	-2.82117800	-1.68067600	H	4.92405500	1.22794900	1.02079700
H	-2.49116500	-2.66293700	1.60923200	H	5.31886900	2.94206300	1.13142200
H	-3.66219000	0.66904700	-1.90034500	H	4.11123800	2.38347200	-0.03345900
H	-2.33077600	2.48430800	-1.70796900	H	3.24217300	2.39946900	4.20609600
H	0.66658400	2.85935900	1.81411500	H	4.80909100	2.97368100	3.60992300
H	2.47185200	-2.91648700	1.73112700	H	4.41389700	1.25089600	3.55831300

11-3-2. Cartesian coordinates for the optimized geometry for TS1

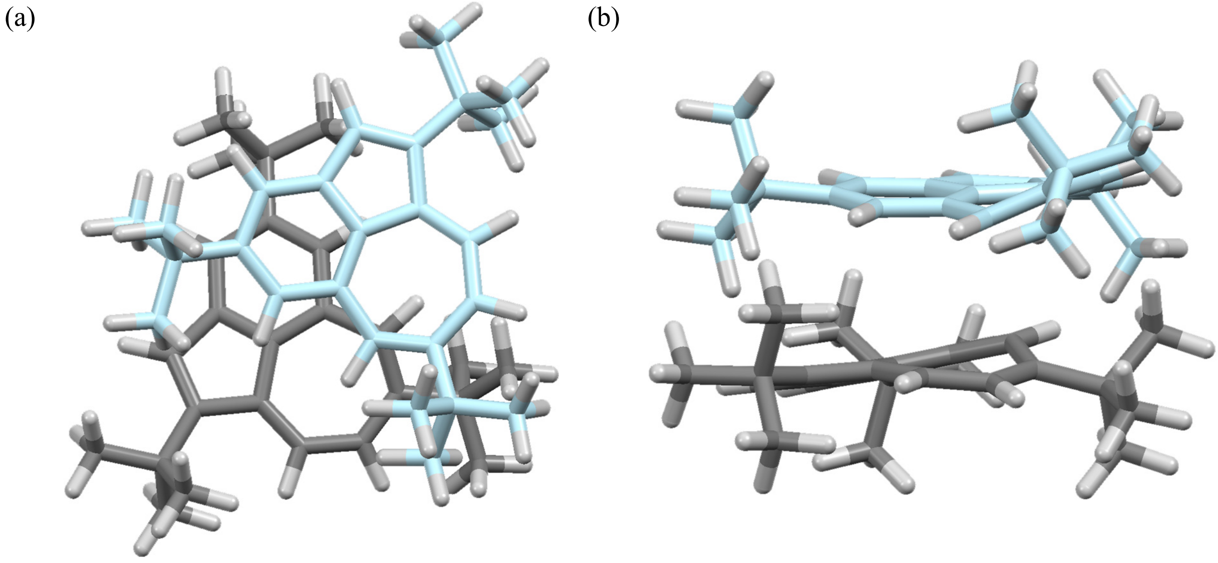

Figure S30. Optimized molecular structure of **TS1** shown in capped stick model. (a) Top and (b) side views.

Table S33. Optimized geometries for **TS1** optimized at the RB3LYP-D3(BJ)/6-311G(d) level.

Atom	X	Y	Z	Atom	X	Y	Z
C	0.47427900	0.10668300	-1.40902000	H	2.70995900	3.65384100	-1.57389500
C	1.77263200	-0.35508700	-1.19191200	H	-1.35308800	-3.69539800	1.75283500
C	-1.77263300	-0.35510400	1.19193800	H	-0.99263500	1.62579000	-1.41398600
C	-0.21231300	-2.20805800	-1.92699100	H	-3.95376700	-2.85197400	0.78779100
C	-0.47427600	0.10666400	1.40903300	H	-0.65415500	3.01584800	4.24737400
C	-4.01465100	-0.67801700	0.56897200	H	0.72154400	4.13281200	4.24704600
C	5.43377400	-0.44845300	-0.07403700	H	0.91791300	2.47592500	3.65743200
C	-0.07534600	1.48733700	1.30647200	H	3.02697400	-2.16239500	1.74920500
C	0.21233200	-2.20808800	1.92693300	H	3.32679900	-3.32301600	3.04445100
C	0.87499200	2.61761600	-1.59157200	H	2.59048200	-1.76703800	3.41954400
C	2.99176800	0.35369600	-0.82557700	H	4.96982500	1.35821000	1.10105700
C	1.09721900	-2.64697700	-1.65158200	H	6.43009700	0.50981300	1.61275400
C	0.50690700	-0.85887800	1.75313000	H	4.83510100	-0.14822100	2.00324400
C	2.06775600	-1.73198000	-1.26160100	H	-6.30586900	-0.30430800	2.06839200
C	-1.24402800	-3.20823900	-2.46664500	H	-7.30691300	0.40588900	0.79264300
C	-2.06775100	-1.73200000	1.26160400	H	-5.88737600	1.26490700	1.38998400
C	1.24404600	-3.20828200	2.46656000	H	6.30588300	-0.30428200	-2.06827400
C	4.01463800	-0.67800400	-0.56890200	H	7.30690300	0.40590500	-0.79250000
C	-0.87499400	2.61759900	1.59161400	H	5.88737800	1.26492800	-1.38986300
C	3.16253300	1.71296600	-0.93554000	H	5.57335200	-2.36277600	0.97968500
C	-2.24802800	2.69339600	1.39109800	H	7.13046500	-1.61495400	0.61113400
C	-3.16254300	1.71295300	0.93560300	H	6.21426900	-2.40733800	-0.66978500
C	-0.50689300	-0.85885200	-1.75316900	H	-4.96988000	1.35820700	-1.10095600
C	-5.43379800	-0.44846500	0.07413700	H	-6.43016000	0.50980900	-1.61262600
C	3.45633200	-1.89466900	-0.85787000	H	-4.83517000	-0.14821700	-2.00315600
C	-0.14419000	3.80551700	2.26971800	H	0.46231100	-4.91341300	1.32238900
C	2.24802400	2.69340800	-1.39105700	H	2.11169100	-5.12566400	1.91182200
C	-1.09720200	-2.64700200	1.65154800	H	1.78695500	-4.05874700	0.53623700
C	-2.99178000	0.35368000	0.82564200	H	-5.57339100	-2.36277900	-0.97959600
C	0.07534500	1.48735200	-1.30641400	H	-7.13049900	-1.61496700	-0.61100700
C	-3.45633600	-1.89468600	0.85790400	H	-6.21427400	-2.40735600	0.66988700
C	0.14420500	3.80550700	-2.26974200	H	0.65429300	3.01573500	-4.24732900
C	0.23391600	3.32604200	3.69146700	H	-0.72140100	4.13270700	-4.24714800
C	2.62458000	-2.56927800	2.67781900	H	-0.91781500	2.47585100	-3.65746000
C	5.41295500	0.37089900	1.23429800	H	1.36666100	5.44434400	-1.45842700
C	-6.27844300	0.27755400	1.14396700	H	0.42668900	5.85576900	-2.88923600
C	6.27844000	0.27757300	-1.14384600	H	1.88523800	4.89052200	-3.05725200
C	6.12205300	-1.79077900	0.22765500	H	-1.36664300	5.44437900	1.45832000
C	-5.41300900	0.37089600	-1.23419300	H	-0.42666300	5.85582800	2.88914500
C	1.40730300	-4.39658700	1.49997200	H	-1.88523800	4.89061800	3.05717800
C	-6.12207900	-1.79079100	-0.22755000	H	1.85261300	3.39895500	1.41840200
C	-0.23381200	3.32596300	-3.69149600	H	1.64568500	5.00906300	2.09426100

C	1.01531900	5.06367200	-2.42035000
C	-1.01530700	5.06372700	2.42028300
C	1.14299400	4.21713000	1.53225600
C	0.74303200	-3.73128200	3.82995300
C	-2.62453000	-2.56919700	-2.67799500
C	-1.14307300	4.21712000	-1.53240100
C	-1.40738000	-4.39650100	-1.50002300
C	-0.74296200	-3.73130300	-3.82999300
H	0.99263300	1.62577500	1.41406100
H	1.35311000	-3.69537300	-1.75286600
H	1.51613900	-0.50256700	1.90868800
H	4.15471100	2.10143200	-0.73629100
H	-2.70995800	3.65383300	1.57392400
H	-4.15472300	2.10141800	0.73636500
H	-1.51612100	-0.50253900	-1.90874400
H	3.95376100	-2.85195900	-0.78776100
H	0.92908800	4.60047500	0.53618400
H	0.60722100	-2.90789400	4.53577900
H	1.46503300	-4.43306700	4.25887400
H	-0.21361300	-4.25018600	3.73791000
H	-3.02696900	-2.16229400	-1.74940900
H	-3.32675000	-3.32292000	-3.04465700
H	-2.59036500	-1.76696700	-3.41972800
H	-1.85260300	3.39886800	-1.41854900
H	-1.64580500	5.00893700	-2.09453400
H	-0.92938800	4.60064900	-0.53631700
H	-0.46239500	-4.91328600	-1.32228100
H	-2.11169400	-5.12561900	-1.91192700
H	-1.78717000	-4.05861300	-0.53636600
H	-0.60707300	-2.90794200	-4.53583700
H	-1.46497200	-4.43306700	-4.25893400
H	0.21365300	-4.25025000	-3.73788400

11-4. Relative energy of other possible σ-dimers to $\mathbf{1b}_2$

To estimate the relative energies of other possible σ-dimers, we optimized the structures of several dimers: (2*R*,2′*R*), (2*R*,2′*S*), (6*R*,6′*R*) = $\mathbf{1b}_2$, (6*R*,6′*S*), (8*R*,8′*R*), and (8*R*,8′*S*). In these dimers, **1b** dimerizes at the carbon atom with a large spin density on the azulene moiety. This optimization was performed using the RB3LYP-D3(BJ)/6-311G(d) level of theory. To identify the most stable conformer for each σ-dimer, we scanned the relaxed energies as a function of the dihedral angles around the formed σ-bond. All molecular geometries were fully optimized at the singlet states and were confirmed to have all positive vibrational frequencies for local minimum states at the same theoretical level. The results obtained were summarized in Table S32.

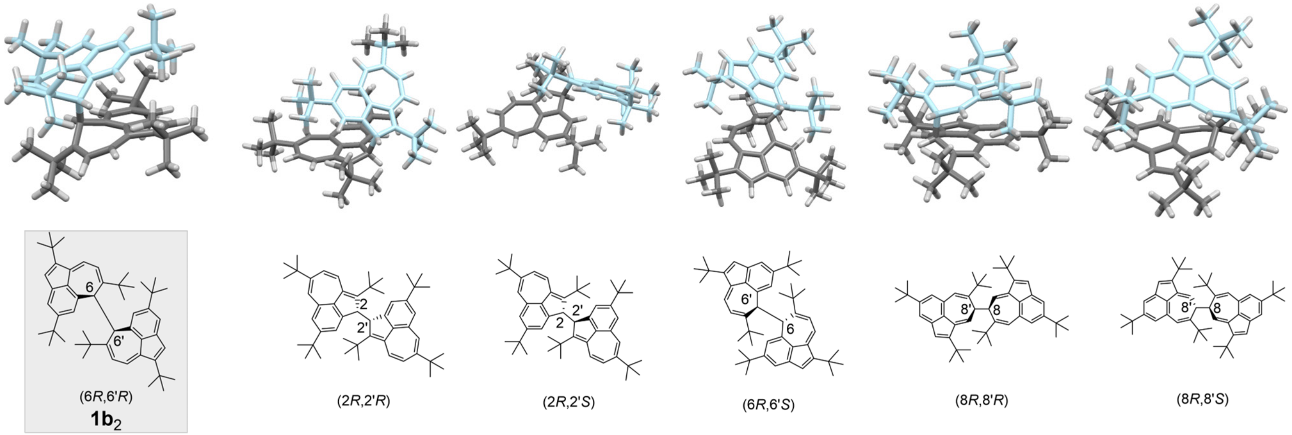

Table S34. Summary for energies of σ-dimers of **1b** at the RB3LYP-D3(BJ)/6-311G(d) level.

σ-Dimer	Dihedral angle / °	Total energy / Hartree	Relative energy to $\mathbf{1b}_2$ / kcal·mol^{-1}
(6*R*,6′*R*) = $\mathbf{1b}_2$	−168.6	−1945.7473746	0.00
(2*R*,2′*R*)	164.8	−1945.7134780	21.27
(2*R*,2′*S*)	70.0	−1945.7273814	12.55
(6*R*,6′*S*)	89.9	−1945.7247616	14.19
(8*R*,8′*R*)	26.4	−1945.7289985	11.53
(8*R*,8′*S*)	−163.1	−1945.7169165	19.11

Table S35. Optimized geometries for (2*R*,2′*R*) optimized at the RB3LYP-D3(BJ)/6-311G(d) level.

Atom	X	Y	Z	Atom	X	Y	Z
C	1.51799900	3.46415800	-1.29119800	C	-0.93306000	1.55587600	5.01638100
C	0.52163400	3.37707600	-2.46534400	C	-2.76484600	2.87386400	4.03512400
C	2.89321100	3.92828200	-1.80968200	C	-2.36265100	3.00044000	0.80872400
H	-0.45316400	3.03156400	-2.11847700	C	-2.30204000	0.29286500	-0.88026900
H	0.39354600	4.35834900	-2.93388300	C	-1.64023900	-2.07752700	-0.64779500
H	0.86463200	2.68189400	-3.23514700	H	-0.80714300	-2.60841400	1.26827300
C	0.99848000	4.51770900	-0.30150200	H	-0.67090300	-3.14077400	3.48304100
C	1.64023900	2.07752700	-0.64779500	H	0.38624800	-4.15597700	4.46468500
H	2.81376100	4.91821900	-2.26900100	H	0.57596300	-4.11665600	2.71096900
H	3.61734700	3.99018800	-0.99307700	H	1.60202000	-0.71439200	5.21031300
H	3.29847000	3.24790100	-2.56139700	H	0.98556800	-2.21970700	5.88314400
H	0.94833000	5.49232600	-0.79422900	H	-0.08556300	-1.17573800	4.96437100
H	1.65606200	4.62043800	0.56586800	H	2.75913400	-3.47711500	4.94819800
H	-0.00538100	4.27646800	0.05193100	H	3.46475500	-2.04743800	4.18034200
C	2.15944500	1.01346800	-1.38601500	H	3.15843100	-3.49388600	3.23223100
C	1.21843500	1.81859700	0.66181000	H	0.67090300	3.14077400	3.48304100
C	2.30204000	-0.29286500	-0.88026900	H	-0.38624800	4.15597700	4.46468500
H	2.48270300	1.18827800	-2.40678100	H	-0.57596300	4.11665600	2.71096900
C	1.31115200	0.53364400	1.17625500	H	-1.60202000	0.71439200	5.21031300
H	0.80714300	2.60841400	1.26827300	H	-0.98556800	2.21970700	5.88314400
C	1.86332700	-0.51475100	0.43523500	H	0.08556300	1.17573800	4.96437100
C	2.89321100	-1.28047500	-1.76554700	H	-2.75913400	3.47711500	4.94819800
C	0.80499900	-0.01174100	2.49006900	H	-3.46475500	2.04743800	4.18034200
C	1.88548100	-1.72013700	1.27916200	H	-3.15843100	3.49388600	3.23223100
C	3.15435000	-2.60051100	-1.60162300	C	-2.87849400	3.35774200	-0.39240900
H	3.16653700	-0.85520300	-2.72595800	H	-2.26128600	3.82034800	1.50736100
C	-0.80499900	0.01174100	2.49006900	C	-2.89321100	1.28047500	-1.76554700
C	1.36246500	-1.42951400	2.51341100	C	-2.15944500	-1.01346800	-1.38601500
H	1.14080600	0.58010000	3.34180500	C	-1.51799900	-3.46415800	-1.29119800
C	2.36265100	-3.00044000	0.80872400	C	-3.15435000	2.60051100	-1.60162300
C	3.83232000	-3.33811200	-2.79084900	H	-3.11827500	4.41030100	-0.45535600
C	2.87849400	-3.35774200	-0.39240900	H	-3.16653700	0.85520300	-2.72595800
C	-1.36246500	1.42951400	2.51341100	H	-2.48270300	-1.18827800	-2.40678100
C	-1.31115200	-0.53364400	1.17625500	C	-0.52163400	-3.37707600	-2.46534400
H	-1.14080600	-0.58010000	3.34180500	C	-2.89321100	-3.92828200	-1.80968200
C	1.34476400	-2.32992300	3.75043300	C	-0.99848000	-4.51770900	-0.30150200
H	2.26128600	-3.82034800	1.50736100	C	-3.83232000	3.33811200	-2.79084900
C	5.27104300	-2.80290900	-2.94519100	H	0.45316400	-3.03156400	-2.11847700
C	3.05110300	-3.09526300	-4.09923300	H	-0.39354600	-4.35834900	-2.93388300
C	3.91662600	-4.86291300	-2.60036100	H	-0.86463200	-2.68189400	-3.23514700
H	3.11827500	-4.41030100	-0.45535600	H	-2.81376100	-4.91821900	-2.26900100
C	-1.34476400	2.32992300	3.75043300	H	-3.61734700	-3.99018800	-0.99307700
C	-1.88548100	1.72013700	1.27916200	H	-3.29847000	-3.24790100	-2.56139700
C	-1.86332700	0.51475100	0.43523500	H	-0.94833000	-5.49232600	-0.79422900
C	-1.21843500	-1.81859700	0.66181000	H	-1.65606200	-4.62043800	0.56586800
C	0.35436100	-3.50440600	3.58601000	H	0.00538100	-4.27646800	0.05193100
C	0.93306000	-1.55587600	5.01638100	C	-5.27104300	2.80290900	-2.94519100
C	2.76484600	-2.87386400	4.03512400	C	-3.05110300	3.09526300	-4.09923300
H	5.78908600	-3.31427600	-3.76271800	C	-3.91662600	4.86291300	-2.60036100
H	5.84543200	-2.96073300	-2.02850800	H	-5.78908600	3.31427600	-3.76271800
H	5.27876700	-1.73215000	-3.15903200	H	-5.84543200	2.96073300	-2.02850800
H	2.02421300	-3.45723300	-4.01455500	H	-5.27876700	1.73215000	-3.15903200
H	3.53048200	-3.62527600	-4.92741100	H	-2.02421300	3.45723300	-4.01455500
H	3.00843800	-2.04011000	-4.37239400	H	-3.53048200	3.62527600	-4.92741100
H	4.35552100	-5.31299900	-3.49410400	H	-3.00843800	2.04011000	-4.37239400
H	2.93140100	-5.31451800	-2.45728800	H	-4.35552100	5.31299900	-3.49410400
H	4.55081800	-5.14507800	-1.75678100	H	-2.93140100	5.31451800	-2.45728800
C	-0.35436100	3.50440600	3.58601000	H	-4.55081800	5.14507800	-1.75678100

Table S36. Optimized geometries for (2*R*,2′*S*) optimized at the RB3LYP-D3(BJ)/6-311G(d) level.

Atom	X	Y	Z	Atom	X	Y	Z
C	-1.01014900	0.33246700	-0.65366800	H	-2.01679800	-1.78815100	3.24884000
C	-0.72072000	1.69226100	-0.07367400	H	-0.67605100	-2.28125500	2.21133900
C	-2.37984300	0.07745400	-0.56504400	H	-1.92862000	-1.23236700	1.57446900
C	0.34806400	1.60635200	1.12376600	C	3.13787500	-2.37558600	1.56258200
C	-2.10532600	2.24921500	0.22446200	H	1.31184500	-2.25349100	2.48439100
H	-0.24505500	2.32453000	-0.82794000	C	4.51562300	-0.83533900	0.16013900
C	-0.11862800	-0.60427100	-1.15397600	C	4.18987500	1.44012100	-0.66419900
C	-2.88741600	-1.16935300	-0.97172000	C	4.07676000	3.80887600	-1.65787900
C	-3.04171700	1.27981300	-0.02760400	H	2.43671800	-3.25210300	-2.60012900
C	0.44154700	0.27597300	1.87981000	H	2.24284000	-2.10248900	-1.28437400
C	1.72004000	1.72415800	0.49579000	H	1.84805200	-1.62473000	-2.94154800
H	0.14856600	2.42957900	1.80976000	H	0.56513800	-4.30403100	-3.74045700
C	-2.35782500	3.72586400	0.54753700	H	-1.11197100	-3.97268600	-3.31187100
C	-0.58239100	-1.86304800	-1.54728000	H	-0.16077400	-2.74045800	-4.14277900
H	0.92949500	-0.35653400	-1.22579100	H	1.06715000	-4.89640000	-1.30043700
C	-4.28279200	-1.58238400	-0.94102800	H	0.75518000	-3.71025700	-0.02404100
C	-1.95363900	-2.11232000	-1.44136100	H	-0.59539600	-4.52087200	-0.82669800
C	-4.46735000	1.35845300	0.19825500	C	-7.71722700	-0.80284300	-1.51636900
C	-0.56202300	-0.19901300	2.93243000	C	-6.71291000	-3.04620100	-1.16977300
C	1.61056300	-0.35074200	1.53687600	C	-7.37533100	-1.71166800	0.81538300
C	2.42415800	0.52940000	0.68248200	C	4.31282700	-2.02438200	0.77208700
C	2.24010400	2.76425600	-0.25173900	H	3.15476200	-3.38679900	1.95229600

C	-2.95715100	3.92318900	1.95702800	H	5.43832700	-0.72224300	-0.39460300
C	-3.30901700	4.33060400	-0.51350900	H	5.16281800	1.30723700	-1.12035700
C	-1.06521700	4.56435100	0.47780900	C	4.21018900	5.04549700	-0.74553800
C	0.36815000	-2.96328400	-2.03544600	C	5.45939000	3.49551100	-2.24737600
C	-5.39604800	-0.92802600	-0.53665400	C	3.11920200	4.13392600	-2.82293500
H	-4.41658200	-2.59185000	-1.30866700	H	-8.69896300	-1.28195900	-1.57330600
H	-2.33631200	-3.08441700	-1.73402800	H	-7.87099800	0.21568200	-1.15589900
C	-5.42982900	0.43137600	-0.00886200	H	-7.31227500	-0.73785700	-2.52946500
H	-4.82240400	2.28426600	0.63158200	H	-7.71898300	-3.47205300	-1.19249300
C	-1.59244700	0.89283300	3.26018500	H	-6.09443000	-3.70476000	-0.55502800
C	0.17124300	-0.50048900	4.26235600	H	-6.32696100	-3.06655600	-2.19190300
C	-1.33608000	-1.45037100	2.46102700	H	-8.35224400	-2.20277300	0.78291700
C	2.02174000	-1.68985100	1.89579800	H	-6.72400900	-2.29482100	1.47138200
C	3.68333100	0.35851300	0.09004700	H	-7.51725300	-0.73242800	1.27575100
C	3.50740300	2.64033500	-0.84486700	C	5.35834800	-3.15338800	0.65106400
H	1.66910200	3.67718700	-0.37520100	H	4.61308900	5.89425600	-1.30623800
H	-2.24900400	3.62206700	2.73011700	H	3.24682000	5.34912300	-0.33034300
H	-3.20020300	4.97784100	2.11886700	H	4.88255800	4.84076800	0.09144900
H	-3.86845900	3.34407100	2.10834400	H	5.82150800	4.35492500	-2.81717200
H	-3.45484800	5.39870800	-0.32448600	H	5.42771900	2.64062400	-2.92772000
H	-2.88373000	4.21897500	-1.51400900	H	6.19606700	3.28480300	-1.46797600
H	-4.28967300	3.85817400	-0.52360700	H	2.12762700	4.42086800	-2.46644900
H	-0.60223600	4.51244800	-0.51027800	H	2.99838400	3.26924600	-3.48045800
H	-1.30409700	5.61357200	0.66874200	H	3.50918700	4.96368100	-3.41987900
H	-0.32336200	4.26475100	1.21785700	C	4.71098100	-4.37749400	-0.03369400
C	1.80303700	-2.44588700	-2.22125200	C	5.86043400	-3.54660300	2.05731200
C	-0.11789500	-3.52694700	-3.38482200	C	6.58634900	-2.75788300	-0.18206500
C	0.39695500	-4.09135000	-0.98300100	H	5.43804100	-5.18997800	-0.12156900
C	-6.77536300	-1.61928700	-0.60475200	H	4.36656600	-4.11913100	-1.03778300
H	-6.41955700	0.76679700	0.27973400	H	3.85300500	-4.76363600	0.51896000
H	-1.10918300	1.79169000	3.65247300	H	6.60283800	-4.34657900	1.98412200
H	-2.27979400	0.53170300	4.02979100	H	6.32994000	-2.69241300	2.55181100
H	-2.18336400	1.17178200	2.39257100	H	5.05803200	-3.90352500	2.70498800
H	0.89725000	-1.30783600	4.18573600	H	7.28290600	-3.59871600	-0.22408700
H	-0.55576600	-0.78069100	5.03072600	H	7.12268500	-1.91139000	0.25381700
H	0.70608700	0.38616300	4.61238700	H	6.31991800	-2.50400400	-1.21098100

Table S37. Optimized geometries for (6*R*,6′*S*) optimized at the RB3LYP-D3(BJ)/6-311G(d) level.

Atom	X	Y	Z	Atom	X	Y	Z
C	0.25349000	-0.35036900	0.38422500	C	3.77377100	-4.12746500	-0.08375800
C	-0.77546000	-1.11190100	-0.50259100	C	4.35742000	-1.65758500	0.01556300
C	-0.03506000	0.10265600	1.81214300	C	4.78197100	0.91659900	0.18893000
C	-0.38139300	-0.94861500	-1.96991600	C	3.97055100	1.97338400	0.47659600
C	-2.18648100	-0.58004300	-0.36835300	C	-5.20286200	-2.54195200	2.07169100
H	-0.78277500	-2.16447700	-0.24467900	C	-5.89953000	-2.86538200	-0.32679000
C	1.65492500	-0.92342400	0.38155100	C	-6.95964200	-1.08999900	1.06271200
H	0.37756100	0.59538000	-0.13193800	H	-5.65078000	0.97418800	0.56921700
C	-0.79092000	-0.69197800	2.88919000	H	-4.31557500	3.49524000	-0.02543200
C	0.52833300	1.29443900	2.14585500	C	-1.81516100	4.50112100	-1.14329900
C	-0.17847900	-2.17542300	-2.88201300	C	4.76141400	-4.49298400	1.04397200
C	-0.21716700	0.28948700	-2.50067500	C	2.58083100	-5.09080200	0.00065200
C	-3.27014800	-1.38965900	0.00366000	C	4.45871700	-4.33427500	-1.45042400
C	-2.43325200	0.77639900	-0.53393000	H	5.39126300	-1.93239300	-0.16405200
C	2.02680200	-2.27309200	0.24368500	H	5.83953100	0.97156600	-0.02956900
C	2.67180400	0.01591700	0.45399400	C	4.40035200	3.42715300	0.56589600
C	-2.17291400	-0.05525100	3.14161500	H	-5.00437300	-1.82200600	2.86928400
C	-0.96293000	-2.17212200	2.51090800	H	-5.98684800	-3.22382000	2.41476700
C	0.01017800	-0.67366700	4.21160700	H	-4.29341200	-3.12762200	1.92338200
C	1.59467600	2.00739800	1.48303700	H	-6.69366400	-3.55448400	-0.02414900
H	0.25244800	1.73860500	3.09881900	H	-6.20695900	-2.37915500	-1.25610900
C	1.31238500	-2.29073600	-3.26524900	H	-5.00987900	-3.46046600	-0.54278100
C	-0.62014100	-3.49203800	-2.22165000	H	-7.72591700	-1.81139000	1.35747000
C	-1.02361900	-2.01412200	-4.16475300	H	-7.32798600	-0.55947700	0.18094800
C	-0.60127000	1.58382700	-1.99123300	H	-6.85947200	-0.36914200	1.87801900
H	0.20429500	0.34146800	-3.50090600	C	-1.78765900	4.75925800	-2.66619800
C	-4.52671600	-0.86572400	0.32798800	C	-0.39941700	4.66670200	-0.55419900
H	-3.10561300	-2.45772100	0.08951400	C	-2.72611400	5.56270300	-0.50504700
C	-3.66580200	1.34284600	-0.14407300	H	5.07242200	-5.53847200	0.95706000
C	-1.61101100	1.82870400	-1.10669000	H	4.29875700	-4.35586600	2.02469200
C	3.35877400	-2.65769300	0.07222200	H	5.66062500	-3.87468500	1.01301800
H	1.25168300	-3.02507500	0.23359600	H	2.05077800	-5.00181200	0.95239400
C	4.01651900	-0.33066900	0.20181000	H	2.93328000	-6.12191900	-0.08303600
C	2.60274200	1.43578500	0.75147000	H	1.86488100	-4.92616700	-0.80770300
H	-2.06717200	0.97652200	3.48491300	H	3.77968500	-4.08510500	-2.26931600
H	-2.70997100	-0.61463300	3.91450200	H	4.76583300	-5.37764200	-1.57002100
H	-2.78435300	-0.04607300	2.24271100	H	5.34969300	-3.71260500	-1.55787500
H	-0.00204500	-2.63544800	2.27616000	C	4.43985600	3.88860900	2.04009400
H	-1.39479100	-2.71577700	3.35524700	C	3.43198900	4.31836900	-0.23778900
H	-1.63366600	-2.30759200	1.66897500	C	5.81105900	3.61047500	-0.01874200
H	-0.49611000	-1.28844900	4.96181900	H	-1.44553600	5.77842000	-2.87046300
H	1.01614100	-1.07335200	4.06319100	H	-2.78709900	4.64423500	-3.09252300
H	0.10790600	0.33027800	4.62662100	H	-1.12357700	4.07511500	-3.19440400
H	1.70784400	3.04180900	1.78634500	H	-0.02334300	5.67698500	-0.74051900
H	1.64688000	-1.41553400	-3.82591300	H	-0.41186800	4.50110400	0.52612400
H	1.47553700	-3.17299500	-3.89249700	H	0.31072200	3.95979600	-0.98138700

H	1.94134100	-2.37479300	-2.37925500	H	-2.32137100	6.56152900	-0.68810300
H	-0.51610000	-4.30981900	-2.93895600	H	-2.80346700	5.42342300	0.57604900
H	-1.66822900	-3.45824400	-1.91316600	H	-3.73462200	5.53536100	-0.92507500
H	-0.01454700	-3.75019200	-1.35313400	H	4.77959800	4.92704100	2.10242500
H	-0.90521700	-2.89060000	-4.80890600	H	5.13113900	3.26934300	2.61661100
H	-2.08357400	-1.90965000	-3.92039000	H	3.46411900	3.82872600	2.52215500
H	-0.72935200	-1.13760200	-4.74340200	H	3.76235700	5.36094500	-0.20885800
H	-0.14082700	2.42440100	-2.49656000	H	3.39620600	4.00425200	-1.28429800
C	-5.63971000	-1.82209500	0.77874300	H	2.41517500	4.28051600	0.15060800
C	-4.70552400	0.53033800	0.28371700	H	6.09336900	4.66612800	0.01131400
C	-3.54440100	2.79172700	-0.30761900	H	5.85810800	3.27853100	-1.05883000
C	-2.33234000	3.10624700	-0.84422100	H	6.56028700	3.05488100	0.55036300

Table S38. Optimized geometries for (8*R*,8′*R*) optimized at the RB3LYP-D3(BJ)/6-311G(d) level.

Atom	X	Y	Z	Atom	X	Y	Z
C	-2.65383300	0.04742500	-0.81337600	C	2.59778300	1.53123400	1.69749900
C	-2.65383800	-0.04744500	0.81337300	H	1.13612000	3.10128200	1.60800500
C	-2.39339200	-1.29115000	-1.48539200	C	2.77595600	0.14141900	1.78436300
C	-2.39340400	1.29113100	1.48539000	C	-0.92278800	-4.44276300	0.80457200
C	-1.87464900	-1.22242300	1.33077100	C	-1.31493400	-3.97245700	3.25925500
H	-3.68893100	-0.30356500	1.03056300	C	0.86471500	-4.88174800	2.47529100
C	-1.87465000	1.22240900	-1.33076900	H	2.38595100	-2.78839500	2.12660400
H	-3.68892500	0.30354000	-1.03057400	C	2.77596300	-0.14140600	-1.78437400
C	-3.65715400	-2.05480000	-1.92767100	C	-0.92273800	4.44277800	-0.80452500
C	-1.17001400	-1.81060100	-1.69823900	C	-1.31500800	3.97244300	-3.25918300
C	-3.65717000	2.05478200	1.92765800	C	0.86468200	4.88174200	-2.47533700
C	-1.17003000	1.81059200	1.69822900	H	2.38593900	2.78840600	-2.12661800
C	-0.57173100	-1.29192300	1.64278600	C	3.78059200	-2.50965100	-1.75296500
H	-2.45670300	-2.13367200	1.37978700	C	3.78057300	2.50967000	1.75296500
C	-0.57173100	1.29191800	-1.64278400	H	3.76409200	-0.28647700	1.89435800
H	-2.45671000	2.13365400	-1.37978700	H	-1.29180300	-5.46393800	0.94089500
C	-3.36025100	-3.47482100	-2.43529900	H	-0.18652400	-4.45286600	-0.00211000
C	-4.67920500	-2.18387000	-0.77851800	H	-1.75428400	-3.82357900	0.47320800
C	-4.30898400	-1.27308900	-3.09169000	H	-1.66755300	-4.99705100	3.41161700
C	0.15137900	-1.20358000	-1.58149300	H	-0.86435100	-3.62671600	4.19291600
H	-1.12339700	-2.82125200	-2.08572600	H	-2.18464400	-3.34493100	3.06465900
C	-4.67934400	2.18357100	0.77858300	H	0.47653600	-5.89313100	2.62151900
C	-4.30883500	1.27325100	3.09189200	H	1.36189900	-4.57757100	3.39961000
C	-3.36030300	3.47491600	2.43499100	H	1.61691300	-4.92786500	1.68404600
C	0.15136700	1.20357900	1.58148300	H	3.76409600	0.28649500	-1.89436800
H	-1.12341800	2.82124500	2.08571200	H	-1.29175500	5.46395300	-0.94084000
C	0.36984000	-0.17175400	1.60606600	H	-0.18643500	4.45288500	0.00212100
C	0.23895300	-2.51131000	1.93029000	H	-1.75422100	3.82360200	-0.47311300
C	0.36984500	0.17175300	-1.60607200	H	-1.66763800	4.99703500	-3.41153600
C	0.23894500	2.51131100	-1.93028300	H	-0.86447000	3.62669600	-4.19286300
H	-2.71461700	-3.46900400	-3.31585800	H	-2.18470500	3.34491600	-3.06453700
H	-4.29516600	-3.96282600	-2.72186700	H	0.47649700	5.89312500	-2.62155100
H	-2.88782100	-4.09295900	-1.66824400	H	1.36181700	4.57755900	-3.39968000
H	-5.56129400	-2.73026400	-1.12256800	H	1.61692100	4.92786400	-1.68413100
H	-5.02809300	-1.21578100	-0.41515600	C	3.79874200	-3.38203400	-0.48374900
H	-4.26081900	-2.73487200	0.06738000	C	5.13223800	-1.78551900	-1.84482500
H	-5.21247000	-1.78539700	-3.43593600	C	3.63629400	-3.41539700	-2.99315900
H	-4.59770200	-0.26046100	-2.80147500	C	3.63625900	3.41541600	2.99315700
H	-3.61829200	-1.19332700	-3.93396800	C	5.13222200	1.78554400	1.84484000
C	1.29417900	-2.02818800	-1.61902100	C	3.79873100	3.38205100	0.48374900
H	-4.26108000	2.73444900	-0.06745600	H	4.60811100	-4.11673700	-0.53504300
H	-5.56143800	2.72996500	1.12262100	H	2.86066500	-3.92286500	-0.34835400
H	-5.02819700	1.21539100	0.41543200	H	3.95529800	-2.76559100	0.40347100
H	-5.21230300	1.78558200	3.43614700	H	5.94386100	-2.51753500	-1.85819600
H	-3.61804400	1.19367300	3.93410800	H	5.21513100	-1.18808400	-2.75614800
H	-4.59754200	0.26055800	2.80189600	H	5.29716100	-1.12616800	-0.98850300
H	-4.29521500	3.96290600	2.72159200	H	4.48155900	-4.10687400	-3.06480000
H	-2.88801700	4.09294900	1.66776200	H	3.60549100	-2.81861600	-3.90824900
H	-2.71455300	3.46930300	3.31546600	H	2.72192800	-4.01101700	-2.95534700
C	1.29416300	2.02819300	1.61901100	H	4.48152000	4.10689900	3.06480300
C	1.66797700	-0.69211100	1.76320100	H	2.72189000	4.01103200	2.95533600
C	-0.28378700	-3.92574800	2.11145300	H	3.60545300	2.81863700	3.90824700
C	1.54286900	-2.13398400	1.96234500	H	5.94384100	2.51756500	1.85821600
C	1.66797900	0.69211800	-1.76321000	H	5.29715600	1.12619200	0.98852100
C	-0.28380300	3.92574800	-2.11143200	H	5.21511000	1.18811300	2.75616500
C	1.54286200	2.13399100	-1.96235400	H	4.60809700	4.11675800	0.53504900
C	2.59779700	-1.53122100	-1.69750800	H	3.95529900	2.76560700	-0.40346900
H	1.13614200	-3.10127700	-1.60801400	H	2.86065400	3.92287700	0.34834400

Table S39. Optimized geometries for (8*R*,8′*S*) optimized at the RB3LYP-D3(BJ)/6-311G(d) level.

Atom	X	Y	Z	Atom	X	Y	Z
C	2.70554500	-0.19921900	3.26932500	C	2.39562900	-4.42507600	-0.84397000
C	2.32299300	-0.61735400	4.70053200	C	0.18530000	-3.38800400	-1.63308400
C	3.82801400	-1.15385200	2.81155800	C	0.12233700	5.11057600	1.57053200
H	1.52967000	0.01357800	5.10641300	C	-0.01819100	4.47877600	-0.87400200
H	3.19407200	-0.52548700	5.35467400	C	-1.98243800	5.48291000	0.28686200
H	1.99131200	-1.65751200	4.74757600	H	-3.41091900	3.26057200	0.55053100
C	3.25424500	1.24147500	3.34411100	C	-5.67511800	-1.30891500	2.86338200

C	1.49329200	-0.21030500	2.33168700	C	-4.14250700	-3.24869600	2.55247900
H	4.63120800	-1.16588000	3.55415700	C	-5.11911200	-2.13397900	0.54872300
H	4.27477900	-0.86200500	1.86281400	H	-4.64691600	0.71664700	1.15240700
H	3.45035900	-2.17271400	2.71080400	C	-1.84470500	-0.32580600	-2.72513600
H	4.10863100	1.28676100	4.02583800	H	-0.93972900	1.61978100	-2.83246000
H	3.58956500	1.60468400	2.37291800	C	-1.61070800	-1.68066000	-2.42327200
H	2.48876800	1.93004800	3.70851600	C	2.67837800	-4.43037900	0.67372400
C	1.49544700	0.43100600	0.94123900	C	3.72021800	-4.30517800	-1.62618300
C	0.28617300	-0.66839200	2.72322400	C	1.75083300	-5.77345100	-1.20482300
C	2.72307500	0.24182100	-0.00181300	H	-0.35952800	-4.31247000	-1.76737700
C	0.96215600	1.83464800	1.02060400	H	-0.41528600	5.16743100	2.52038000
H	0.70564800	-0.10979500	0.43048500	H	0.45045900	6.12019900	1.30581800
C	-0.99789100	-0.36859800	2.08233300	H	1.01208900	4.50198900	1.73407200
H	0.19253000	-1.20260900	3.66243900	H	0.84497700	3.81637900	-0.82822800
C	2.64580300	1.16150200	-1.22245300	H	0.32988700	5.47764300	-1.15524400
C	2.78863300	-1.21070400	-0.42875100	H	-0.66865200	4.10895600	-1.67080000
H	3.63836800	0.47820200	0.53252000	H	-1.63057000	6.48849300	0.04159400
C	-0.37066300	1.99234300	1.18053900	H	-2.57944500	5.54880500	1.19984200
H	1.62203600	2.67046800	0.83644400	H	-2.63735700	5.15638700	-0.52476200
C	-2.10051200	-1.24036600	2.25358400	H	-6.51381100	-2.00776500	2.93686400
C	-1.25426100	0.86539500	1.47484700	H	-6.06459100	-0.36721500	2.47165300
C	3.86534700	2.04683000	-1.50181900	H	-5.30134500	-1.11938000	3.87286700
C	1.55366300	1.17349600	-2.00743600	H	-5.00500600	-3.91915000	2.58964000
C	1.78387600	-1.81940600	-1.09387100	H	-3.37503400	-3.73237900	1.94318800
H	3.63409000	-1.78688300	-0.07661400	H	-3.75828600	-3.15121700	3.57110800
C	-1.25580500	3.14388500	0.84662300	H	-5.95055400	-2.84500800	0.57431800
C	-3.39409700	-0.90590400	1.87098400	H	-4.34171000	-2.53895800	-0.10424600
H	-1.90250400	-2.20459900	2.70467000	H	-5.48374300	-1.20993900	0.09516400
C	-2.58243200	1.27466300	1.20854300	C	-3.18697600	0.17182300	-3.27995300
C	3.70693600	2.88399700	-2.77994600	H	-2.37387900	-2.42654300	-2.60593500
C	4.13311800	3.02055000	-0.33589800	H	3.29304500	-5.29518900	0.94166700
C	5.09664000	1.13113600	-1.68764800	H	1.74484400	-4.48571300	1.23879000
C	0.44962400	0.22013800	-2.00927100	H	3.20511500	-3.53457600	0.99757200
H	1.48346500	1.92304500	-2.78743800	H	4.38740000	-5.13501000	-1.37464300
C	0.61882000	-1.11545900	-1.63690300	H	3.53536400	-4.33350000	-2.70298400
C	1.47258300	-3.27786200	-1.21008000	H	4.24600300	-3.37498300	-1.40851900
C	-0.78656400	4.53421700	0.46543500	H	2.43979700	-6.59012100	-0.97409900
C	-2.53627900	2.68511000	0.82129600	H	1.50850400	-5.82961200	-2.26889300
C	-4.56378900	-1.89273800	1.96825500	H	0.83354900	-5.94621800	-0.63644500
C	-3.63527300	0.39837300	1.38041600	C	-3.76050700	1.24391400	-2.33225200
H	3.54461300	2.25521400	-3.65810700	C	-4.22224700	-0.95607500	-3.40276100
H	4.61460300	3.46787400	-2.95255000	C	-2.97218100	0.78225300	-4.67972400
H	2.87560200	3.58933700	-2.70523600	H	-4.71564200	1.61939400	-2.71298700
H	5.03881700	3.60135400	-0.53096000	H	-3.08441000	2.09350600	-2.22093000
H	4.27959800	2.50662200	0.61505600	H	-3.92240400	0.82844900	-1.33682900
H	3.30866300	3.72727600	-0.21454000	H	-5.16474800	-0.55149700	-3.78064900
H	5.98315100	1.73048800	-1.91395400	H	-3.89927000	-1.73483000	-4.09862400
H	5.31752800	0.54830700	-0.79076500	H	-4.42878000	-1.42303600	-2.43707200
H	4.93708900	0.43082200	-2.51057500	H	-3.92219100	1.13352500	-5.09396300
C	-0.80005000	0.58255700	-2.54791200	H	-2.55375100	0.04306400	-5.36783100
C	-0.38082500	-2.06811300	-1.91316200	H	-2.28844200	1.63307700	-4.65081700

12. References

[1] S. Stoll, A. Schweiger, “EasySpin, a comprehensive software package for spectral simulation and analysis in EPR” *J. Magn. Reson.* **2006**, *178*, 42–55.

[2] O. V. Dolomanov, L. J. Bourhis, R. J. Gildea, J. A. K. Howard, H. Puschmann, “OLEX2 : a complete structure solution, refinement and analysis program” *J. Appl. Crystallogr.* **2009**, *42*, 339–341.

[3] Z. Zhao, W. Huan, C. Sun, M. E. El-Khouly, B. Zhang, Y. Chen, “Proton-responsive azulene-based conjugated polymer with nonvolatile memory effects” *New J. Chem.* **2022**, *46*, 3800–3805.

[4] S. Ito, N. Morita, T. Asao, “Syntheses of Azulene Analogues of Triphenylmethyl Cation: Extremely Stable Hydrocarbon Carbocations and the First Example of a One-Ring Flip as the Threshold Rotation Mechanism for Molecular Propellers” *Bull. Chem. Soc. Jpn.* **1995**, *68*, 1409–1436.

[5] M. J. Frisch, G. W. Trucks, H. B. Schlegel, G. E. Scuseria, M. A. Robb, J. R. Cheeseman, G. Scalmani, V. Barone, G. A. Petersson, H. Nakatsuji, X. Li, M. Caricato, A. V. Marenich, J. Bloino, B. G. Janesko, R. Gomperts, B. Mennucci, H. P. Hratchian, J. V. Ortiz, A. F. Izmaylov, J. L. Sonnenberg, D. Williams-Young, F. Ding, F. Lipparini, F. Egidi, J. Goings, B. Peng, A. Petrone, T. Henderson, D. Ranasinghe, V. G. Zakrzewski, J. Gao, N. Rega, G. Zheng, W. Liang, M. Hada, M. Ehara, K. Toyota, R. Fukuda, J. Hasegawa, M. Ishida, T. Nakajima, Y. Honda, O. Kitao, H. Nakai, T. Vreven, K. Throssell, J. J. A. Montgomery, J. E. Peralta, F. Ogliaro, M. J. Bearpark, J. J. Heyd, E. N. Brothers, K. N. Kudin, V. N. Staroverov, T. A. Keith, R. Kobayashi, J. Normand, K. Raghavachari, A. P. Rendell, J. C. Burant, S. S. Iyengar, J. Tomasi, M. Cossi, J. M. Millam, M. Klene, C. Adamo, R. Cammi, J. W. Ochterski, R. L. Martin, K. Morokuma, O. Farkas, J. B. Foresman, D. J. Fox, *Gaussian 16, Revision C.01*, Gaussian, Inc., Wallingford CT, **2016**.

[6] D. Geuenich, K. Hess, F. Köhler, R. Herges, “Anisotropy of the Induced Current Density (ACID), a General Method To Quantify and Visualize Electronic Delocalization” *Chem. Rev.* **2005**, *105*, 3758–3772.
